# Supplementary material for: Harnessing PROTAC technology to combat stress hormone receptor activation
Source: Nat Commun. 2023 Dec 9;14:8177. doi: 10.1038/s41467-023-44031-2 (PMC10710461; doi:10.1038/s41467-023-44031-2)
Supplement: Supplementary file 1 — Supplementary information [file 41467_2023_44031_MOESM1_ESM.pdf]

Supplementary Information

Harnessing PROTAC technology to combat stress hormone receptor activation

Mahshid Gazorpak*[1,2], Karina M. Hugentobler*[3], Dominique Paul[4], Pierre-Luc Germain[1,4,5], Miriam Kretschmer[1,2], Iryna Ivanova[1], Selina Frei[1], Kei Mathis[1], Remo Rudolf[1], Sergio Mompart Barrenechea[1], Vincent Fischer[1,2], Xiaohan Xue[6], Aleksandra L. Ptaszek[7], Julian Holzinger[7], Mattia Privitera[8], Andreas Hierlemann[6], Onno C. Meijer[9], Robert Konrat[10], Erick M. Carreira[3], Johannes Bohacek[2,8], Katharina Gapp[#1,2]

[1]Laboratory of Epigenetics and Neuroendocrinology, Institute for Neuroscience, Department of Health Science and Technology, ETH Zurich, 8057 Zurich, Switzerland

[2]Neuroscience Center Zurich, ETH Zurich and University of Zurich, Switzerland

[3]Laboratory of Organic Chemistry, Department of Chemistry and Applied Biosciences, ETH Zurich, Zurich, Switzerland

[4]Lab of Statistical Bioinformatics, University of Zürich, Switzerland,

[5]Computational Neurogenomics, Institute for Neuroscience, Department of Health Science and Technology, ETH Zurich, 8057 Zurich, Switzerland

[6]Bio Engineering Laboratory, Department of Biosystems Science and Engineering, ETH Zurich, 4056 Basel, Switzerland

[7]Christian Doppler Laboratory for High-Content Structural Biology and Biotechnology, Max Perutz Laboratories, Department of Structural and Computational Biology, University of Vienna, Campus Vienna Biocenter 5, 1030 Vienna, Austria

[8] Laboratory of Molecular and Behavioral Neuroscience, Institute for Neuroscience, Department of Health Science and Technology, ETH Zurich, 8057 Zurich, Switzerland

[9] Department of Medicine, Division of Endocrinology, Leiden University Medical Center, 2300 RA Leiden, the Netherlands

[10] Department of Structural and Computational Biology, University of Vienna, Campus Vienna Biocenter 5, 1030 Vienna, Austria

* These authors contributed equally

#Corresponding: Prof. Katharina Gapp (katharina.gapp@hest.ethz.ch)

This file contains:

A) Supplementary Figures

B) Supplementary Methods

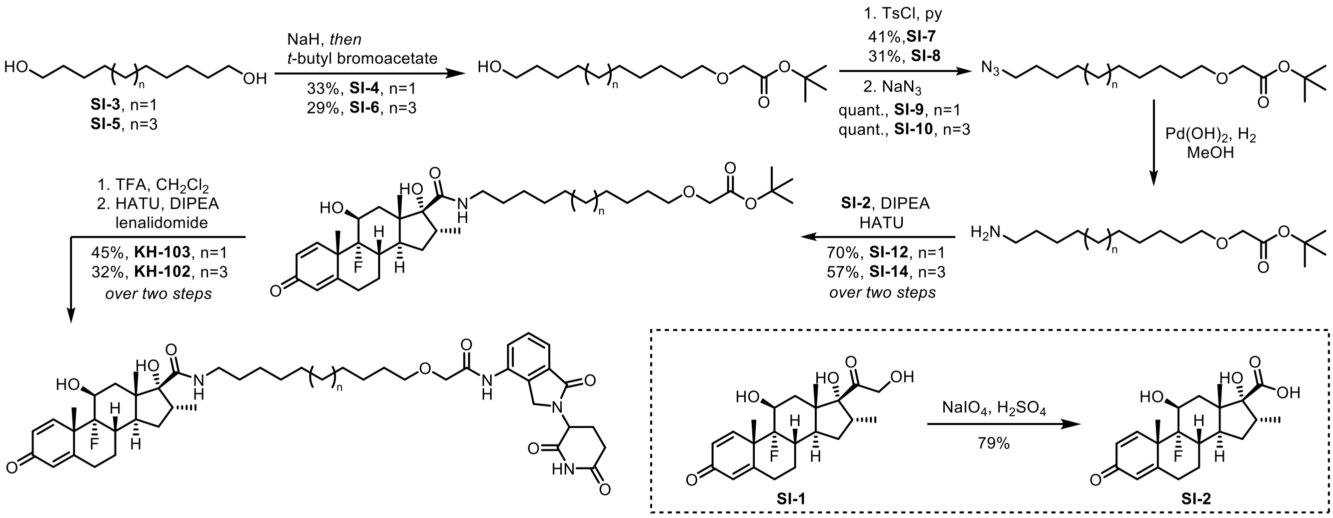

Supplementary Fig. 1. Synthesis of PROTAC KH-102 and PROTAC KH-103. Synthetic procedures and characterization of PROTAC KH-102 and PROTAC KH-103 are detailed in Supplementary Methods and all NMR spectra of the PROTACs and their synthetic intermediates are provided in Supplementary Data 1.

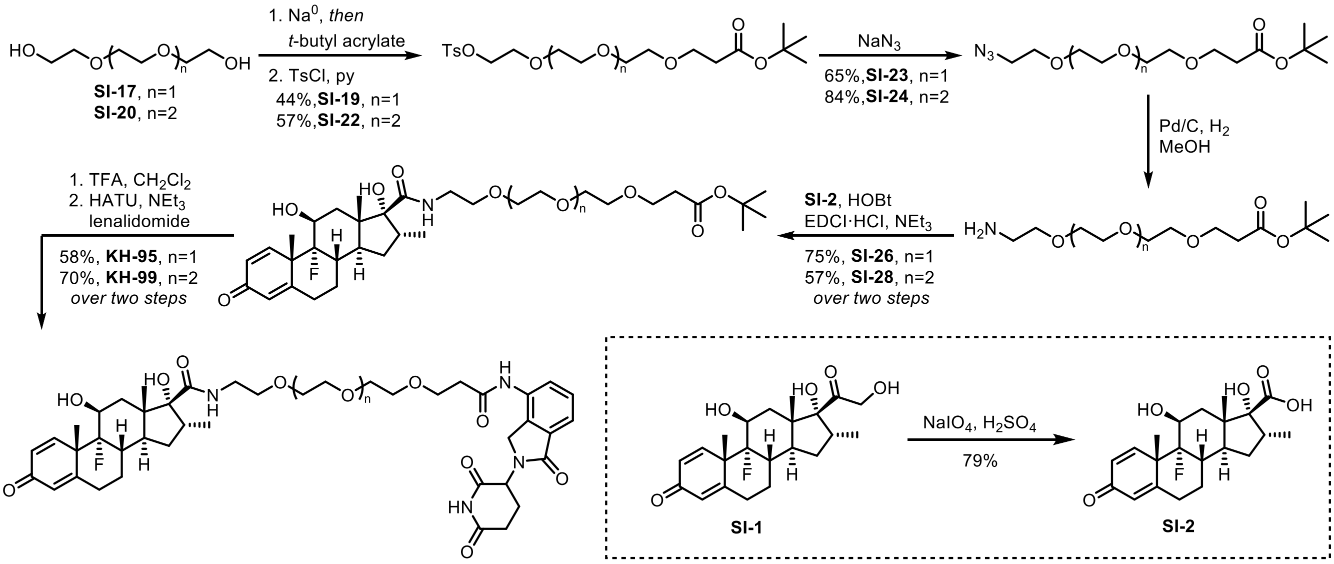

Supplementary Fig. 2. Synthesis Scheme of PROTAC KH-95 and PROTAC KH-99. Synthetic procedures and characterization of PROTAC KH-95 and PROTAC KH-99 are detailed in Supplementary Methods and all NMR spectra of the PROTACs and their synthetic intermediates are provided in Supplementary Data 1.

a

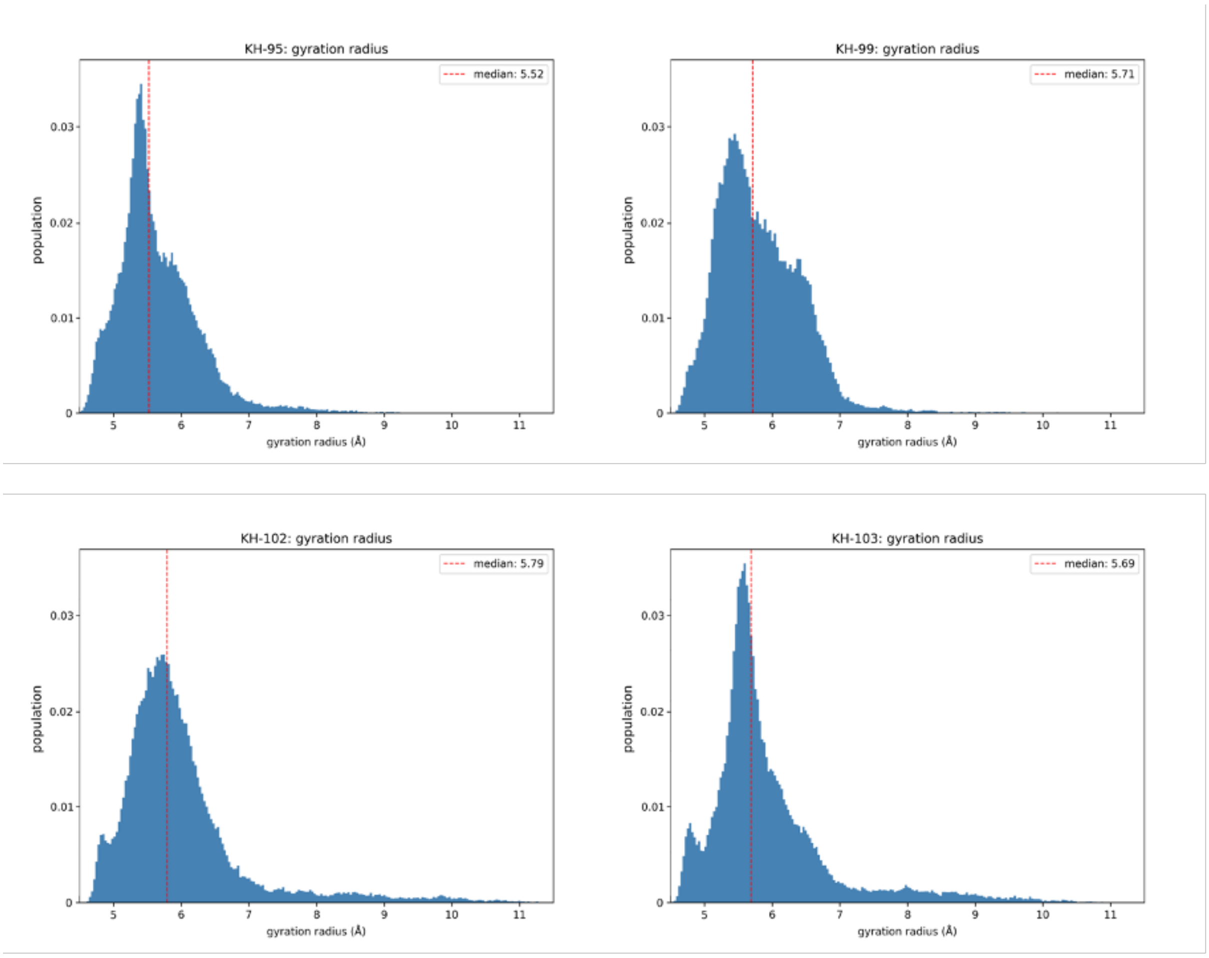
KH-95: gyration radius
median: 5.52
population
gyration radius (Å)
KH-99: gyration radius
median: 5.71
population
gyration radius (Å)
KH-102: gyration radius
median: 5.79
population
gyration radius (Å)
KH-103: gyration radius
median: 5.69
population
gyration radius (Å)

b

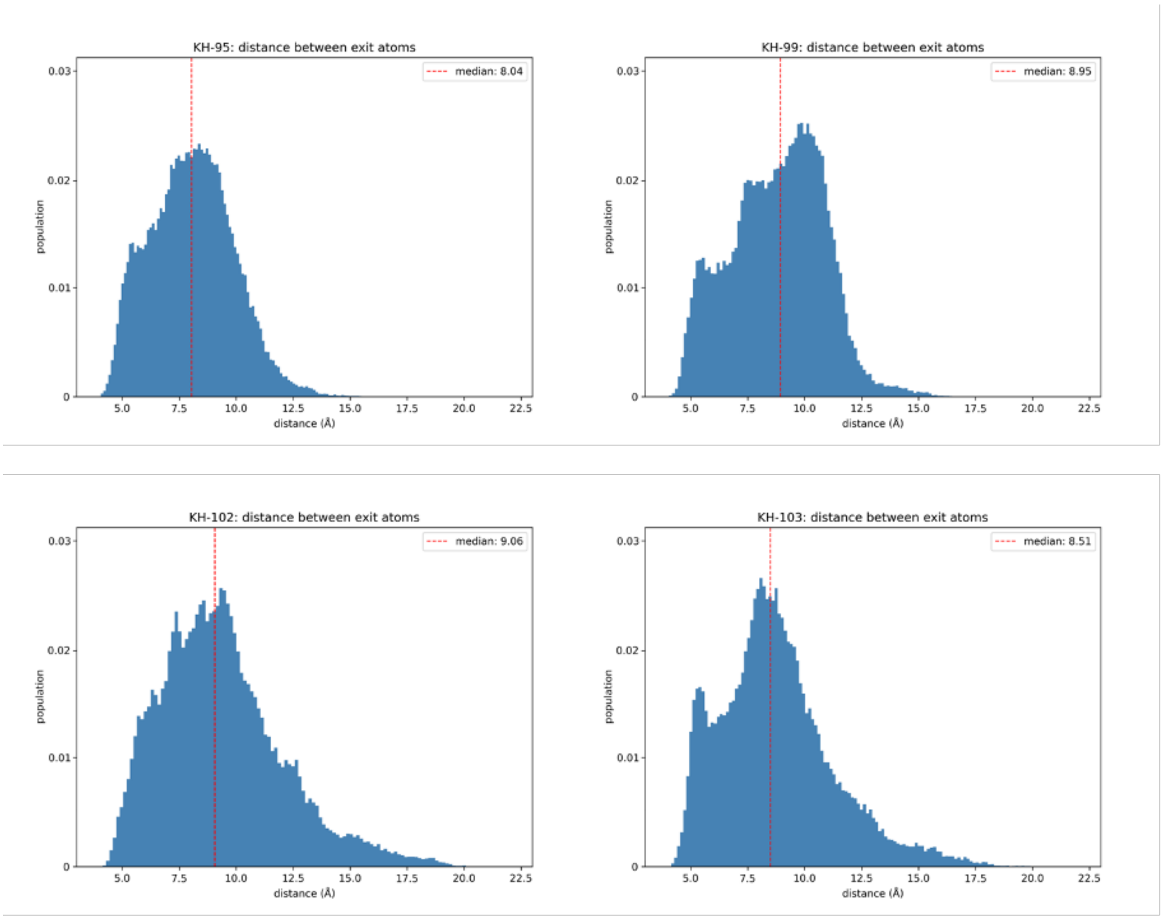
KH-95: distance between exit atoms
median: 8.04
population
distance (Å)
KH-99: distance between exit atoms
median: 8.95
population
distance (Å)
KH-102: distance between exit atoms
median: 9.06
population
distance (Å)
KH-103: distance between exit atoms
median: 8.51
population
distance (Å)

c

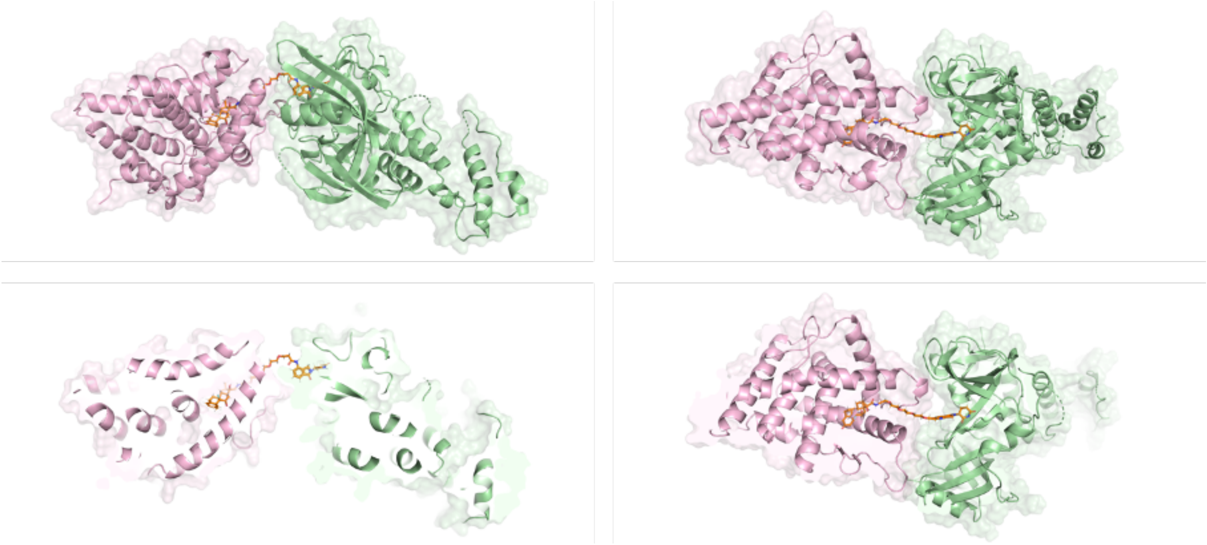

d

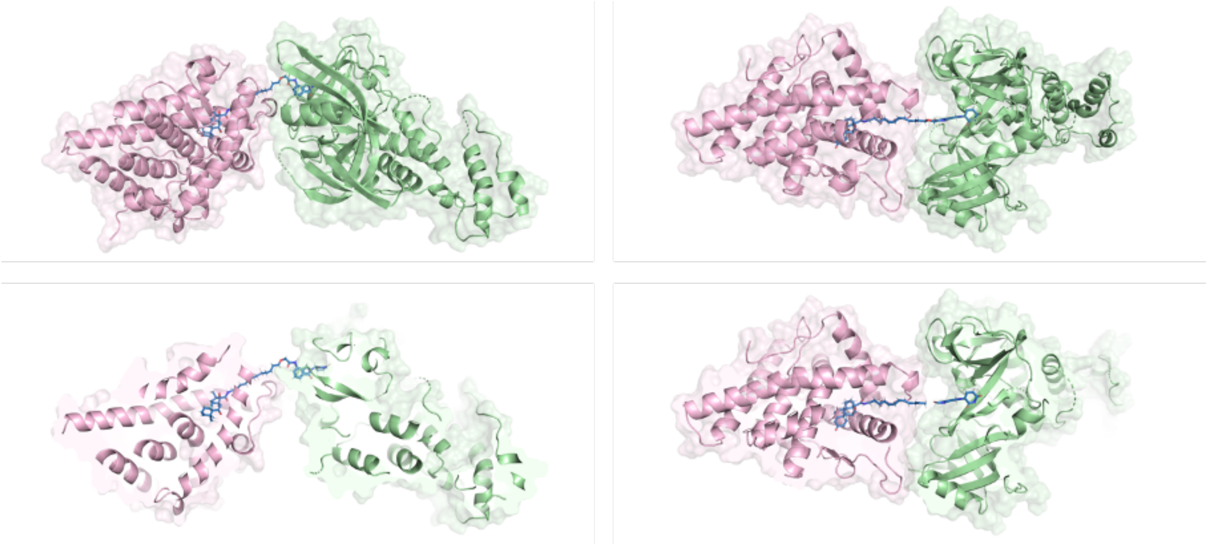

e

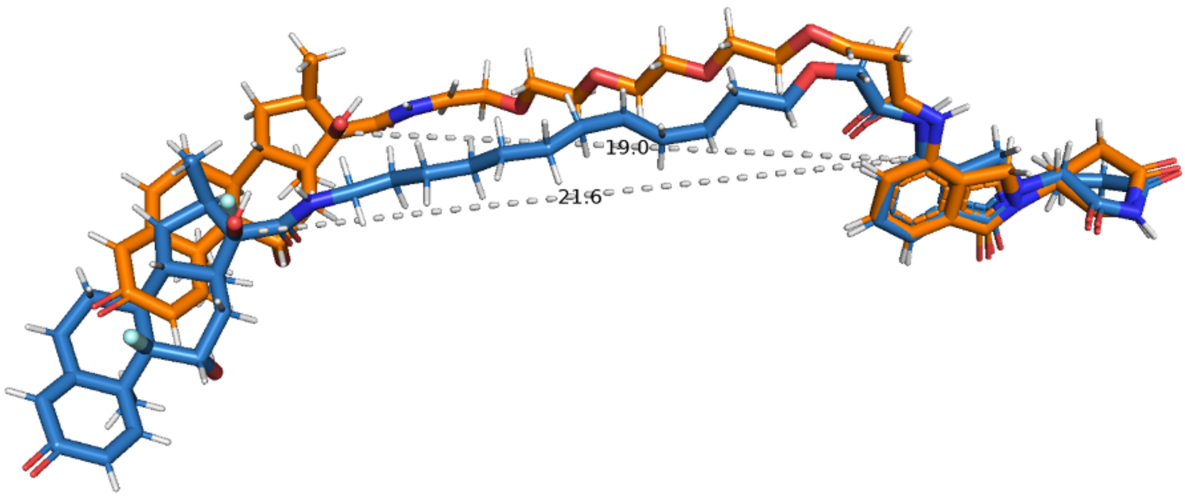

f

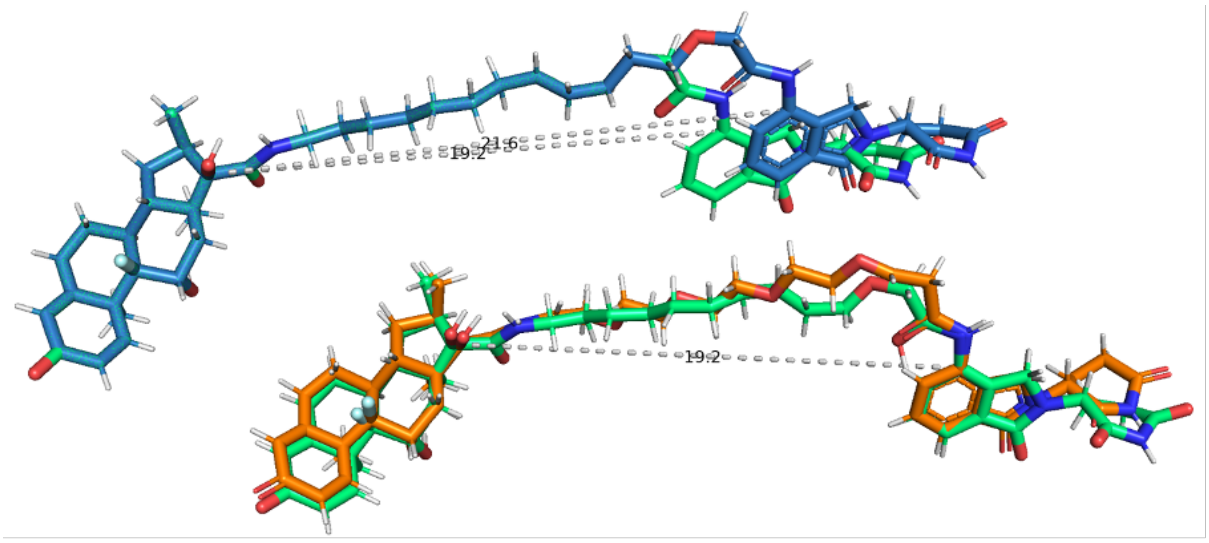

g

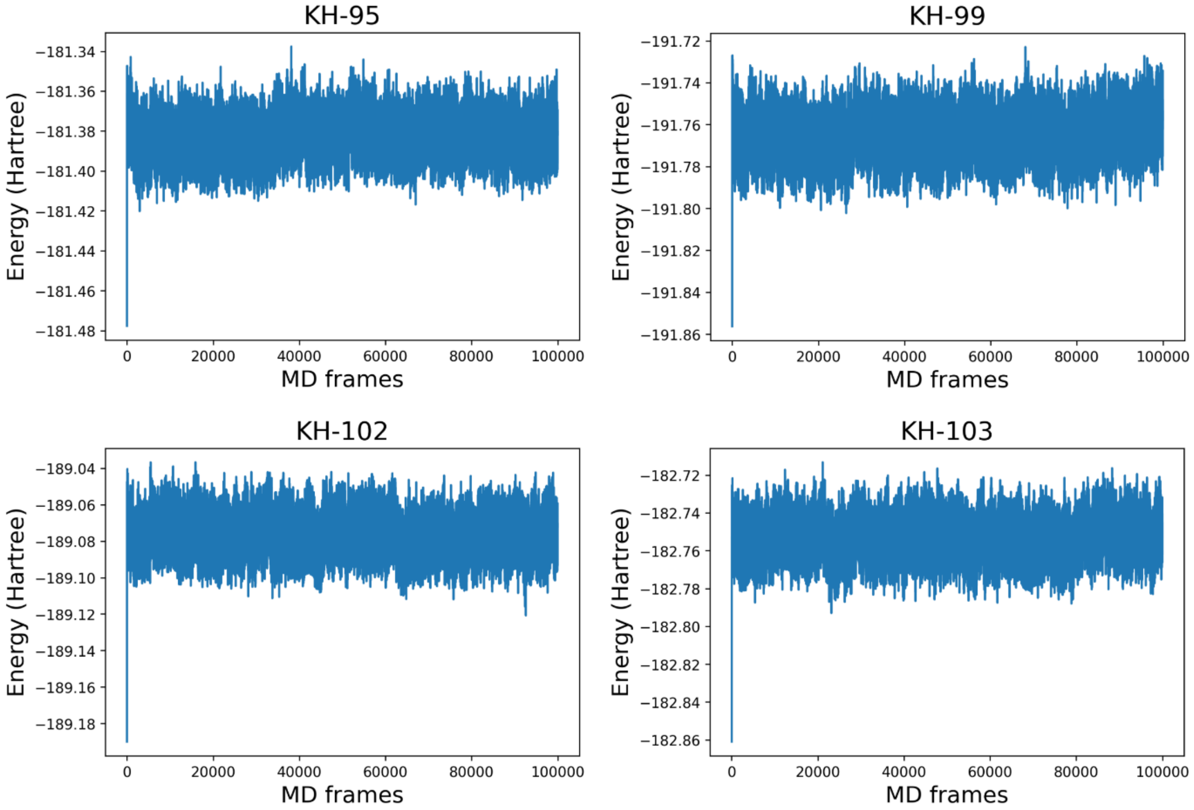

Supplementary Fig. 3 Molecular dynamics simulations and docking simulations via the PRosettaC web server. (a) Histograms of the gyration radii of the four PROTACs KH-95 (top, left), -99 (top, right), -102 (bottom, left) and -103 (bottom, right) from MD simulations. The red

dashed line marks the median value. (b) Histograms of the distances between the exit atoms of the four PROTACs KH-95 (top, left), -99 (top, right), -102 (bottom, left) and -103 (bottom, right) from MD simulations. The red dashed line marks the median value. (c) Structure of the ternary complex of GR – KH-99 – CRBN (light pink – orange – light green) from two different viewing angles (top) and the corresponding clip outs (bottom) as simulated by PRosettaC and reported as the highest score. (d) Structure of the ternary complex of GR – KH-102 – CRBN (light pink – blue – light green) from two different viewing angles (top) and the corresponding clip outs (bottom) as simulated by PRosettaC and reported as the highest score. (e) Structure of the PROTAC molecules KH-99 (orange) and -102 (blue), including the distance between the exit atoms, taken from the ternary structure as simulated by PRosettaC and reported as the highest score. (f) Structure of the PROTAC molecules KH-99 (orange) and -102 (blue) in comparison with KH-103 (green; modified from the KH-102 structure), including the distance between the exit atoms, taken from the ternary structure as simulated by PRosettaC and reported as the highest score. (g) Energy of the system as a function of MD trajectory frames for each PROTAC molecule.

a

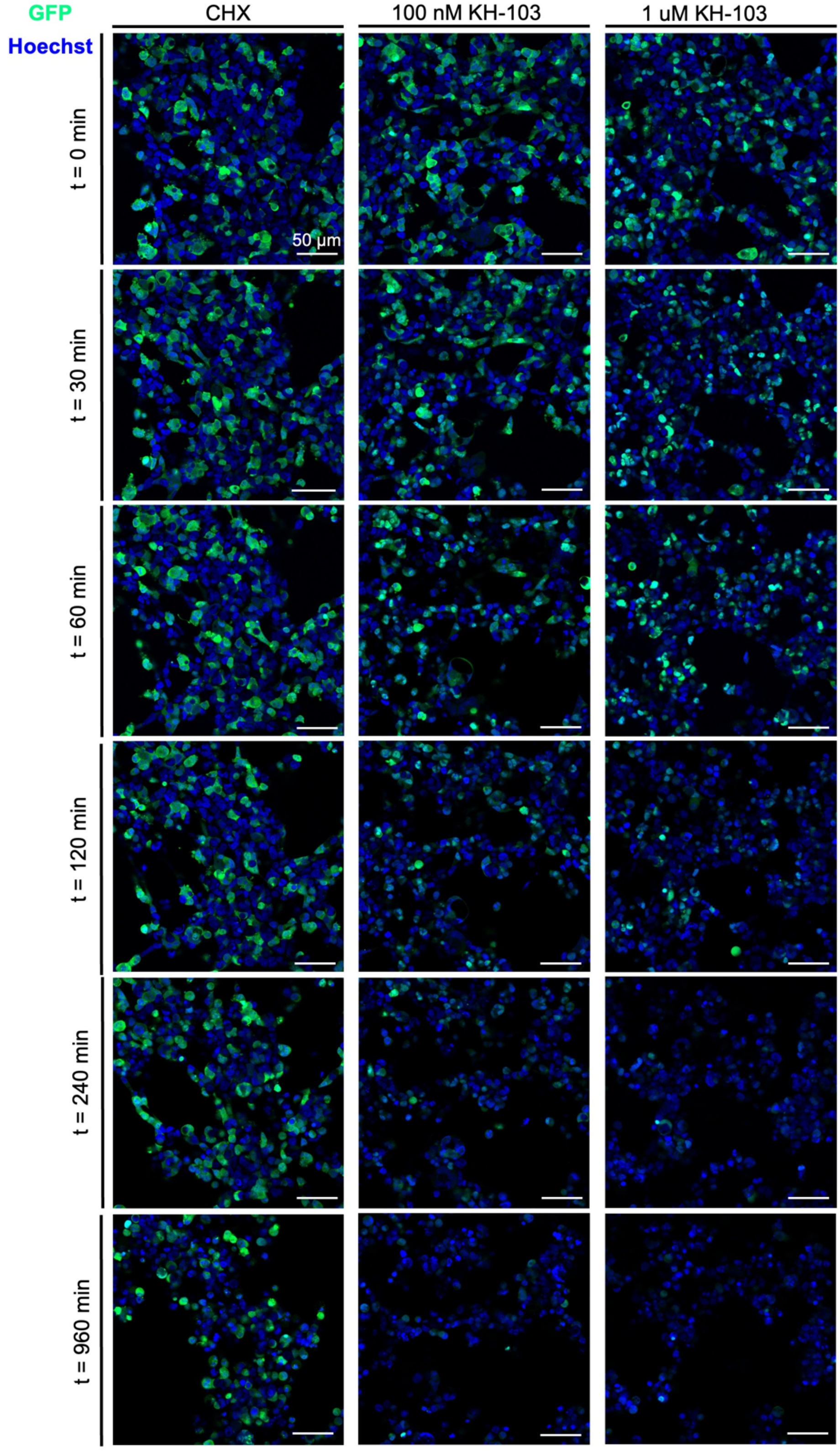
GFP
Hoechst
CHX
100 nM KH-103
1 uM KH-103
t = 0 min
t = 30 min
t = 60 min
t = 120 min
t = 240 min
t = 960 min
50 µm

b

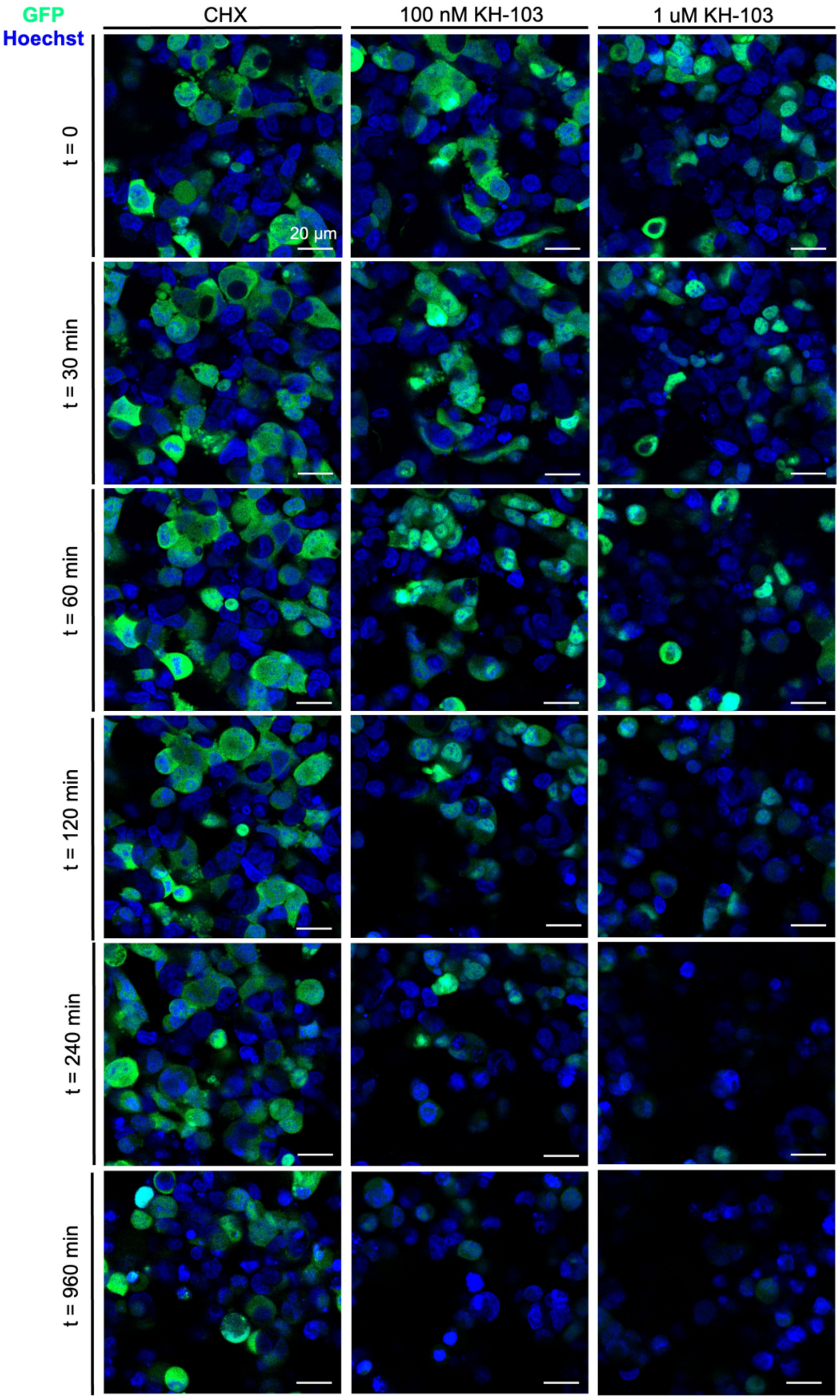

Supplementary Fig. 4 Live imaging of HEK293 cells transiently expressing eGFP-GR and exposed to KH-103 or DMSO in the presence of cycloheximide (CHX) (a protein synthesis inhibitor) (n=1/condition) across time (in minutes). Scale bars represent 50µm (a) and 20µm (b) as depicted.

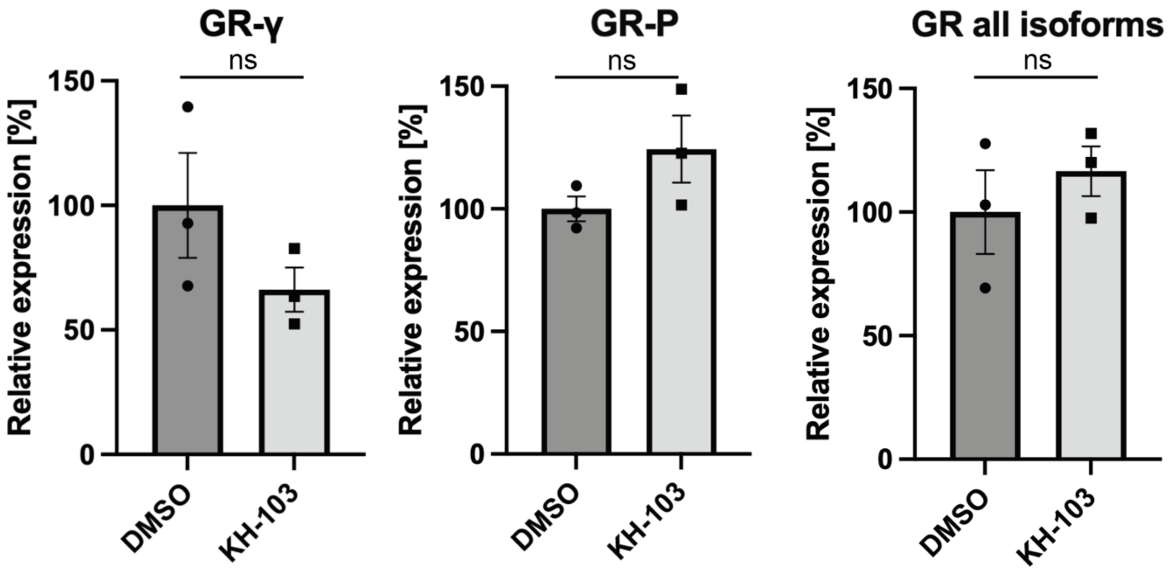

Supplementary Fig. 5 Q-RT-PCR results depicting the expression of GR transcriptional isoforms gamma, P and alpha in HEK cells following 16 hours of KH-103 treatment GR-y: DMSO n=3, KH-103 n=3; GR-P: DMSO n=3, KH-103 n=3; GR all isoforms: DMSO n=3, KH-103 n=3. Data are presented as mean values +/- SEM.

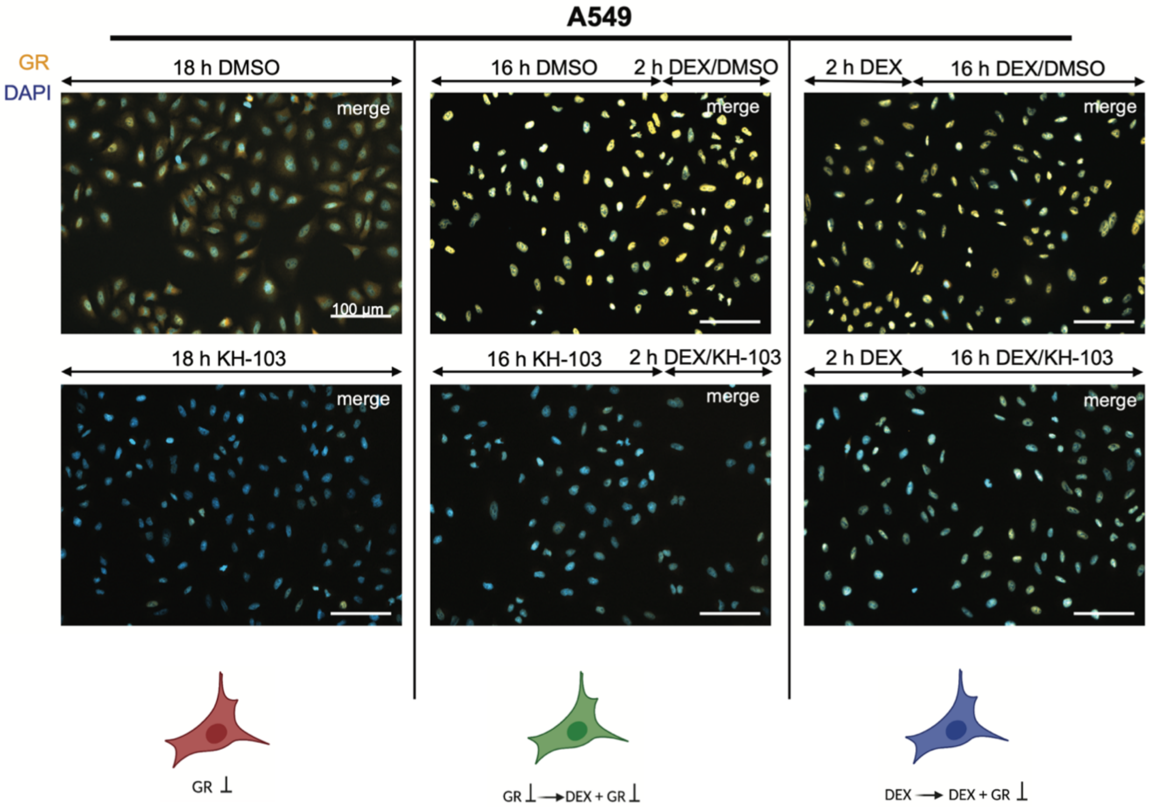

Supplementary Fig. 6 Immunofluorescent staining of GR in A549 cells treated according to the RNAseq experiment conditions described in Fig. 6a. Scale bars: 100 µm

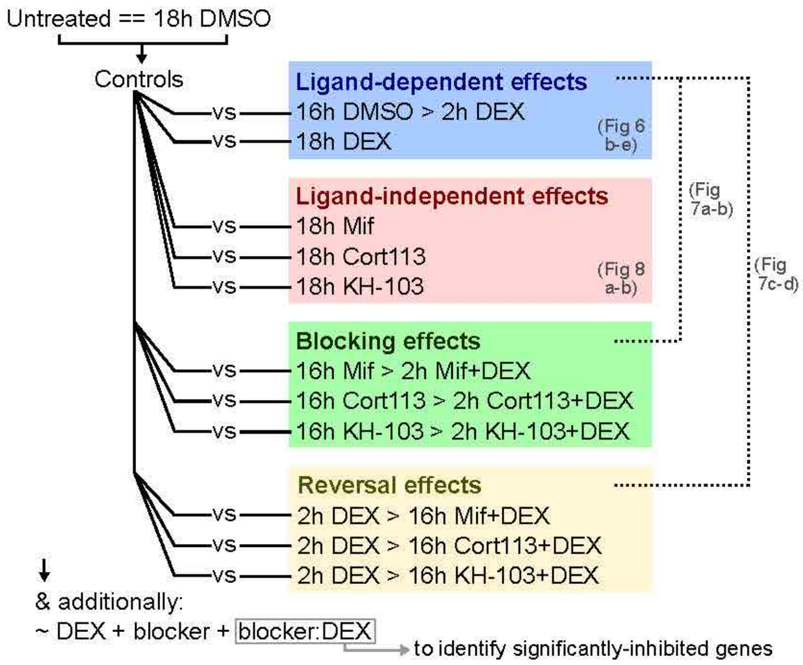

Supplementary Fig. 7 Scheme depicting the bioinformatic quantification workflow of RNA sequencing in A549 cells. Initially, since no change between untreated and DMSO condition was observable, these two conditions were merged. Subsequently they were subjected a) to pairwise comparisons with either DEX only group (Fig.7a-b), or ligand only group (Fig8 a-b). Additionally, significant interactions were determined between untreated, DEX and blocker + DEX groups to identify significantly-inhibited genes using the model with the tested coefficient.

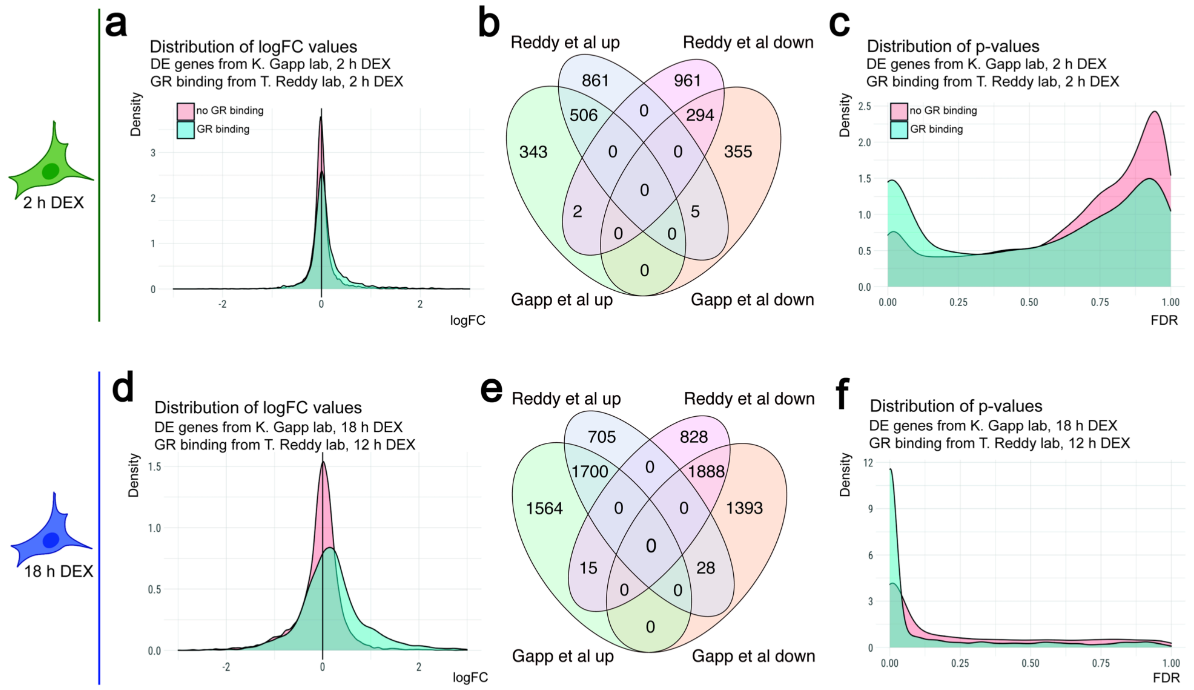

Supplementary Fig. 8 DEGs in 2 h (a &b) and 18 h DEX treatment (d-e) with a GR binding signal within + / - 2500 bp from their promoters, showed higher fold changes (shift of the curve to the right or left, away from zero) than those without GR binding signal. This was particularly pronounced at the 18 h time point (d). DEGs in 2 h (c) and 18 h DEX treatment (f) with a GR binding signal within + / - 2500 bp from their promoters, showed higher density of smaller p-values than those without a GR peak.

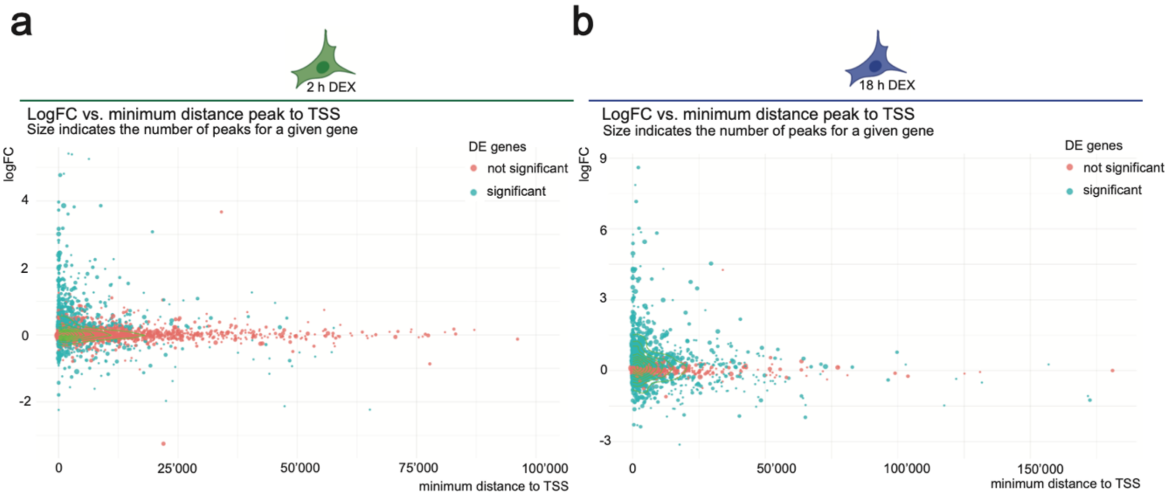

Supplementary Fig. 9 For DEGs in both 2 h (a) and 18 h (b) DEX treatment, the further away the GR binding signal peak from the gene's TSS, the smaller logFC was observed.

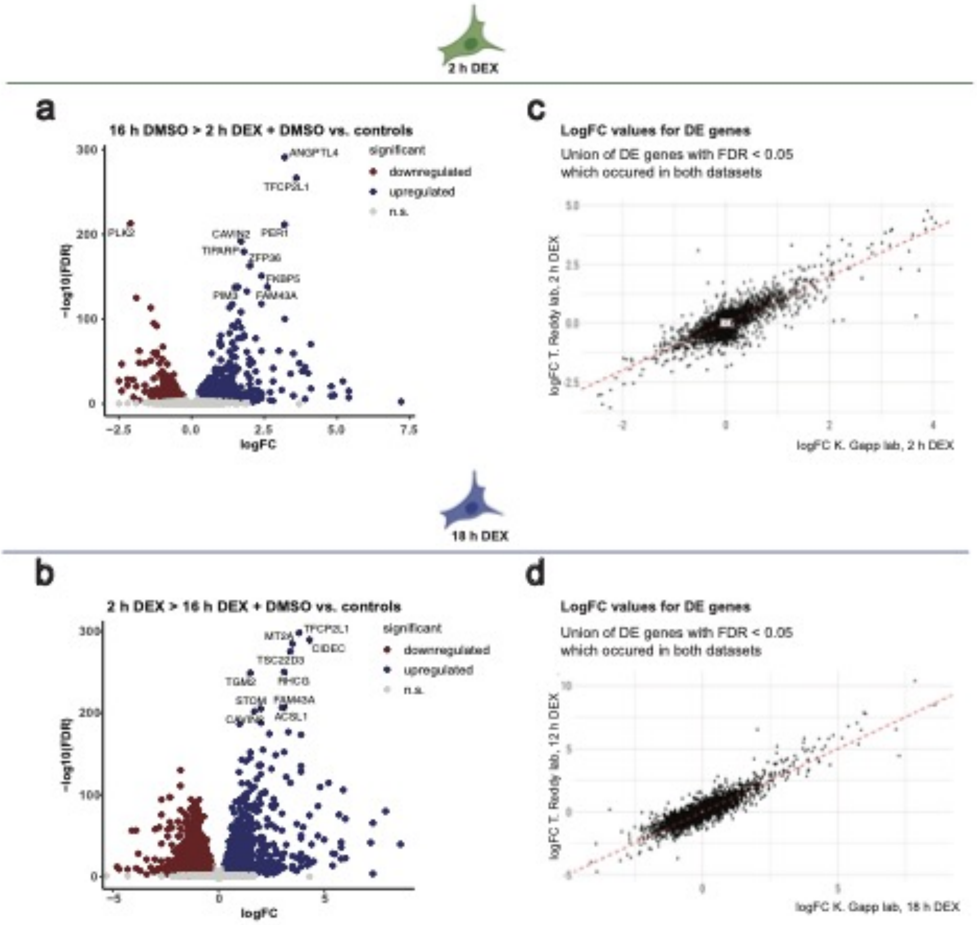

Supplementary Fig. 10 Volcano plots depicting differentially expressed genes following (a) 2 hours and (b) 18 hours of DEX treatment in A549 cells. Correlations of logFC between Gapp lab and Reddy lab for (c) 2 hours DEX treatment and (d) 18 hours respectively 12 hours DEX treatment.

a

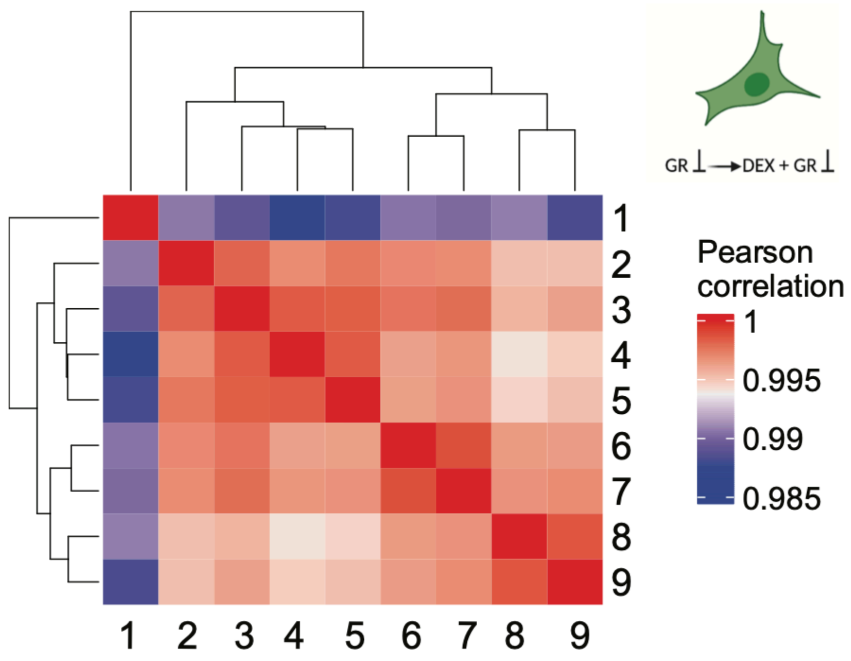

b

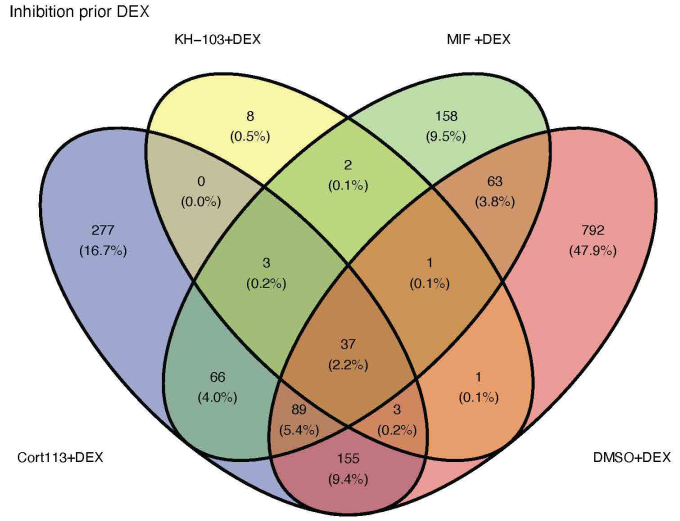

Supplementary Fig. 11 (a) Pearson correlations and clustering of the conditions based on log(CPM) of the union of DEGs in DEX 2 h condition. 1 : 16 h DMSO > 2 h DEX + DMSO, 2 : 16 h KH-103 > 2 h DEX + KH-103, 3 : 18 h KH-103, 4 : 18 h untreated, 5 : 18 h DMSO, 6 : 16 h MIF > 2 h DEX + MIF, 7 : 18 h MIF, 8 : 16 h CORT113176 > 2 h DEX + CORT113176, 9 : 18 h CORT113176. CPM : Counts Per Million. (b) Venn diagram depicting the overlaps of efficient blockage of DEX-induced changes between MIF, CORT113 and KH-103 treatment.

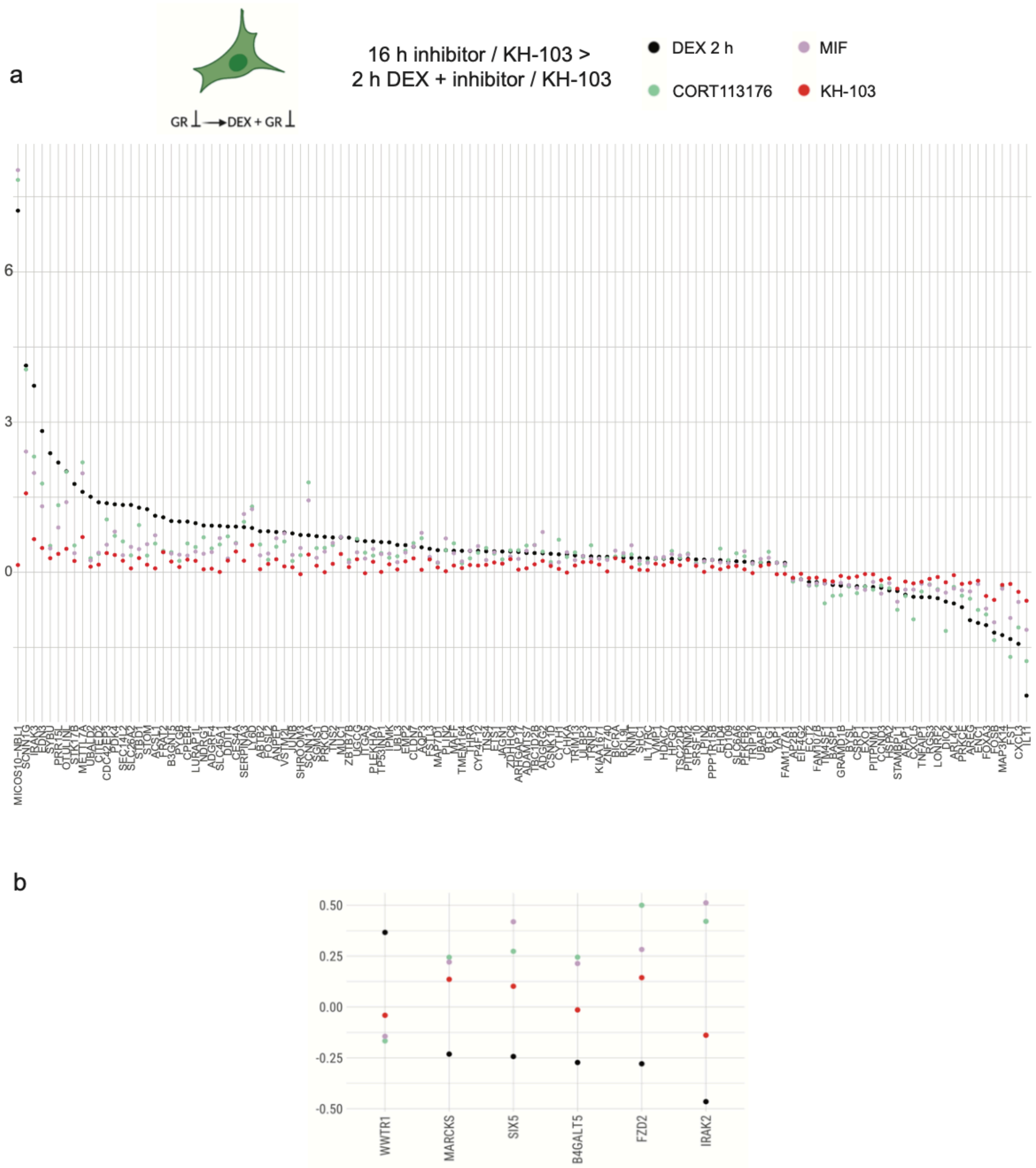

Supplementary Fig. 12 (a) Genes in the blocking group that showed no significant change upon pretreatment with KH-103 and remained comparable to controls, but that were significantly changed in the same direction as DEX when pretreated with inhibitors (MIF or CORT113176) prior to DEX exposure. (b) Genes that showed no significant change upon pretreatment with KH-103 and remained comparable to controls but were significantly changed upon the inhibitors to the opposite direction as DEX.

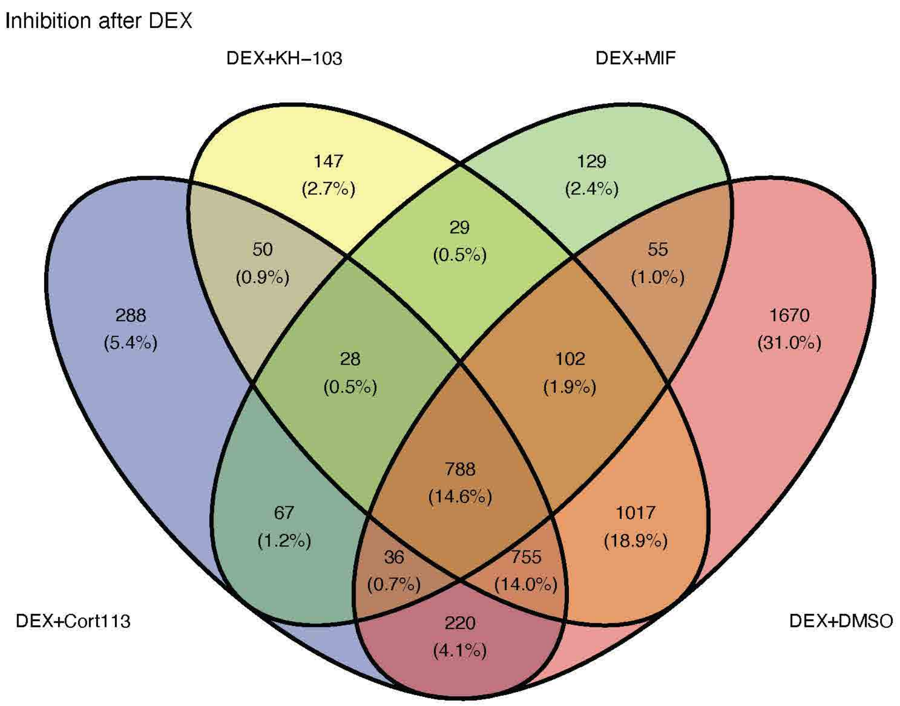

Supplementary Figure 13: Venn diagram depicting the overlaps of successful inhibition by either KH-103, MIF or CORT113176 of DEX-induced DEGs.

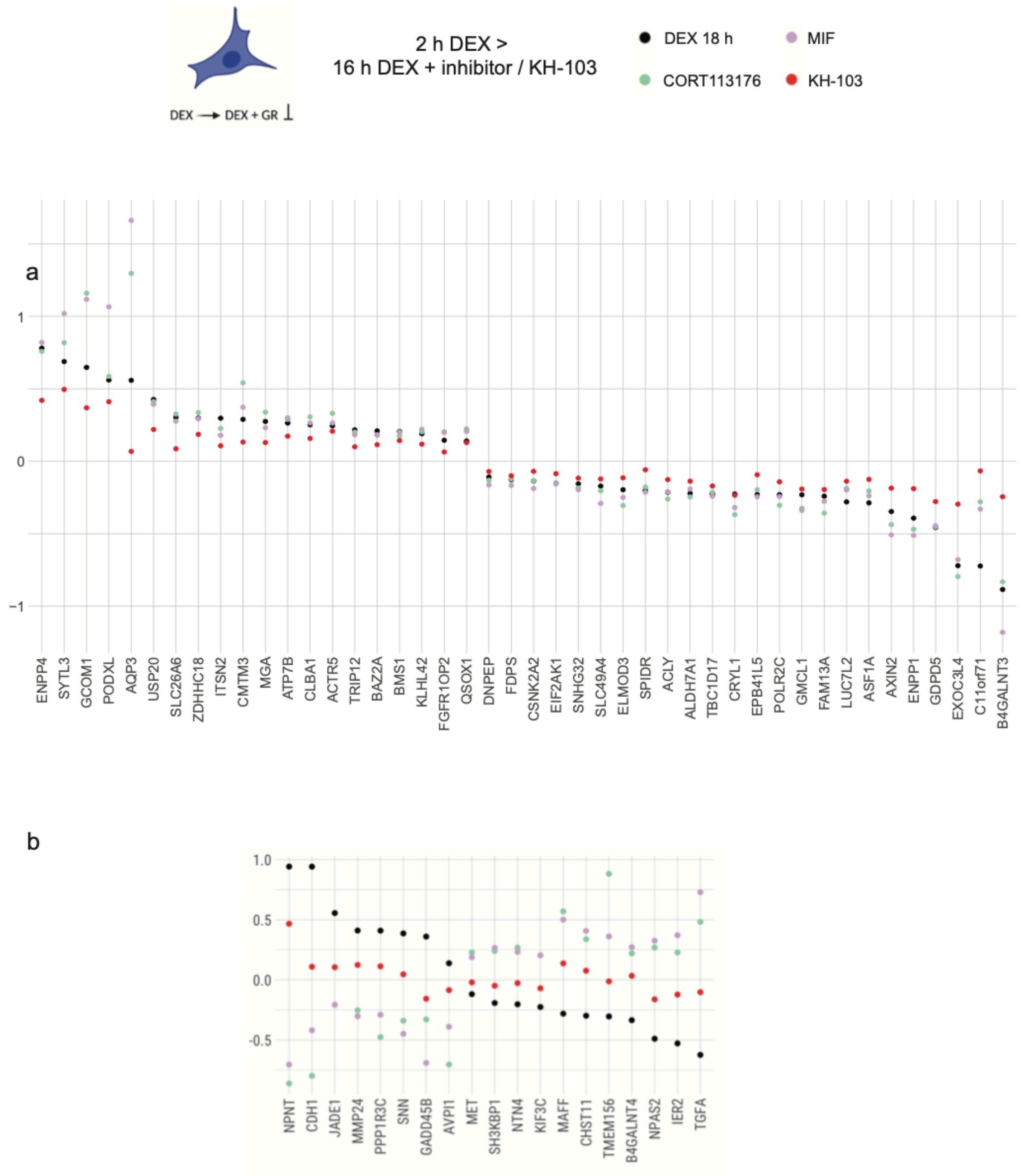

Supplementary Fig. 14 (a) Genes in the reversing group which were significantly changed in the same direction as DEX by the inhibitors, but addition of KH-103 reversed them to comparable levels as controls (were not significantly changed). (b) Genes that showed no significant change compared to controls upon reversing with KH-103 but were significantly changed by the inhibitors in the opposite direction as DEX.

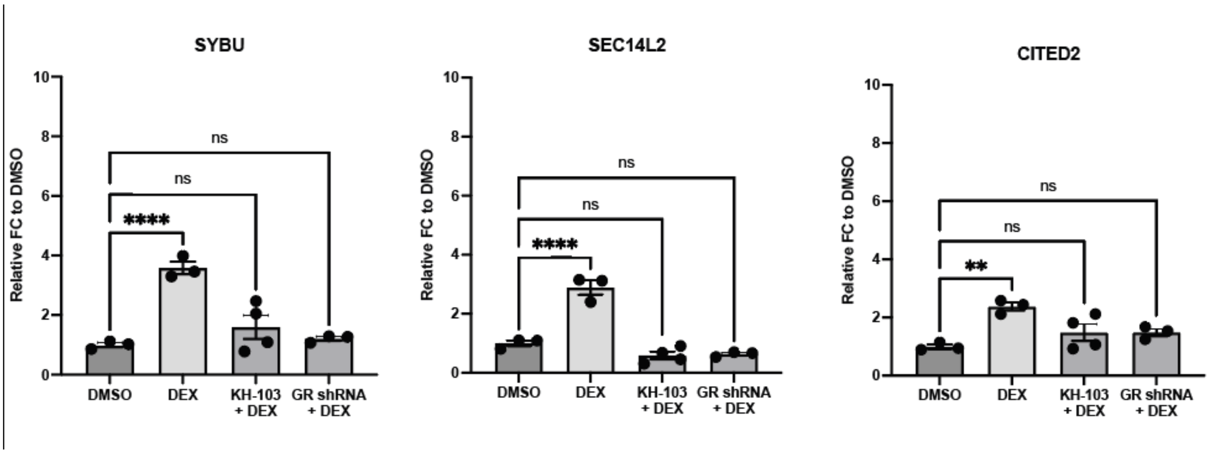

Supplementary Figure 15 Effects of shRNA mediated knockdown of GR versus KH-103 mediated depletion on gene transcripts triggered by DEX but not blocked by MIF or CORT113176 (from the gene list shown in supplementary Fig. 8a). Results were normalised to HPRT and PPIA housekeeping genes. All groups $n = 3$, with the exception for KH-103 + DEX group $n = 4$. For SYBU, Ordinary one-way ANOVA showed a significant difference ($F(4, 11) = 19.04$, $p = < 0.0001$). Follow-up Dunnett's multiple comparisons showed significant differences for DMSO vs. DEX ($p = < 0.0001$). For SEC14L2, Ordinary one-way ANOVA showed a significant difference ($F(4, 11) = 44.53$, $p = < 0.0001$). Follow-up Dunnett's multiple comparisons showed significant differences for DMSO vs. DEX ($p = < 0.0001$). For CITED2, Ordinary one-way ANOVA showed a significant difference ($F(4, 11) = 9.656$, $p = 0.0013$). Follow-up Dunnett's multiple comparisons showed significant differences for DMSO vs. DEX ($p = 0.0014$). P-values ** < 0.01, **** < 0.0001. Data are presented as mean values +/- SEM. Source data are provided as a Source Data file.

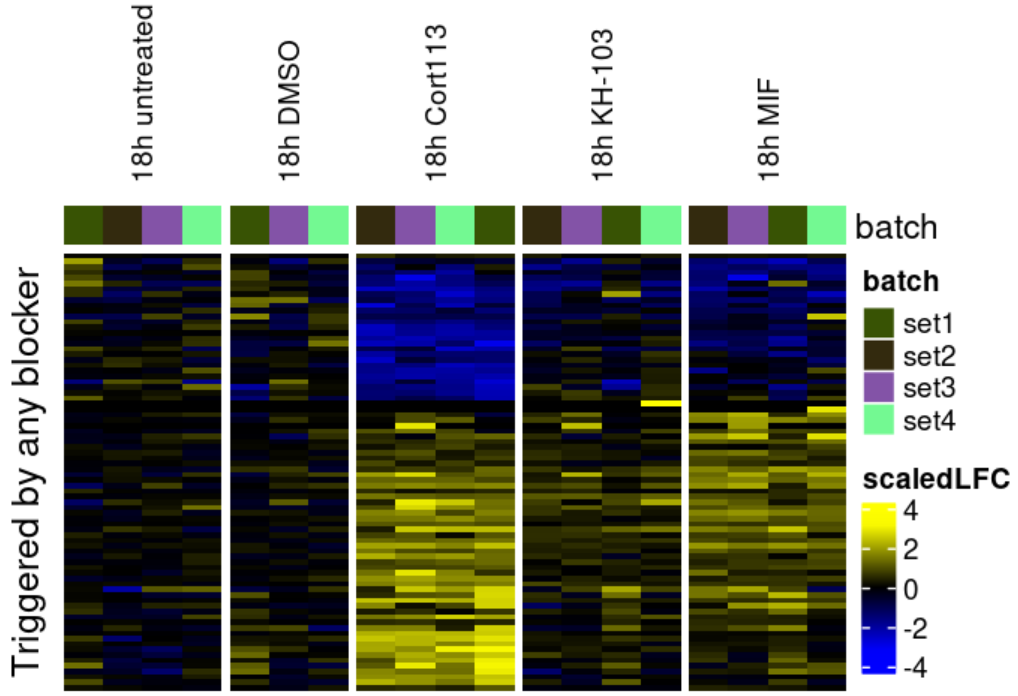

Supplementary Figure 16 Heatmap showing changes induced by ligand independent treatment with KH-103, Mif and CORT113 applying a more stringent fold change threshold (|*logFC*|>*1*) persistently demonstrates less changes triggered by KH-103 as opposed to other inhibitors.

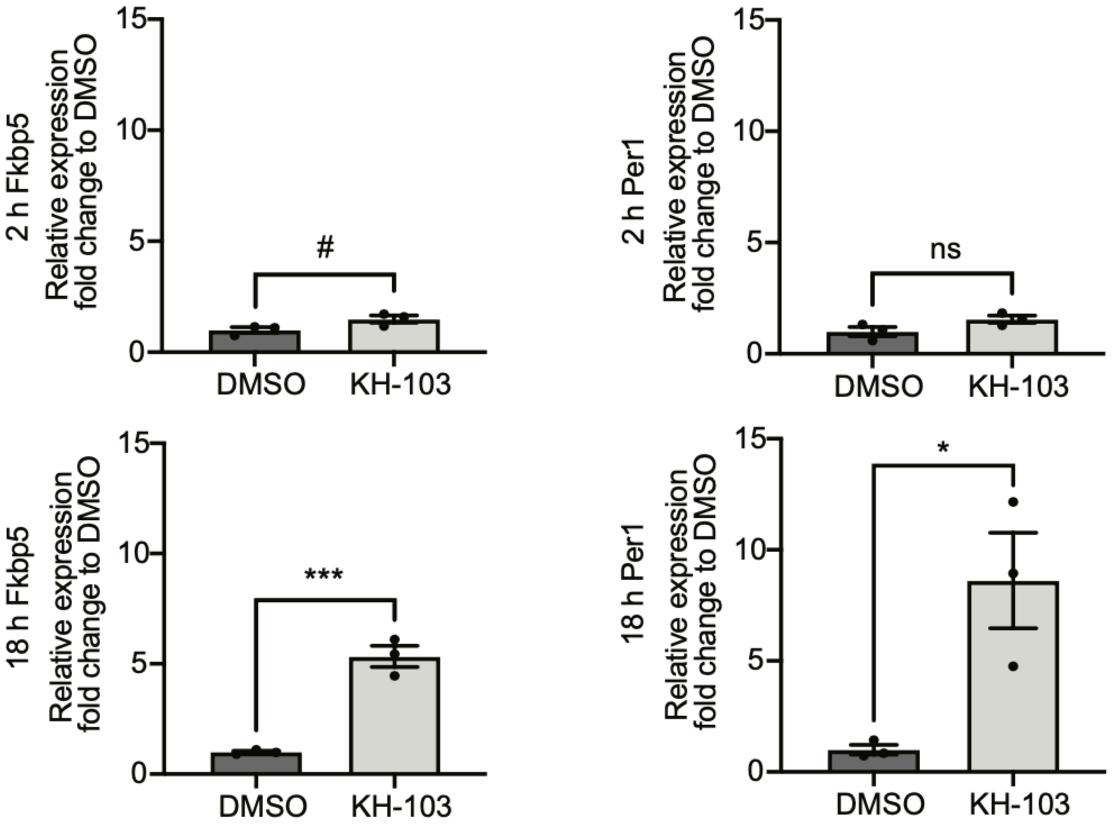

Supplementary Fig. 17 Validation of FKBP5 and Per1 mRNA expression changes upon KH-103 at 2 h and 18 h in A549 cells via qRT-PCR. Results were normalised to HPRT and PPIA housekeeping genes. N = 3. For FKBP5, unpaired, two-tailed t-test showed a trend between DMSO and KH-103 at 2 h ($t(4) = 2.42$, $p = 0.0729$), and a significant difference at 18 h ($t(4) = 8.92$, $p = 0.0009$). For Per1, unpaired, two-tailed t-test showed no significant difference between DMSO and KH-103 at 2 h ($t(4) = 2.10$, $p = 0.1032$), and a significant difference at 18 h ($t(4) = 3.53$, $p = 0.0242$). P-values # < 0.1, * < 0.05, *** < 0.001. Data are presented as mean values +/- SEM. Source data are provided as a Source Data file.

a

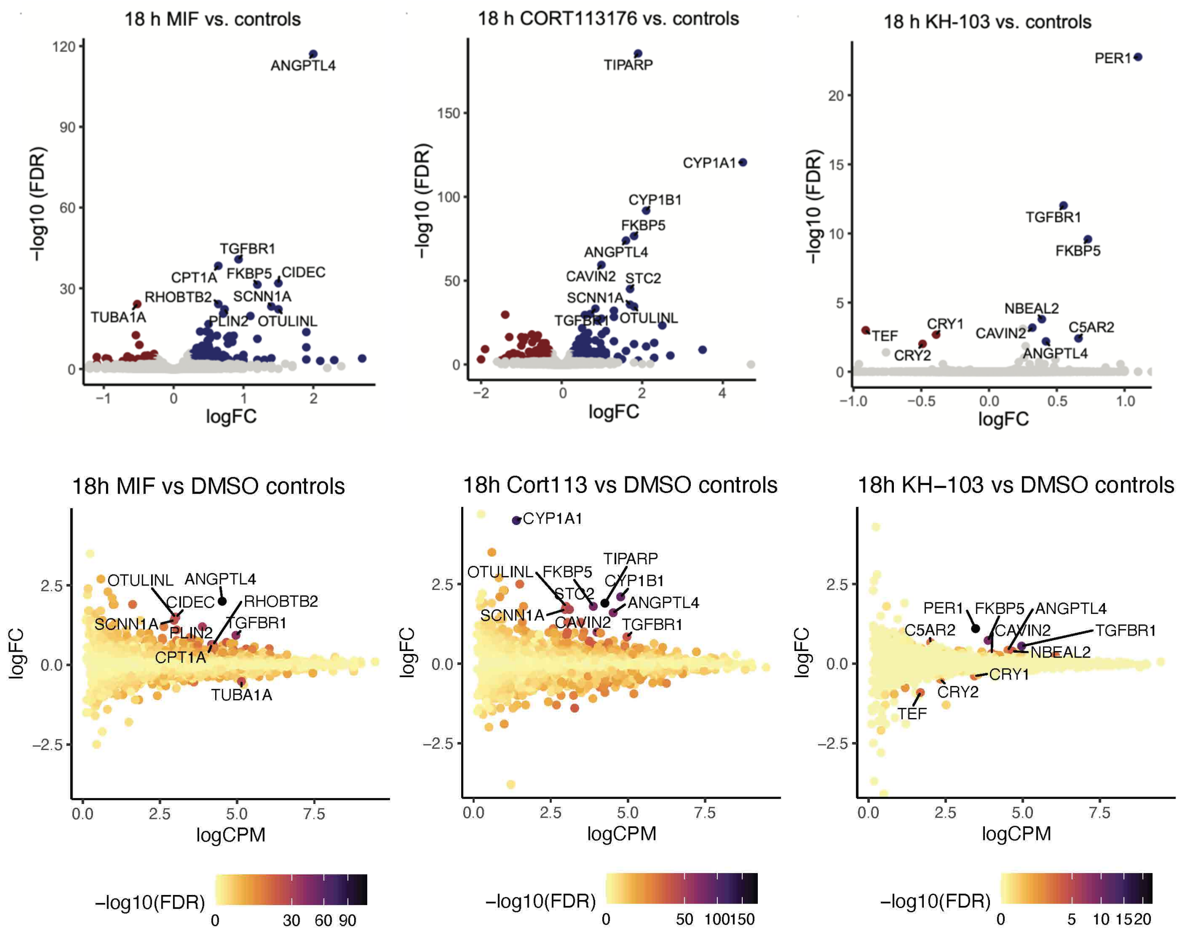
18 h MIF vs. controls
18 h CORT113176 vs. controls
18 h KH-103 vs. controls
−log10 (FDR)
logFC
ANGPTL4
TGFBR1
CPT1A
FKBP5
CIDEC
RHOBTB2
SCNN1A
TUBA1A
PLIN2
OTULINL
TIPARP
CYP1A1
CYP1B1
CAVIN2
STC2
PER1
NBEAL2
CRY1
C5AR2
TEF
CRY2
18h MIF vs DMSO controls
18h Cort113 vs DMSO controls
18h KH−103 vs DMSO controls
logFC
logCPM
−log10(FDR)

b

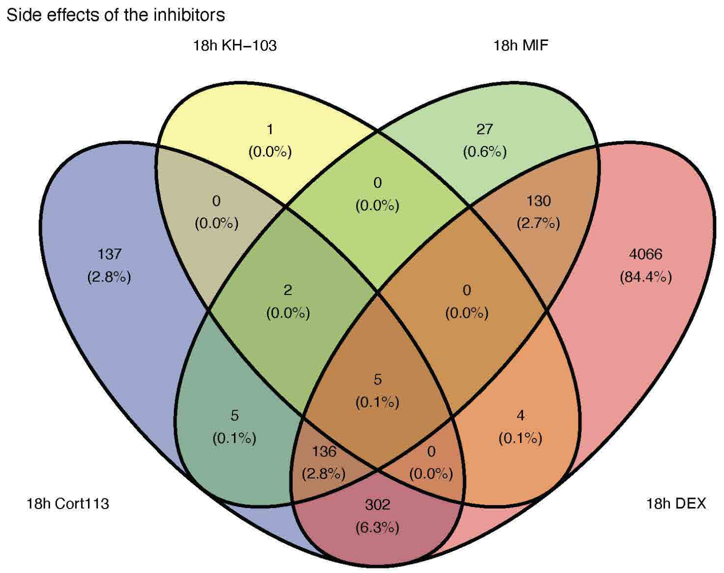
Side effects of the inhibitors
18h KH−103
18h MIF
18h Cort113
18h DEX
1 (0.0%)
27 (0.6%)
0 (0.0%)
0 (0.0%)
130 (2.7%)
137 (2.8%)
4066 (84.4%)
2 (0.0%)
0 (0.0%)
5 (0.1%)
5 (0.1%)
4 (0.1%)
136 (2.8%)
0 (0.0%)
302 (6.3%)

Supplementary Fig. 18 (a) Volcano plots and MA plots of DEGs in A549 cells treated with MIF, CORT113176 , and KH-103 for 18 h normalized to controls (untreated and DMSO conditions). (b) Venn diagram depicting the overlaps of side effects of MIF, CORT113 and KH-103.

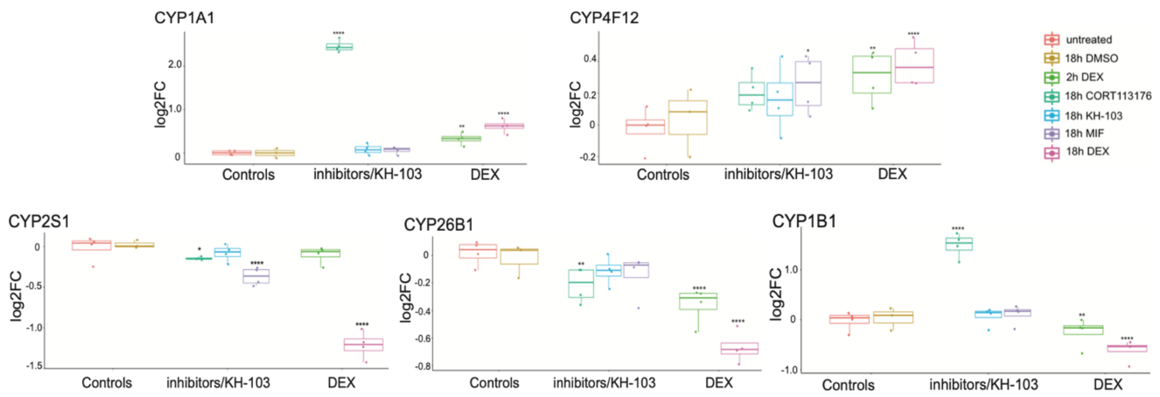

Supplementary Fig. 19 Comparison of CYPs genes expression changes obtained by RNAseq upon 18 h treatment with DEX, or MIF, or CORT113176, or KH-103. Stars represent FDR values # < 0.1, * < 0.05, ** < 0.01, **** < 0.0001, all the logFC and FDR values are summarised in Supplementary Table. 5). Boxplot minimum represents the 25% quantile and maxium the 75% quantile, the center line is the median, whiskers are all values that lie outside 1.5x interquartile range.

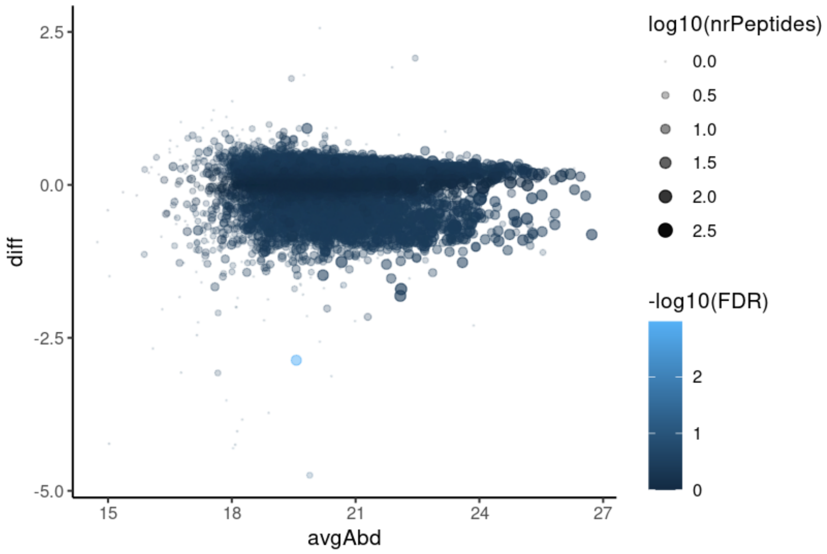

Supplementary Fig.20 MA plot depicting differential abundance of proteins (x-axis), the number of peptides they are represented with (dot size), their FDR significance values (blue color scale) and their fold change (y-axis) in A549 cells with or without KH-103 treatment for 16 h as assessed in a proteomic screen.

a

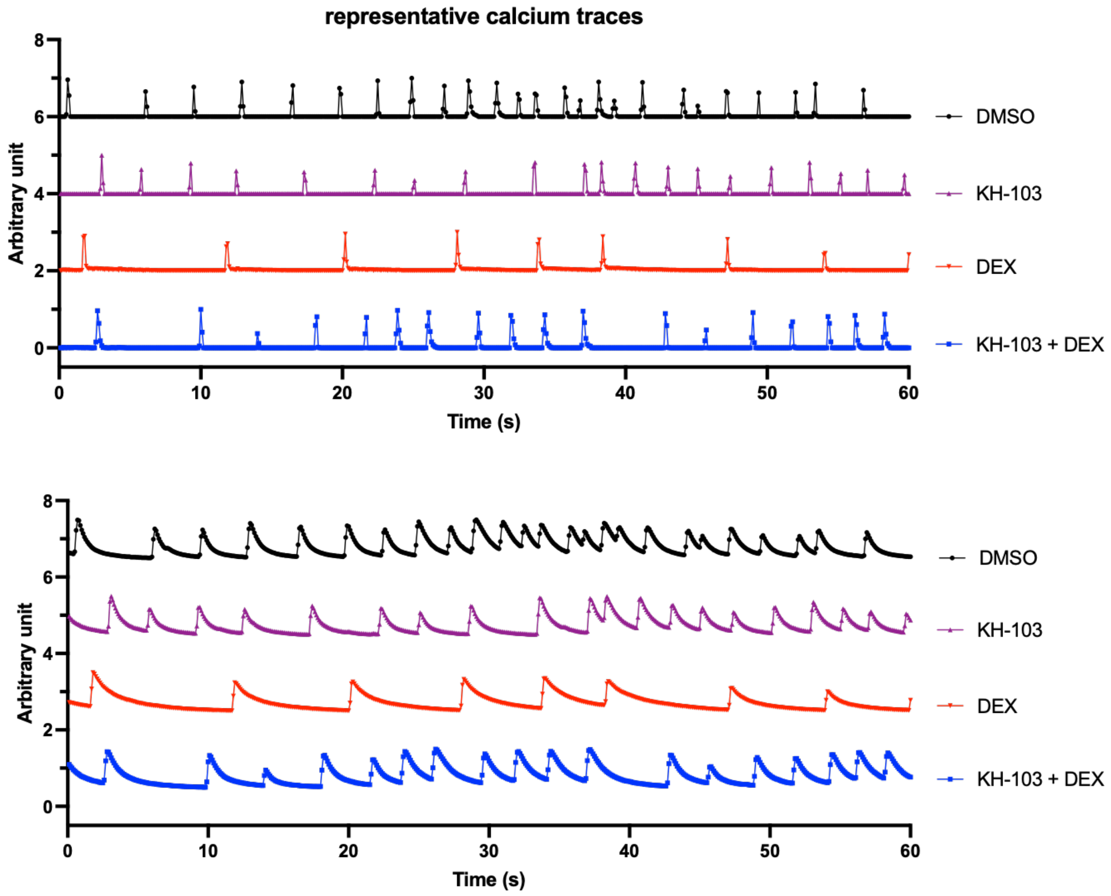
representative calcium traces
Arbitrary unit
Time (s)
DMSO
KH-103
DEX
KH-103 + DEX
Arbitrary unit
Time (s)
DMSO
KH-103
DEX
KH-103 + DEX

b

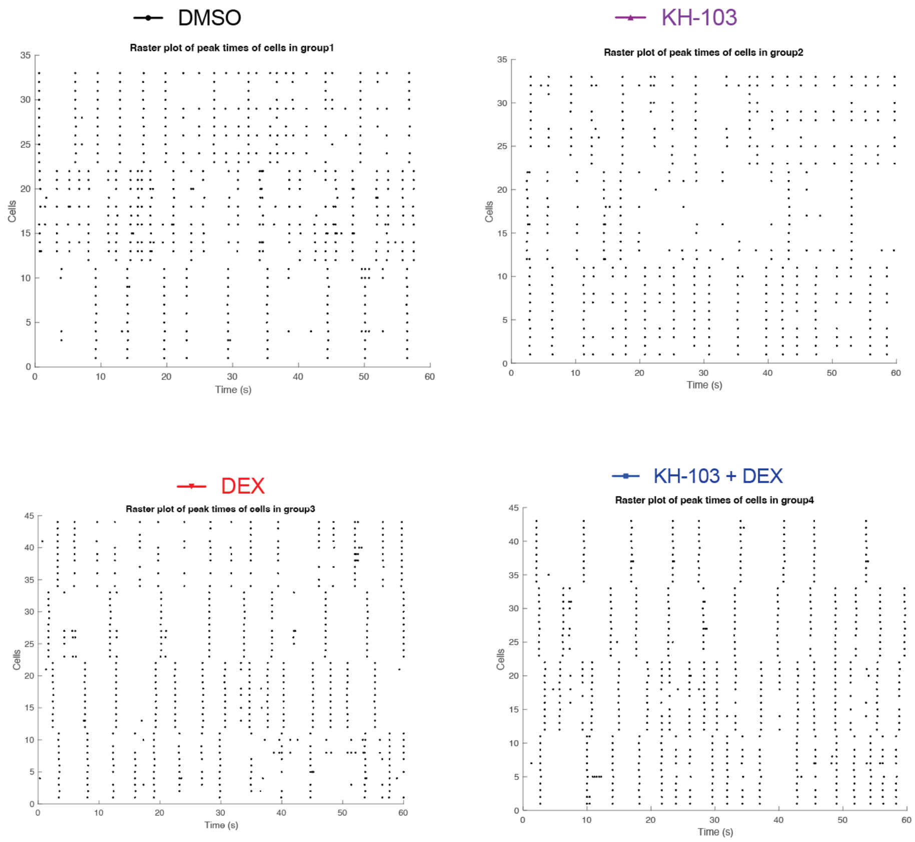

Supplementary Figure 21_(a) Representative traces of each group. (b) Raster plots of different conditions that depict peaks over time of (x-axis) each single neuron (y-axis) in one row.

Supplementary Table. 1-5_Gazorpak et al.

Fisher's LSD	Mean difference	95% CI of difference	Summary	P-Value
A: DMSO/DMSO vs. C: DEX/DEX	53.67	17.87 to 89.46	**	0.0067
A: DMSO/DMSO vs. E: DEX/DMSO	20.67	-15.13 to 56.46	ns	0.2323
A: DMSO/DMSO vs. B: DMSO/KH-103	96.33	60.54 to 132.10	****	< 0.0001
A: DMSO/DMSO vs. D: DEX/DEX+KH-103	62.67	26.87 to 98.46	**	0.0025
A: DMSO/DMSO vs. F: DEX/KH-103	92.67	56.87 to 128.50	***	0.0001
C: DEX/DEX vs. E: DEX/DMSO	-33.00	-68.79 to 2.79	#	0.0676
C: DEX/DEX vs. B: DMSO/KH-103	42.67	6.88 to 78.46	*	0.0233
C: DEX/DEX vs. D: DEX/DEX+KH-103	9.00	-26.79 to 44.79	ns	0.5938
C: DEX/DEX vs. F: DEX/KH-103	39.00	3.21 to 74.79	*	0.0351
E: DEX/DMSO vs. B: DMSO/KH-103	75.67	39.87 to 111.50	***	0.0006
E: DEX/DMSO vs. D: DEX/DEX+KH-103	42.00	6.21 to 77.79	*	0.0252
E: DEX/DMSO vs. F: DEX/KH-103	72.00	36.21 to 107.80	***	0.0009
B: DMSO/KH-103 vs. D: DEX/DEX+KH-103	-33.67	-69.46 to 2.13	#	0.0629
B: DMSO/KH-103 vs. F: DEX/KH-103	-3.67	-39.46 to 32.13	ns	0.8271
D: DEX/DEX+KH-103 vs. F: DEX/KH-103	30.00	-5.79 to 65.79	#	0.0928

Supplementary Table. 1_Summary of Fisher's LSD multiple comparison of the cytosolic fraction.

Fisher's LSD	Mean difference	95% CI of difference	Summary	P-Value
A: DMSO/DMSO vs. C: DEX/DEX	50.67	16.84 to 84.49	**	0.0068
A: DMSO/DMSO vs. E: DEX/DMSO	44.00	10.18 to 77.82	*	0.0150
A: DMSO/DMSO vs. B: DMSO/KH-103	95.67	61.84 to 129.50	****	< 0.0001
A: DMSO/DMSO vs. D: DEX/DEX+KH-103	63.00	29.18 to 96.82	**	0.0016
A: DMSO/DMSO vs. F: DEX/KH-103	96.00	62.18 to 129.80	****	< 0.0001
C: DEX/DEX vs. E: DEX/DMSO	-6.67	-40.49 to 27.16	ns	0.6752
C: DEX/DEX vs. B: DMSO/KH-103	45.00	11.18 to 78.82	*	0.0134
C: DEX/DEX vs. D: DEX/DEX+KH-103	12.33	-21.49 to 46.16	ns	0.4423
C: DEX/DEX vs. F: DEX/KH-103	45.33	11.51 to 79.16	*	0.0128
E: DEX/DMSO vs. B: DMSO/KH-103	51.67	17.84 to 85.49	**	0.0060
E: DEX/DMSO vs. D: DEX/DEX+KH-103	19.00	-14.82 to 52.82	ns	0.2444
E: DEX/DMSO vs. F: DEX/KH-103	52.00	18.18 to 85.82	**	0.0058
B: DMSO/KH-103 vs. D: DEX/DEX+KH-103	-32.67	-66.49 to 1.16	#	0.0571
B: DMSO/KH-103 vs. F: DEX/KH-103	0.33	-33.49 to 34.16	ns	0.9832
D: DEX/DEX+KH-103 vs. F: DEX/KH-103	33.00	-0.82 to 66.82	#	0.0550

Supplementary Table. 2_Summary of Fisher's LSD multiple comparison of the membrane fraction.

Fisher's LSD	Mean difference	95% CI of difference	Summary	P-Value
A: DMSO/DMSO vs. C: DEX/DEX	-491.00	-567.10 to -414.90	****	< 0.0001
A: DMSO/DMSO vs. E: DEX/DMSO	-234.30	-310.40 to -158.30	****	< 0.0001
A: DMSO/DMSO vs. B: DMSO/KH-103	65.00	-9.68 to 139.70	#	0.0822
A: DMSO/DMSO vs. D: DEX/DEX+KH-103	-228.30	-304.40 to -152.30	****	< 0.0001
A: DMSO/DMSO vs. F: DEX/KH-103	64.67	-11.39 to 140.70	#	0.0881
C: DEX/DEX vs. E: DEX/DMSO	256.70	180.60 to 332.70	****	< 0.0001
C: DEX/DEX vs. B: DMSO/KH-103	571.50	486.50 to 656.50	****	< 0.0001
C: DEX/DEX vs. D: DEX/DEX+KH-103	262.70	186.60 to 338.70	****	< 0.0001
C: DEX/DEX vs. F: DEX/KH-103	555.70	479.60 to 631.70	****	< 0.0001
E: DEX/DMSO vs. B: DMSO/KH-103	314.80	229.80 to 399.90	****	< 0.0001
E: DEX/DMSO vs. D: DEX/DEX+KH-103	6.00	-70.06 to 82.06	ns	0.8653
E: DEX/DMSO vs. F: DEX/KH-103	299.00	222.90 to 375.10	****	< 0.0001
B: DMSO/KH-103 vs. D: DEX/DEX+KH-103	-308.80	-393.90 to -223.80	****	< 0.0001
B: DMSO/KH-103 vs. F: DEX/KH-103	-15.83	-100.90 to 69.20	ns	0.6898
D: DEX/DEX+KH-103 vs. F: DEX/KH-103	293.00	216.90 to 369.10	****	< 0.0001

Supplementary Table. 3_Summary of Fisher's LSD multiple comparison of the nuclear fraction.

transcriptional GR isoforms	one-way ANOVA	Holm-Šídák's: DMSO vs.	Summary
GR-α (N)	F(2, 6) = 6.81, p = 0.0286	KH-103: p = 0.0231	*
		dTAG13: p = 0.0424	*
GR- γ (N)	F(2, 6) = 13.49, p = 0.0060	KH-103: p = 0.0292	*
		dTAG13: p = 0.0041	**
GR-β (N)	F(2, 6) = 7.82, p = 0.0213	KH-103: p = 0.8207	ns
		dTAG13: p = 0.0325	*
GR-P (N)	F(2, 6) = 6.60, p = 0.0305	KH-103: p = 0.4922	ns
		dTAG13: p = 0.0684	#
GR-A (N)	F(2, 6) = 20.15, p = 0.0022	KH-103: p = 0.0120	*
		dTAG13: p = 0.0820	#
translational GR isoforms	one-way ANOVA	Holm-Šídák's: DMSO vs.	Summary
GR-α (C)	F(2, 6) = 9.08, p = 0.0153	KH-103: p = 0.0222	*
		dTAG13: p = 0.0127	*
GR-α-C3 (C)	F(2, 6) = 46.42, p = 0.0002	KH-103: p = 0.0002	***
		dTAG13: p = 0.0002	***
GR-α-D3 (C)	F(2, 6) = 101.30, p = < 0.0001	KH-103: p = < 0.0001	****
		dTAG13: p = < 0.0001	****
SARS-CoV2-MPro	one-way ANOVA	Holm-Šídák's: DMSO vs.	Summary
Mpro (C)	F(2, 8) = 4.36, p = 0.0524	KH-103: p = 0.9898	ns
		dTAG13: p = 0.0825	#
Mpro (N)	F(2, 8) = 16.96, p = 0.0013	KH-103: p = 0.3505	ns
		dTAG13: p = 0.0065	**

Supplementary Table. 4_Summary of statistics for GR isoforms and SARS-CoV2-MPro as assessed by immuno blotting upon PROTAC mediated depletion.

	DEX 18 h		DEX 2 h		MIF		CORT113176		KH-103	
gene name	LogFC	FDR	LogFC	FDR	LogFC	FDR	LogFC	FDR	LogFC	FDR
CYP2S1	-1.80	0.0000	-0.12	0.3300	-0.54	0.0000	-0.22	0.0160	-0.11	1
CYP4F22	5.40	0.0000	1.50	0.0880	-0.11	0.9800	-0.68	0.7700	0.42	1
CYP26B1	-0.99	0.0000	-0.52	0.0000	-0.21	0.2100	-0.34	0.0032	-0.20	1
CYP51A1	0.51	0.0000	0.02	0.9300	-0.06	0.7900	0.01	0.9700	0.04	1
CYP24A1	-0.63	0.0000	-0.74	0.0000	-0.03	0.9500	-0.04	0.8400	-0.01	1
CYP1B1	-0.87	0.0000	-0.39	0.0044	0.13	0.7100	2.10	0.0000	0.00	1
CYP4F11	-1.20	0.0000	-0.09	0.8600	-0.15	0.8000	0.01	0.9900	-0.13	1
CYP1A1	1.50	0.0000	0.85	0.0038	0.15	0.9000	4.50	0.0000	0.17	1
CYP26A1	-1.40	0.0000	-1.30	0.0000	-0.48	0.2400	-0.49	0.1600	-0.56	1
CYP3A5	0.49	0.0000	0.35	0.0075	0.02	0.9800	0.18	0.3900	0.21	1
CYP4F12	0.59	0.0000	0.43	0.0087	0.40	0.0480	0.33	0.1100	0.28	1
CYP27C1	-0.69	0.0009	-0.42	0.1500	-0.04	0.9800	-0.10	0.8700	-0.23	1
CYP2R1	0.30	0.0062	-0.01	0.9800	0.22	0.3000	0.06	0.8700	0.08	1
CYP4F2	-0.94	0.0270	0.42	0.5700	-0.77	0.3300	-0.38	0.6700	-0.35	1
CYP4F3	0.18	0.0430	0.40	0.0000	-0.09	0.7500	0.02	0.9500	0.93	1
CYP20A1	0.24	0.0470	0.11	0.7100	0.07	0.8900	0.16	0.5000	0.94	1
not significant	trend # < 0.1	significant FDR								

Supplementary Table. 5_Summary of differential expression analysis for CYPs genes obtained from the RNAseq experiment. All the listed CYPs genes were significantly changed in the DEX 18 h condition. colour labels green: genes selected for boxplot display in Supplementary Fig. 8, red: FDR statistically significant, yellow: FDR # < 0.1, grey: FDR not significant > 0.1).

Supplementary Methods

1. General Information 36
2. Experimental Procedures and Spectroscopic Data 37

1. General Information

1.1 General Methods

All non-aqueous reactions were performed in flame-dried glassware sealed with a rubber septum under an atmosphere of dry nitrogen. Transfer of anhydrous solvents or reagents was executed with syringes which were purged three times with nitrogen prior to use. Reactions were stirred magnetically and monitored by thin layer chromatography (TLC). TLC was performed on Merck silica gel F^{254} TLC glass plates and visualized by ultraviolet light (UV) light and stained with aqueous potassium permanganate ($KMnO_4$) followed by heating. Organic solutions were concentrated by rotary evaporation at 40 °C at the appropriate pressure. Crude products were absorbed onto Silicycle SiliaFlash® Silica Gel F60 and purified by flash column chromatography using Silicycle SiliaFlash® Silica Gel F60 under 0.3–0.5 bar overpressure. The yields given refer to purified compounds and spectroscopically pure compounds unless stated otherwise.

1.2 Solvents and Reagents

All reagents were purchased from commercial suppliers (ABCR, ACROS, Sigma Aldrich, Fluka, TCI, Strem, Alfa, Combi-Blocks or Fluorochem) and used without further purification. Anhydrous solvents over molecular sieves were purchase from Acros and used as received. Et_3N and DIPEA were freshly distilled from CaH_2 under an atmosphere of dry nitrogen. Deuterated solvents were obtained from Cambridge Isotope Laboratories, Apollo Scientific, or Sigma-Aldrich.

1.3 Analytics

NMR Spectroscopy. Nuclear magnetic resonance (NMR) data were recorded on a Bruker AV400, Bruker DRX400, Bruker AV500 or Bruker AVIII (600 MHz with cryoprobe) spectrometer at 298 K. Chemical shifts (δ) are reported in ppm with the residual solvent peak employed as internal standard ($CHCl_3$ at 7.26 ppm (^{1}H-NMR) and 77.16 ppm (^{13}C-NMR), CH_3OH at 3.31 ppm (^{1}H-NMR) and 49.0 ppm (^{13}C-NMR) and $(CH_3)_2CO$ at 2.05 ppm (^{1}H-NMR) and 29.84 ppm and 206.26 ppm (^{13}C-NMR). Signals are reported as s = singlet, d = doublet, t = triplet, q = quartet, m = multiplet or unresolved, br = broad signal. Coupling constant(s) are given in Hz. All ^{13}C-NMR spectra were recorded with broadband ^{1}H-decoupling.

Infrared Spectroscopy. Infrared spectra (IR) were recorded as thin films of neat compounds on a Perkin Elmer Two-FT-IR spectrometer. Only absorptions of characteristic peaks are reported in wavenumbers (cm^{-1}).

Mass Spectrometry. Mass spectrometric (MS) analyses were performed as high-resolution ESI measurements by the mass spectrometry service of the Laboratorium für Organische Chemie at ETH Zürich by Mr. Louis Bertschi, Mr. Daniel Wirz, and Mr. Michael Meier.

Optical Rotation. Specific rotations (α) were recorded on a Jasco P-2000 digital polarimeter using a cuvette with 10 cm cell length and a volume of 1 mL. The concentration c = 1 corresponds to 10 mg/mL.

2. Experimental Procedures and Spectroscopic Data

Synthesis of SI-2

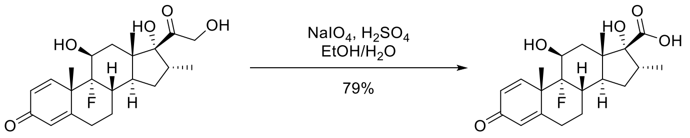

To a solution of **SI-1** (2.0 g, 5.1 mmol, 1.0 equiv) in ethanol (400 mL) was added water (157 mL) followed by sodium periodate (1.3 g, 6.0 mmol, 1.2 equiv) and sulfuric acid (2 M, 10.2 mL, 20.4 mmol, 4.00 equiv). After stirring at r.t. overnight, the reaction mixture was concentrated *in vacuo*. Water and brine were added and the reaction mixture was basified to a pH of 12 with aq. NaOH to dissolve the white precipitate. The aqueous layer was washed with CH_2Cl_2, acidified with 1 M $NaHSO_4$ to a pH of 3 and extracted with EtOAc. The combined organic layers were washed with brine, dried over Na_2SO_4, filtered, and concentrated *in vacuo* to yield **SI-2** (1.5 g, 79% yield) as a white solid.

^{1}H-NMR (500 MHz, CD_3OD): δ = 7.42 (d, *J* = 10.2 Hz, 1H), 6.28 (dd, *J* = 10.1, 1.9 Hz, 1H), 6.08 (d, *J* = 1.8 Hz, 1H), 4.27 – 4.22 (m, 1H), 3.04 – 2.96 (m, 1H), 2.78 – 2.66 (m, 1H), 2.53 – 2.36 (m, 2H), 2.19 – 2.10 (m, 2H), 1.93 – 1.84 (m, 1H), 1.75 (dt, *J* = 13.1, 11.0 Hz, 1H), 1.62 – 1.46 (m, 5H), 1.26 – 1.17 (m, 1H), 1.14 (s, 3H), 0.94 (dd, *J* = 7.2, 0.8 Hz, 3H) ppm.
^{13}C-NMR (126 MHz, CD_3OD): δ = 189.0, 176.9, 171.2, 156.1, 129.8, 125.1, 103.2, 101.8, 87.7, 73.1 (d, *J* = 37.3 Hz), 50.3 (d, *J* = 22.6 Hz), 44.3, 37.2, 37.0, 35.7 (d, *J* = 19.3 Hz), 33.6, 32.3, 28.8, 23.6 (d, *J* = 5.7 Hz), 17.7, 15.4 ppm.
^{19}F NMR (376 MHz, CD_3OD): δ = -166.08 ppm.
IR (neat): 3442, 2943, 2873, 1079, 1660, 1602, 1452, 1395, 1377, 1353, 1300, 1242, 1035, 892 cm^{-1}.
HRMS (ESI): *m/z* calcd. for $C_{21}H_{28}FO_5$ $[M+H]^+$ 379.1915, found 379.1916.
$[\alpha]^{25}_D$ = 55.1 (c = 1.0, CH_3OH).

Synthesis of SI-4

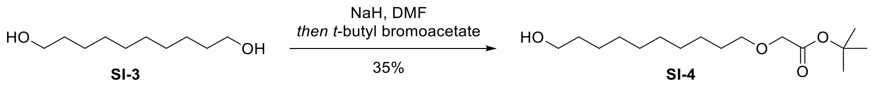

Sodium hydride (6.77 g, 169 mmol, 1.00 equiv) was added to a solution of **SI-3** (29.5 g, 169 mmol, 1.00 equiv) in anhydrous DMF (300 mL) at r.t. After stirring for 1 h at r.t., *t*-butyl bromoacetate (25.0 mL, 169 mmol, 1.00 equiv) was added slowly and cautiously at r.t. and in case of a strong rise in temperature, the reaction solution was cooled with a water bath. The resulting yellow reaction solution was stirred overnight at ambient temperature, after which residual sodium hydride was quenched by addition of water. The aqueous layer was extracted three times with ether:hexanes (2:1), the combined organic layers were washed with brine, dried over Na_2SO_4, filtered, and concentrated *in vacuo*. Column chromatography (eluent: 20% EtOAc/hexanes) yielded **SI-4** (16.9 g, 35%) as a colorless oil.

^{1}H-NMR (400 MHz, $CDCl_3$): δ = 3.94 (s, 2H), 3.63 (t, J = 6.7 Hz, 2H), 3.49 (t, J = 6.7 Hz, 2H), 1.65 – 1.51 (m, 4H), 1.47 (s, 9H), 1.39 – 1.25 (m, 12H) ppm.
^{13}C-NMR (101 MHz, $CDCl_3$): δ = 170.0, 81.6, 72.0, 68.9, 63.2, 32.9, 29.8, 29.6, 29.6, 29.6, 29.5, 28.3, 26.2, 25.9 ppm.
IR (neat): 3450, 2928, 2856, 1750, 1458, 1368, 1226, 1162, 1136, 11057, 847, 732 cm^{-1}.
HRMS (ESI): *m/z* calcd. for $C_{16}H_{32}NaO_4$ [M+Na]$^+$ 311.2193, found 311.2199.

Synthesis of SI-6

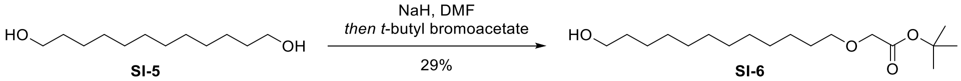

Sodium hydride (0.81 g, 20 mmol, 1.0 equiv) was added to a solution of **SI-5** (4.1 g, 20 mmol, 1.0 equiv) in anhydrous DMF (37 mL) at r.t. After stirring for 1 h at r.t., *t*-butyl bromoacetate (3 mL, 20 mmol, 1.0 equiv) was added slowly and cautiously at r.t. and in case of a strong rise in temperature, the reaction solution was cooled with a water bath. The resulting yellow reaction solution was stirred overnight at ambient temperature, after which residual sodium hydride was quenched by addition of water. The aqueous layer was extracted three times with ether:hexanes (2:1), the combined organic layers were washed with brine, dried over Na_2SO_4, filtered, and concentrated *in vacuo*. Column chromatography (eluent: 15% EtOAc/hexanes) yielded **SI-6** (1.9 g, 29%) as a colorless oil.

^{1}H-NMR (400 MHz, $CDCl_3$): δ = 3.93 (s, 2H), 3.62 (dd, *J* = 7.3, 6.1 Hz, 2H), 3.48 (t, *J* = 6.7 Hz, 2H), 1.64 – 1.50 (m, 4H), 1.46 (s, 9H), 1.38 – 1.20 (m, 16H) ppm.
^{13}C-NMR (101 MHz, $CDCl_3$): δ = 170.0, 81.5, 72.0, 68.9, 63.2, 32.9, 29.8, 29.7, 29.7, 29.6, 29.5, 28.2, 26.2, 25.9 ppm.
IR (neat): 3430, 2925, 2854, 1750, 1458, 1368, 1300, 1225, 1161, 1135, 11056, 846, 731 cm^{-1}.
HRMS (ESI): *m/z* calcd. for $C_{18}H_{36}NaO_4$ $[M+Na]^+$ 339.2506, found 339.2506.

Synthesis of SI-7

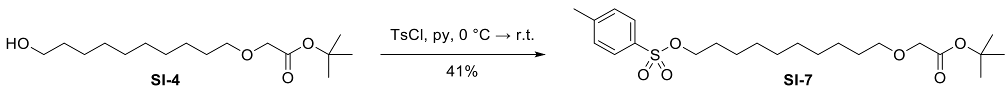

TsCl (9.9 g, 52 mmol, 3.0 equiv) was added to a solution of **SI-4** (5.0 g, 17 mmol, 1.0 equiv) in pyridine (16.5 mL) at 0 °C. The resulting reaction solution was allowed to warm to r.t. over 2 h before it was diluted with EtOAc and washed with sat. aq. NH_4Cl solution, water and brine. The reaction mixture was dried over Na_2SO_4, filtered, and concentrated *in vacuo*. Column chromatography (eluent: 5% EtOAc/hexanes) yielded **SI-7** (3.14 g, 41%) as a colorless oil.

^{1}H-NMR (400 MHz, $CDCl_3$): δ = 7.78 (d, *J* = 8.2 Hz, 2H), 7.34 (d, *J* = 8.4 Hz, 2H), 4.01 (t, *J* = 6.1 Hz, 2H), 3.93 (s, 2H), 3.49 (t, *J* = 6.7 Hz, 2H), 2.44 (s, 3H), 1.65 – 1.56 (m, 4H), 1.47 (s, 9H), 1.35 – 1.16 (m, 12H) ppm.
^{13}C-NMR (101 MHz, $CDCl_3$): δ = 170.0, 144.7, 133.4, 129.9, 128.0, 81.6, 71.9, 70.8, 68.9, 29.8, 29.5, 29.5, 29.4, 29.0, 28.9, 28.3, 26.1, 25.4, 21.8 ppm.
IR (neat): 2928, 2856, 1747, 1727, 1365, 1225, 1189, 1176, 1135, 1098, 959, 934, 815, 664 cm^{-1}.
HRMS (ESI): *m/z* calcd. for $C_{23}H_{38}NaO_6S$ $[M+Na]^+$ 465.2281, found 465.2280.

Synthesis of SI-8

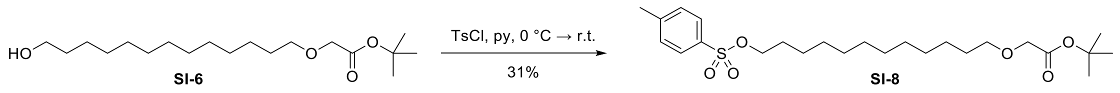

TsCl (3.4 g, 18 mmol, 3.0 equiv) was added to a solution of **SI-6** (1.9 g, 6.0 mmol, 1.0 equiv) in pyridine (5.7 mL) at 0 °C. The resulting reaction solution was allowed to warm to r.t. over 2 h before it was diluted with EtOAc and washed with sat. aq. NH_4Cl solution, water and brine. The reaction mixture was dried over Na_2SO_4, filtered, and concentrated *in vacuo*. Column chromatography (eluent: 8% EtOAc/hexanes) yielded **SI-8** (0.88 g, 31%) as a colorless oil.

^{1}H-NMR (400 MHz, $CDCl_3$): δ = 7.81 – 7.76 (m, 2H), 7.37 – 7.31 (m, 2H), 4.01 (t, *J* = 6.5 Hz, 2H), 3.94 (s, 2H), 3.50 (t, *J* = 6.7 Hz, 2H), 2.45 (s, 3H), 1.67 – 1.56 (m, 4H), 1.48 (s, 9H), 1.40 – 1.15 (m, 16H) ppm.

^{13}C-NMR (101 MHz, $CDCl_3$): δ = 170.0, 144.7, 133.4, 129.9, 128.0, 81.6, 72.0, 70.9, 68.9, 29.8, 29.7, 29.7, 29.6, 29.5, 29.1, 29.0, 28.3, 26.2, 25.5, 21.8 ppm.

IR (neat): 2927, 2855, 1748, 1599, 1366, 1226, 1189, 1177, 1135, 948, 815 cm^{-1}.

HRMS (ESI): *m/z* calcd. for $C_{25}H_{42}NaO_6S$ $[M+Na]^+$ 493.2594, found 493.2590.

Synthesis of SI-9

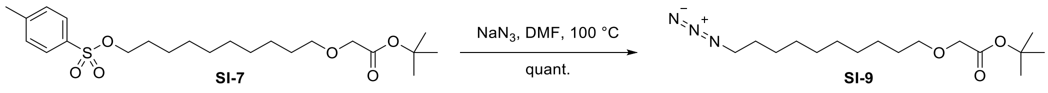

Sodium azide (2.3 g, 35 mmol, 5.0 equiv) was added to a solution of **SI-7** (3.1 g, 7.1 mmol, 1.0 equiv) in anhydrous DMF (52 mL). After stirring the resulting yellowish reaction mixture at 100 °C for 3 h, it was allowed to cool down to r.t. and diluted with ether and water. The reaction mixture was extracted three times with ether:hexanes (2:1) and the combined organic layers were washed with brine, dried over Na_2SO_4, filtered, and concentrated *in vacuo*. Column chromatography (eluent: 5% EtOAc/hexanes) yielded azide **SI-9** (2.2 g, quant.) as a yellowish oil.

^{1}H-NMR (400 MHz, $CDCl_3$): δ = 3.94 (d, *J* = 0.4 Hz, 2H), 3.50 (t, *J* = 6.7 Hz, 2H), 3.25 (t, *J* = 7.0 Hz, 2H), 1.65 – 1.54 (m, 4H), 1.47 (s, 9H), 1.40 – 1.23 (m, 12H) ppm.
^{13}C-NMR (101 MHz, $CDCl_3$): δ = 170.0, 81.6, 72.0, 68.9, 51.6, 29.8, 29.6, 29.6, 29.5, 29.3, 29.0, 28.3, 26.8, 26.2 ppm.
IR (neat): 2928, 2856, 2093, 1749, 1729, 1457, 1368, 1256, 1225, 1134, 847 cm^{-1}.
HRMS (ESI): *m/z* calcd. for $C_{16}H_{31}N_3NaO_3$ $[M+Na]^+$ 336.2258, found 336.2254.

Synthesis of SI-10

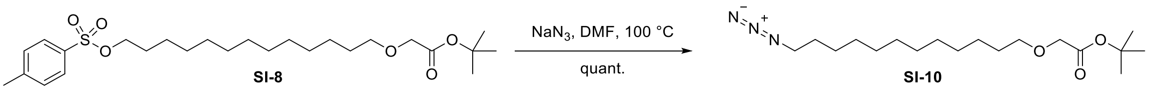

Sodium azide (0.22 g, 3.4 mmol, 5.0 equiv) was added to a solution of **SI-8** (0.32 g, 0.68 mmol, 1.0 equiv) in anhydrous DMF (6.7 mL). After stirring the resulting yellowish reaction mixture at 100 °C for 3 h, it was allowed to cool down to r.t. and diluted with ether and water. The reaction mixture was extracted three times with ether:hexanes (2:1) and the combined organic layers were washed with brine, dried over Na_2SO_4, filtered, and concentrated *in vacuo*. Column chromatography (eluent: 5% EtOAc/hexanes) yielded azide **SI-10** (0.23 g, quant.) as a yellowish oil.

^{1}H-NMR (400 MHz, $CDCl_3$): δ = 3.94 (s, 2H), 3.50 (t, *J* = 6.7 Hz, 2H), 3.25 (t, *J* = 7.0 Hz, 2H), 1.65 – 1.55 (m, 4H), 1.48 (s, 9H), 1.40 – 1.21 (m, 16H) ppm.
^{13}C-NMR (101 MHz, $CDCl_3$): δ = 170.0, 81.6, 72.0, 69.0, 51.6, 29.8, 29.7, 29.65, 29.6, 29.3, 29.0, 28.3, 26.9, 26.2 ppm.
IR (neat): 2926, 2855, 2094, 1750, 1730, 1457, 1368, 1224, 1163, 1134, 942 cm^{-1}.
HRMS (ESI): *m/z* calcd. for $C_{18}H_{35}N_3NaO_3$ $[M+Na]^+$ 364.2571, found 364.2570.

Synthesis of SI-12

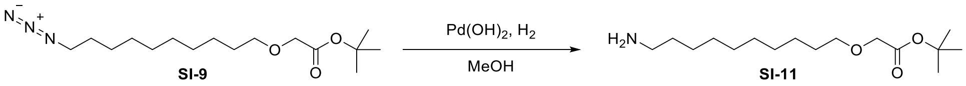

SI-9 (1.0 g, 3.2 mmol, 1.0 equiv) in anhydrous MeOH (32 mL) was added to $Pd(OH)_2$ (0.44 g, 0.64 mmol, 0.20 equiv). The reaction mixture was cautiously saturated by hydrogen at atmospheric pressure and stirred for 1.5 h at r.t. Subsequent filtration over celite followed by washing with EtOAc gave crude product **SI-11** which was used in the next step without further purification.

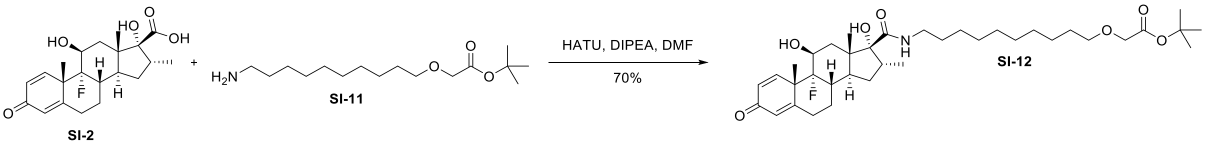

SI-2 (50 mg, 0.13 mmol, 1.0 equiv), **SI-11** (57 mg, 0.20 mmol, 1.5 equiv) and HATU (52.9 mg, 0.139 mmol, 1.05 equiv) were dissolved in anhydrous DMF (1.3 mL) and DIPEA (0.07 mL, 0.40 mmol, 3.0 equiv) was added. The resulting reaction mixture was stirred overnight after which it was concentrated *in vacuo.* Column chromatography (eluent: 20% EtOAc/hexanes) followed by preparative TLC (3:2 EtOAc/hexanes) yielded **SI-12** (60 mg, 70%) as a yellowish foam.

^{1}H-NMR (500 MHz, CD_3OD): δ = 7.42 (d, *J* = 10.1 Hz, 1H), 6.28 (dd, *J* = 10.1, 1.9 Hz, 1H), 6.08 (t, *J* = 1.7 Hz, 1H), 4.26 – 4.21 (m, 1H), 3.95 (s, 2H), 3.50 (t, *J* = 6.6 Hz, 2H), 3.26 (dt, *J* = 13.2, 7.2 Hz, 1H), 3.20 – 3.06 (m, 2H), 2.76 – 2.66 (m, 1H), 2.52 – 2.35 (m, 2H), 2.24 – 2.15 (m, 2H), 1.94 – 1.84 (m, 1H), 1.81 – 1.69 (m, 1H), 1.59 (s, 4H), 1.57 – 1.49 (m, 4H), 1.48 (s, 9H), 1.47 – 1.43 (m, 1H), 1.38 – 1.31 (m, 12H), 1.24 – 1.16 (m, 1H), 1.10 (s, 3H), 0.89 (d, *J* = 7.2 Hz, 3H) ppm.
^{13}C-NMR (126 MHz, CD_3OD): δ = 189.0, 175.4, 171.7, 171.1 (d, *J* = 1.8 Hz), 156.0, 129.8, 125.1, 103.3, 101.9, 88.1, 82.7, 73.0 (d, *J* = 37.5 Hz), 72.7, 69.5, 50.3 (d, *J* = 22.8 Hz), 44.8 (d, *J* = 1.6 Hz), 40.4, 36.9, 36.3, 35.7 (d, *J* = 19.3 Hz), 33.4, 32.3, 30.9, 30.6, 30.5, 30.4, 28.8 (d, *J* = 1.7 Hz), 28.4 (d, *J* = 1.5 Hz), 28.1, 27.1, 23.6 (d, *J* = 5.7 Hz), 17.8, 15.2 ppm.
^{19}F NMR (471 MHz, CD_3OD): δ = -165.79 ppm.
IR (neat): 3415, 2930, 2856, 1747, 1663, 1623, 1525, 1453, 1368, 1242, 1135 cm^{-1}.
HRMS (ESI): *m/z* calcd. for $C_{37}H_{58}FNNaO_7$ $[M+Na]^+$ 670.4090, found 670.4087.
$[\alpha]^{25}_D$ = 57.3 (c = 1.0, CH_3OH).

Synthesis of SI-14

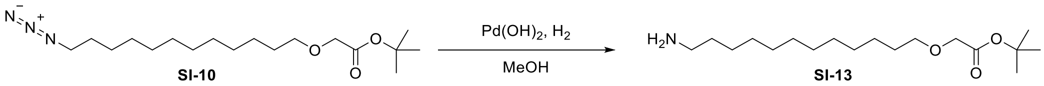

SI-10 (54 mg, 0.16 mmol, 1.0 equiv) in anhydrous MeOH (1.6 mL) was added to $Pd(OH)_2$ (22 mg, 0.03 mmol, 0.20 equiv). The reaction mixture was cautiously saturated by hydrogen at atmospheric pressure and stirred for 1.5 h at r.t. Subsequent filtration over celite followed by washing with EtOAc gave crude product **SI-13** which was used in the next step without further purification.

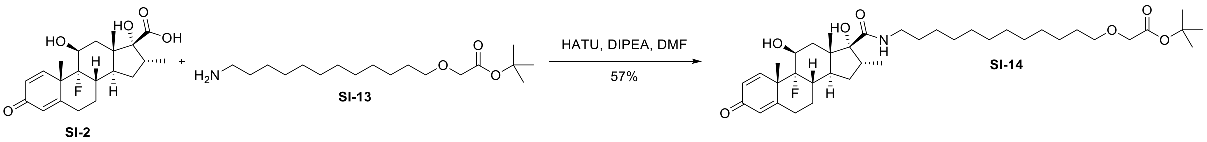

SI-2 (50 mg, 0.13 mmol, 1.0 equiv), **SI-13** (62.5 mg, 0.198 mmol, 1.50 equiv) and HATU (52.9 mg, 0.139 mmol, 1.05 equiv) were dissolved in anhydrous DMF (1.3 mL) and DIPEA (0.07 mL, 0.40 mmol, 3.0 equiv) was added. The resulting reaction mixture was stirred overnight after which it was concentrated *in vacuo.* Column chromatography (eluent: 30% EtOAc/hexanes) followed by preparative TLC (3:2 EtOAc/hexanes) yielded **SI-14** (51.1 mg, 57%) as a yellowish foam.

^{1}H-NMR (400 MHz, CD_3OD): δ = 7.42 (d, *J* = 10.1 Hz, 1H), 6.28 (dd, *J* = 10.1, 1.9 Hz, 1H), 6.08 (t, *J* = 1.7 Hz, 1H), 4.28 – 4.19 (m, 1H), 3.95 (s, 2H), 3.50 (t, *J* = 6.6 Hz, 2H), 3.31 – 3.21 (m, 1H), 3.19 – 3.06 (m, 2H), 2.78 – 2.66 (m, 1H), 2.53 – 2.34 (m, 2H), 2.26 – 2.12 (m, 2H), 1.94 – 1.84 (m, 1H), 1.75 (dt, *J* = 12.3, 10.7 Hz, 1H), 1.59 (s, 4H), 1.56 – 1.49 (m, 4H), 1.48 (s, 9H), 1.46 (d, *J* = 1.1 Hz, 1H), 1.39 – 1.25 (m, 16H), 1.26 – 1.16 (m, 1H), 1.10 (s, 3H), 0.89 (d, *J* = 7.3 Hz, 3H) ppm.
^{13}C-NMR (101 MHz, CD_3OD): δ = 187.7, 174.1, 170.4, 169.8 (d, *J* = 1.7 Hz), 154.7, 128.4, 123.7, 102.1, 100.4, 86.8, 81.3, 71.7 (d, *J* = 37.5 Hz), 71.3, 68.1, 49.0 (d, *J* = 22.8 Hz), 43.5, 39.0, 35.5, 34.9, 34.4 (d, *J* = 19.3 Hz), 32.0, 30.9, 29.5, 29.3 (d, *J* = 1.5 Hz), 29.2, 29.1, 29.0, 27.4, 27.0, 26.7, 25.7, 22.2 (d, *J* = 5.6 Hz), 16.4, 13.8 ppm.
^{19}F NMR (376 MHz, CD_3OD): δ = -165.87 ppm.
IR (neat): 3414, 2927, 2854, 1747, 1663, 1624, 1525, 1454, 1368, 1241, 1135 cm^{-1}.
HRMS (ESI): *m/z* calcd. for $C_{39}H_{62}FNNaO_7$ $[M+Na]^+$ 698.4403, found 698.4394.
$[\alpha]^{25}_D$ = 61.1 (c = 0.5, CH_3OH).

Synthesis of KH-103

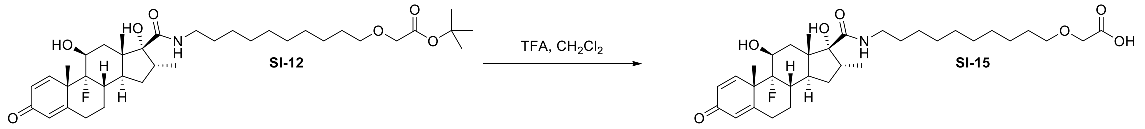

SI-12 (60 mg, 0.09 mmol, 1.0 equiv) was dissolved in anhydrous CH_2Cl_2 (0.45 mL) and TFA (0.45 mL) was added. The resulting reaction mixture was stirred at r.t. for 1.5 h after which it was diluted with toluene and concentrated *in vacuo*. Crude product **SI-15** was used in the next step without further purification.

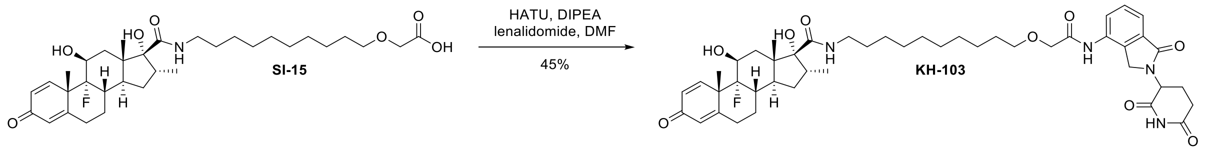

SI-15 (54.8 mg, 0.093 mmol, 1.00 equiv), lenalidomide (24 mg, 0.09 mmol, 1.0 equiv) and HATU (38.8 g, 0.102 mmol, 1.10 equiv) were dissolved in anhydrous DMF (0.75 mL) and DIPEA (48 μL, 0.28 mmol, 3.0 equiv) was added. The resulting reaction mixture was stirred overnight after which it was concentrated *in vacuo.* Column chromatography (eluent: grading from 0% to 10% MeOH/EtOAc) followed by preparative TLC (15% MeOH/EtOAc) yielded **KH-103** (35 mg, 45%) as a colorless oil.

^{1}H-NMR (400 MHz, CD_3OD): δ = 7.75 (dd, *J* = 7.9, 1.0 Hz, 1H), 7.69 (dd, *J* = 7.6, 1.0 Hz, 1H), 7.55 (t, *J* = 7.7 Hz, 1H), 7.41 (d, *J* = 10.1 Hz, 1H), 6.28 (dd, *J* = 10.1, 1.9 Hz, 1H), 6.07 (d, *J* = 1.8 Hz, 1H), 5.16 (m, 1H), 4.50 (d, *J* = 3.2 Hz, 2H), 4.26 – 4.18 (m, 1H), 4.13 (s, 2H), 3.61 (t, *J* = 6.6 Hz, 2H), 3.31 – 3.19 (m, 1H), 3.19 – 3.02 (m, 2H), 2.99 – 2.85 (m, 1H), 2.82 – 2.64 (m, 2H), 2.56 – 2.31 (m, 3H), 2.24 – 2.10 (m, 3H), 1.93 – 1.80 (m, 1H), 1.79 – 1.63 (m, 3H), 1.58 (s, 3H), 1.55 – 1.44 (m, 4H) 1.33 (s, 12H), 1.23 – 1.12 (m, 1H), 1.09 (s, 3H), 0.88 (d, *J* = 7.2 Hz, 3H) ppm.
^{13}C-NMR (101 MHz, CD_3OD): δ = 189.1, 175.4, 174.6, 172.1, 171.2, 171.2, 171.2, 171.0, 156.1, 136.9, 134.0, 133.7, 130.1, 129.8, 128.3, 125.1, 121.9, 103.6, 101.8, 88.2, 73.1, 73.0 (d, *J* = 37.4 Hz), 71.1, 53.7, 50.3 (d, *J* = 22.8 Hz), 44.8, 40.4, 36.9, 36.3, 35.8 (d, *J* = 19.4 Hz), 33.4, 32.4, 32.3, 30.8, 30.6, 30.6, 30.5, 30.5, 30.4, 28.8, 28.0, 27.1, 24.1, 23.6 (d, *J* = 5.7 Hz), 17.8, 15.2 ppm.
^{19}F NMR (376 MHz, CD_3OD): δ = -165.8 ppm.
^{1}H-NMR (600 MHz, $(CD_3)_2CO$): δ =9.83 (s, 1H), 8.97 (s, 1H), 7.92 (dt, *J* = 8.0, 1.0 Hz, 1H), 7.60 (dt, *J* = 7.5, 0.9 Hz, 1H), 7.52 (t, *J* = 7.7 Hz, 1H), 7.30 (d, *J* = 10.3 Hz, 1H), 7.07 (t, *J* = 6.0 Hz, 1H), 6.19 (dd, *J* = 10.1, 1.9 Hz, 1H), 6.00 (t, *J* = 1.8 Hz, 1H), 5.22 (dd, *J* = 13.4, 5.1 Hz, 1H), 4.51 (q, *J* = 16.6 Hz, 2H), 4.37 – 4.28 (m, 2H), 4.09 (s, 2H), 3.63 (t, *J* = 6.6 Hz, 2H), 3.28 – 3.20 (m, 1H), 3.20 – 3.12 (m, 2H), 3.05 – 2.95 (m, 1H), 2.83 – 2.75 (m, 1H), 2.75 – 2.67 (m, 1H), 2.58 – 2.40 (m, 2H), 2.39 – 2.33 (m, 1H), 2.26 – 2.17 (m, 3H), 1.90 – 1.83 (m, 1H), 1.78 – 1.69 (m, 1H), 1.69 – 1.62 (m, 2H), 1.60 (s, 3H), 1.54 (dd, *J*

= 14.2, 1.8 Hz, 1H), 1.52 – 1.45 (m, 3H), 1.45 – 1.38 (m, 2H), 1.38 – 1.25 (m, 10H), 1.20 – 1.14 (m, 1H), 1.12 (s, 3H), 0.90 (dd, *J* = 7.2, 1.1 Hz, 3H) ppm.

IR (neat): 3382, 2929, 2855, 1662, 1605, 1527, 1434, 1239, 1125, 893 cm^{-1}.

HRMS (ESI): *m/z* calcd. for $C_{46}H_{61}FN_4NaO_9$ $[M+Na]^+$ 855.4315, found 855.4307.

$[\alpha]^{25}_{D}$ = 37.1 (c = 1.0, CH_3OH).

Synthesis of KH-102

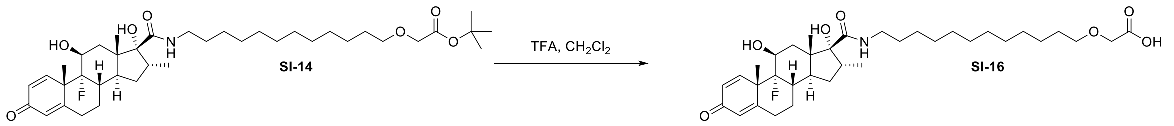

SI-14 (51.1 mg, 0.076 mmol, 1.00 equiv) was dissolved in anhydrous CH_2Cl_2 (0.37 mL) and TFA (0.37 mL) was added. The resulting reaction mixture was stirred at r.t. for 1.5 h after which it was diluted with toluene and concentrated *in vacuo*. Crude product **SI-16** was used in the next step without further purification.

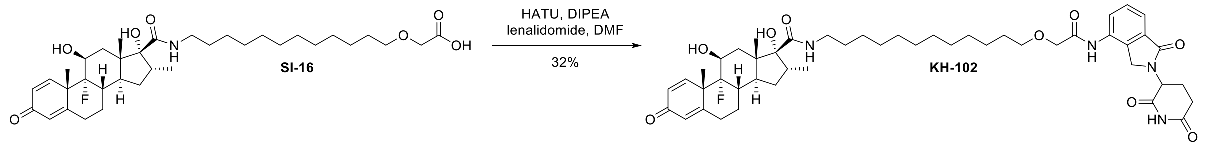

SI-16 (46.9 mg, 75.7 μmol, 1.00 equiv), lenalidomide (19.6 mg, 0.076 mmol, 1.00 equiv) and HATU (31.7 mg, 0.083 mmol, 1.10 equiv) were dissolved in anhydrous DMF (0.63 mL) and DIPEA (39 μL, 0.23 mmol, 3.0 equiv) was added. The resulting reaction mixture was stirred overnight after which it was concentrated *in vacuo.* Column chromatography (eluent: grading from 0% to 10% MeOH/EtOAc) followed by preparative TLC (15% MeOH/EtOAc) yielded **KH-102** (20.7 mg, 32%) as a white solid.

^{1}H-NMR (500 MHz, CD_3OD): δ = 7.75 (dd, *J* = 8.0, 1.2 Hz, 1H), 7.71 – 7.66 (m, 1H), 7.58 – 7.49 (m, 1H), 7.41 (dd, *J* = 10.1, 1.4 Hz, 1H), 6.31 – 6.26 (m, 1H), 6.07 (t, *J* = 1.7 Hz, 1H), 5.19 – 5.12 (m, 1H), 4.57 – 4.45 (m, 2H), 4.23 (dd, *J* = 12.2, 3.6 Hz, 1H), 4.13 (s, 2H), 3.61 (td, *J* = 6.7, 1.7 Hz, 2H), 3.29 – 3.22 (m, 1H), 3.19 – 3.05 (m, 2H), 2.96 – 2.85 (m, 1H), 2.82 – 2.65 (m, 2H), 2.54 – 2.34 (m, 3H), 2.25 – 2.12 (m, 3H), 1.88 (dd, *J* = 16.6, 9.7 Hz, 1H), 1.81 – 1.64 (m, 3H), 1.58 (s, 3H), 1.58 – 1.39 (m, 4H), 1.36 – 1.27 (m, 16H), 1.24 – 1.13 (m, 1H), 1.09 (s, 3H), 0.89 (d, *J* = 7.2 Hz, 3H) ppm.
^{13}C-NMR (126 MHz, CD_3OD): δ = 189.1, 175.5, 174.6, 172.1, 171.3, 171.2, 171.2, 171.0, 156.1, 136.9, 134.0, 133.7, 130.1, 129.8, 128.3, 125.1, 121.9, 103.4, 102.0, 88.2, 73.1, 73.1 (d, *J* = 37.8 Hz), 71.1, 53.7, 50.3 (d, *J* = 22.8 Hz), 44.8 (d, *J* = 1.5 Hz), 40.4, 36.9, 36.3, 35.8 (d, *J* = 19.3 Hz), 33.4, 32.4, 32.3, 30.8, 30.7, 30.7, 30.7, 30.6, 30.5, 30.4, 28.8, 28.1, 27.1, 24.1, 23.6 (d, *J* = 5.6 Hz), 17.8, 15.2 ppm.
^{19}F NMR (471 MHz, CD_3OD): δ = -165.8 ppm.
^{1}H-NMR (600 MHz, $(CD_3)_2CO$): δ = 9.79 (s, 1H), 8.96 (s, 1H), 7.94 – 7.89 (m, 1H), 7.59 (dd, *J* = 7.5, 1.0 Hz, 1H), 7.52 (t, *J* = 7.7 Hz, 1H), 7.30 (d, *J* = 10.1 Hz, 1H), 7.09 – 6.99 (m, 1H), 6.18 (dd, *J* = 10.1, 1.9 Hz, 1H), 5.99 (t, *J* = 1.8 Hz, 1H), 5.21 (dd, *J* = 13.4, 5.1 Hz, 1H), 4.51 (q, *J* = 16.6 Hz, 2H), 4.36 – 4.29 (m, 2H), 4.09 (s, 2H), 3.62 (t, *J* = 6.6 Hz, 2H), 3.29 – 3.21 (m, 1H), 3.21 – 3.10 (m, 2H), 3.01 (ddd, *J* = 17.6, 13.7, 5.4 Hz, 1H), 2.81 – 2.75 (m, 1H), 2.71 (tdd, *J* = 13.7, 6.1, 1.9 Hz, 1H), 2.59 – 2.41 (m, 2H), 2.35 (ddd, *J* = 13.8, 5.2, 1.9 Hz, 1H), 2.25 – 2.16 (m, 3H), 1.90 – 1.80 (m, 1H), 1.78 – 1.69 (m, 1H), 1.69 – 1.62 (m, 2H), 1.60 (s, 3H), 1.54 (dd, *J* = 14.4, 1.9 Hz, 1H), 1.52 – 1.45 (m, 3H), 1.45 – 1.38

(m, 2H), 1.38 – 1.25 (m, 14H), 1.21 – 1.15 (m, 1H), 1.12 (d, *J* = 0.7 Hz, 3H), 0.90 (dd, *J* = 7.3, 0.6 Hz, 3H) ppm.

IR (neat): 3384, 2927, 2854, 1663, 1606, 1526, 1434, 1291, 1240, 1125, 844 cm^{-1}.

HRMS (ESI): *m/z* calcd. for $C_{48}H_{65}FN_4NaO_9$ $[M+Na]^+$ 883.4628, found 883.4632.

$[\alpha]^{25}_D$ = 33.8 (c = 0.5, CH_3OH).

Synthesis of SI-19

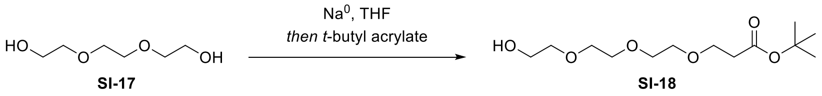

Sodium (9.7 mg, 0.42 mmol, 0.03 equiv) was added to a solution of **SI-17** (7.4 g, 6.6 mL, 49 mmol, 3.5 equiv) in anhydrous THF (26.6 mL) at r.t. After stirring the resulting reaction solution for 1 h and upon complete dissolution of sodium, *t*-butyl acrylate (2.00 mL, 13.8 mmol, 1.00 equiv) was added dropwise. The reaction solution was stirred overnight and concentrated *in vacuo*. Crude product **SI-18** was used in the next step without further purification.

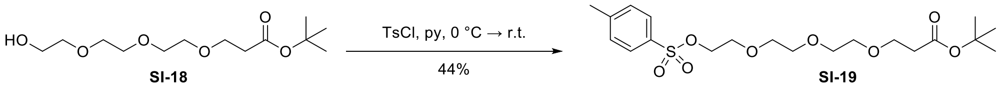

TsCl (1.26 mL, 8.62 mmol, 3.00 equiv) was added to a solution of **SI-18** (800 mg, 2.87 mmol, 1.00 equiv) in pyridine (2.6 mL) at 0 °C. The resulting reaction solution was allowed to warm to r.t. overnight before it was diluted with EtOAc and washed with sat. aq. NH_4Cl solution, water and brine. The reaction mixture was dried over Na_2SO_4, filtered, and concentrated *in vacuo*. Column chromatography (eluent: grading from 25% to 100% EtOAc/hexanes) yielded **SI-19** (548 mg, 44% yield) as a colorless oil.

^{1}H-NMR (400 MHz, $CDCl_3$): δ = 7.77 (d, *J* = 8.1 Hz, 2H), 7.32 (d, *J* = 8.0 Hz, 2H), 4.15 – 4.10 (m, 2H), 3.72 – 3.63 (m, 4H), 3.60 – 3.52 (m, 8H), 2.50 – 2.44 (m, 2H), 2.42 (s, 3H), 1.41 (s, 9H) ppm.
^{13}C-NMR (101 MHz, $CDCl_3$): δ =170.9, 144.9, 133.1, 129.9, 128.0, 80.6, 70.8, 70.6, 70.4, 69.3, 68.7, 67.0, 36.3, 28.2, 21.7 ppm.
IR (neat): 2977, 2872, 1726, 1598, 1455, 1393, 1356, 1280, 1252, 1189, 1157, 1175, 1097, 1018, 919, 816, 774 cm^{-1}.
HRMS (ESI): *m/z* calcd. for $C_{20}H_{36}NO_8S$ $[M+NH_4]^+$ 450.2156, found 450.2153.

Synthesis of SI-22

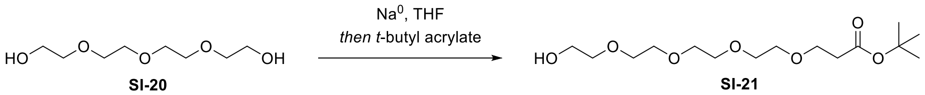

Sodium (8.40 mg, 365 μmol, 0.0265 equiv) was added to a solution of **SI-20** (8.03 g, 7.14 mL, 41.3 mmol, 3.00 equiv) in anhydrous THF (20 mL) at r.t. After stirring the resulting reaction solution for 1 h and upon complete dissolution of sodium, *t*-butyl acrylate (2.00 mL, 13.8 mmol, 1.00 equiv) was added dropwise. The reaction solution was stirred overnight and concentrated *in vacuo*. Crude product **SI-21** was used in the next step without further purification.

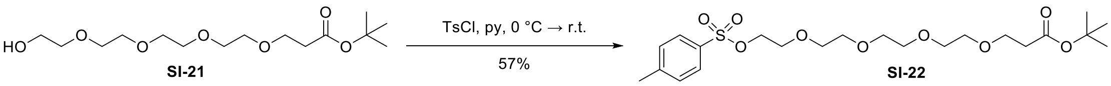

TsCl (1.6 g, 8.5 mmol, 3.0 equiv) was added to a solution of **SI-21** (913 mg, 2.83 mmol, 1.00 equiv) in pyridine (2.7 mL) at 0 °C. The resulting reaction solution was allowed to warm to r.t. overnight before it was diluted with EtOAc and washed with sat. aq. NH_4Cl solution, water and brine. The reaction mixture was dried over Na_2SO_4, filtered, and concentrated *in vacuo*. Column chromatography (eluent: grading from 30% to 100% EtOAc/hexanes) yielded **SI-22** (769 mg, 57% yield) as a colorless oil.

^{1}H-NMR (400 MHz, $CDCl_3$): δ = 7.78 (d, *J* = 8.3 Hz, 2H), 7.32 (d, *J* = 8.0 Hz, 2H), 4.16 – 4.11 (m, 2H), 3.72 – 3.64 (m, 4H), 3.62 – 3.53 (m, 12H), 2.47 (t, *J* = 6.6 Hz, 2H), 2.43 (s, 3H), 1.42 (s, 9H) ppm.
^{13}C-NMR (101 MHz, $CDCl_3$): δ = 171.0, 144.9, 133.1, 129.9, 128.1, 80.6, 70.8, 70.7, 70.7, 70.6, 70.6, 70.4, 69.3, 68.8, 67.0, 36.4, 28.2, 21.7 ppm.
IR (neat): 2871, 1727, 1598, 1455, 1357, 1290, 1251, 1189, 1176, 1097, 1018, 920, 816, 752 cm^{-1}.
HRMS (ESI): *m/z* calcd. for $C_{22}H_{36}NaO_9S$ $[M+Na]^+$ 499.1972, found 499.1958.

Synthesis of SI-23

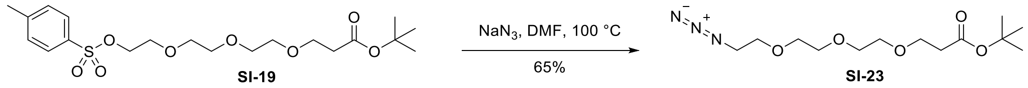

Sodium azide (0.526 g, 8.09 mmol, 3.50 equiv) was added to a solution of **SI-19** (1.00 g, 2.31 mmol, 1.00 equiv) in anhydrous DMF (6.0 mL). After stirring the resulting yellowish reaction mixture at r.t. overnight, it was diluted with ether and water. The reaction mixture was extracted three times with ether:hexanes (2:1) and the combined organic layers were washed with brine, dried over Na_2SO_4, filtered, and concentrated *in vacuo*. Column chromatography (eluent: 15% EtOAc/hexanes) yielded azide **SI-23** (458 mg, 65%) as a yellowish oil.

^{1}H-NMR (400 MHz, $CDCl_3$): δ =3.73 – 3.59 (m, 12H), 3.38 (t, *J* = 5.1 Hz, 2H), 2.50 (t, *J* = 6.6 Hz, 2H), 1.44 (s, 9H) ppm.
^{13}C-NMR (101 MHz, $CDCl_3$): δ = 171.1, 80.7, 70.9, 70.8, 70.8, 70.5, 70.2, 67.0, 50.8, 36.4, 28.2 ppm.
IR (neat): 2976, 2869, 2101, 1728, 1456, 1393, 1367, 1281, 1252, 1111, 999, 946, 848 cm^{-1}.
HRMS (ESI): *m/z* calcd. for $C_{13}H_{25}N_3NaO_5$ $[M+Na]^+$ 326.1686, found 326.1682.

Synthesis of SI-24

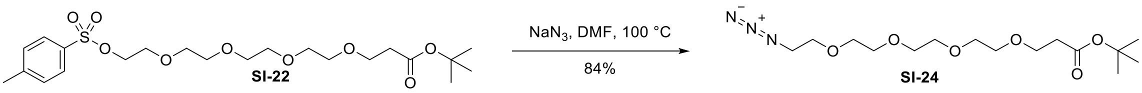

Sodium azide (477 mg, 7.34 mmol, 3.50 equiv) was added to a solution of **SI-22** (1.0 g, 2.1 mmol, 1.0 equiv) in anhydrous DMF (5.0 mL). After stirring the resulting yellowish reaction mixture at r.t. overnight, it was diluted with ether and water. The reaction mixture was extracted three times with ether:hexanes (2:1) and the combined organic layers were washed with brine, dried over Na_2SO_4, filtered, and concentrated *in vacuo*. Column chromatography (eluent: grading from 20% to 25% EtOAc/hexanes) yielded azide **SI-24** (612 mg, 84%) as a yellowish oil.

^{1}H-NMR (400 MHz, $CDCl_3$): δ = 3.72 – 3.64 (m, 13H), 3.64 – 3.60 (m, 3H), 3.41 – 3.36 (m, 2H), 2.50 (t, *J* = 6.6 Hz, 2H), 1.44 (s, 9H) ppm.
^{13}C-NMR (101 MHz, $CDCl_3$): δ = 171.1, 80.7, 70.8, 70.8, 70.8, 70.7, 70.6, 70.5, 70.2, 67.0, 50.8, 36.4, 28.2 ppm.
IR (neat): 2977, 2870, 2101, 1727, 1456, 1393, 1367, 1281, 1251, 1156, 1115, 1000, 943, 848 cm^{-1}.
HRMS (ESI): *m/z* calcd. for $C_{15}H_{33}N_4O_6$ $[M+NH_4]^+$ 365.2395, found 365.2393.

Synthesis of SI-26

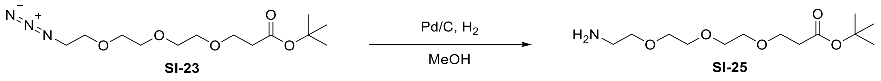

SI-23 (458 mg, 1.51 mmol, 1.00 equiv) in anhydrous MeOH (5.0 mL) was added to 10% Pd/C (120 mg, 1.13 mmol, 0.747 equiv). The reaction mixture was cautiously saturated by hydrogen at atmospheric pressure and stirred for 24 h at r.t. Subsequent filtration over celite followed by washing with EtOAc gave crude product **SI-25** which was used in the next step without further purification.

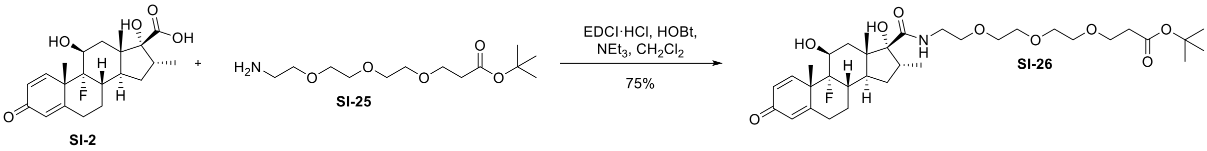

SI-2 (50 mg, 0.13 mmol, 1.0 equiv), **SI-25** (36.6 mg, 132 µmol, 1.00 equiv), EDCI·HCl (30.4 mg, 159 µmol, 1.20 equiv) and HOBt (25.4 mg, 159 µmol, 1.20 equiv) were dissolved in anhydrous CH_2Cl_2 (1.3 mL) and NEt_3 (55 µL, 0.40 mmol, 3.0 equiv) was added. The resulting reaction mixture was stirred overnight after which it was concentrated *in vacuo.* Column chromatography (eluent: grading from 80% to 90% EtOAc/hexanes) followed by preparative TLC (3% MeOH/EtOAc) yielded **SI-26** (63.6 mg, 75% yield) as a yellowish foam.

^{1}H-NMR (400 MHz, CD_3OD): δ = 7.42 (d, *J* = 10.1 Hz, 1H), 6.29 (dd, *J* = 10.1, 1.9 Hz, 1H), 6.08 (s, 1H), 4.32 – 4.17 (m, 1H), 3.69 (t, *J* = 6.2 Hz, 2H), 3.67 – 3.58 (m, 8H), 3.56 (td, *J* = 5.2, 4.4, 1.9 Hz, 2H), 3.51 – 3.43 (m, 1H), 3.39 – 3.33 (m, 1H), 3.18 – 3.07 (m, 1H), 2.72 (td, *J* = 13.2, 12.6, 5.5 Hz, 1H), 2.48 (t, *J* = 6.2 Hz, 2H), 2.39 (dd, *J* = 14.8, 3.9 Hz, 2H), 2.20 (td, *J* = 12.1, 10.6, 7.6 Hz, 2H), 1.89 (dd, *J* = 12.1, 5.5 Hz, 1H), 1.75 (q, *J* = 12.1, 11.6 Hz, 1H), 1.60 (s, 3H), 1.57 – 1.48 (m, 2H), 1.45 (s, 9H), 1.21 (dt, *J* = 8.2, 4.1 Hz, 1H), 1.08 (s, 3H), 0.90 (d, *J* = 7.3 Hz, 3H) ppm.
^{13}C-NMR (101 MHz, CD_3OD): δ = 189.0, 175.7, 172.8, 171.1, 156.1, 129.8, 125.1, 103.5, 101.8, 88.2, 81.8, 73.1 (d, *J* = 37.6 Hz), 71.6, 71.5, 71.4, 71.1, 70.7, 67.9, 50.3 (d, *J* = 22.8 Hz), 44.8, 40.0, 37.2, 36.7, 36.2, 35.8 (d, *J* = 19.4 Hz), 33.5, 32.3, 28.8, 28.4, 23.6 (d, *J* = 5.7 Hz), 17.7, 15.2 ppm.
^{19}F NMR (376 MHz, CD_3OD): δ = -165.87 ppm.
IR (neat): 3422, 2935, 2872, 1727, 1662, 1623, 1522, 1453, 1367, 1293, 1244, 1158, 1124, 1035, 893 cm^{-1}.
HRMS (ESI): *m/z* calcd. for $C_{34}H_{53}FNO_9$ $[M+H]^+$ 638.3699, found 638.3701.
$[\alpha]^{25}_D$ = 43.4 (c = 1.0, CH_3OH).

Synthesis of SI-28

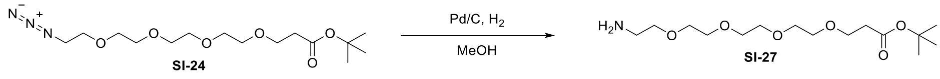

SI-24 (612 mg, 1.76 mmol, 1.00 equiv) in anhydrous MeOH (5.2 mL) was added to 10% Pd/C (100 mg, 0.940 µmol, 0.533 equiv). The reaction mixture was cautiously saturated by hydrogen at atmospheric pressure and stirred for 24 h at r.t. Subsequent filtration over celite followed by washing with EtOAc gave crude product **SI-27** which was used in the next step without further purification.

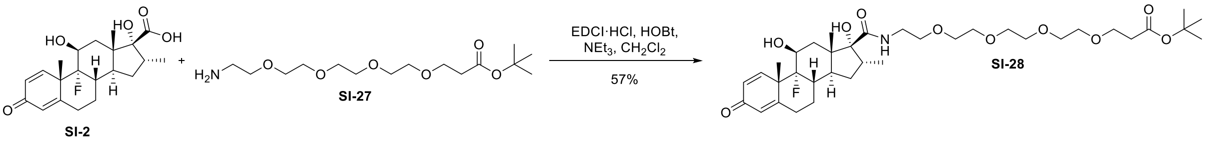

SI-2 (50 mg, 0.13 mmol, 1.0 equiv), **SI-27** (46.7 mg, 145 µmol, 1.10 equiv), EDCI·HCl (30.4 mg, 159 µmol, 1.20 equiv) and HOBt (25.4 mg, 159 µmol, 1.20 equiv) were dissolved in anhydrous CH_2Cl_2 (1.3 mL) and NEt_3 (55.2 µL, 0.40 mmol, 3.0 equiv) was added. The resulting reaction mixture was stirred overnight after which it was concentrated *in vacuo.* Column chromatography (eluent: EtOAc) followed by preparative TLC (3% MeOH/EtOAc) yielded **SI-28** (51.3 mg, 57% yield) as a yellowish foam.

^{1}H-NMR (500 MHz, CD_3OD): δ = 7.42 (d, *J* = 10.1 Hz, 1H), 6.29 (dd, *J* = 10.1, 1.8 Hz, 1H), 6.08 (s, 1H), 4.27 – 4.21 (m, 1H), 3.69 (t, *J* = 6.2 Hz, 2H), 3.65 (d, *J* = 5.3 Hz, 8H), 3.62 – 3.54 (m, 6H), 3.47 (dt, *J* = 13.8, 5.2 Hz, 1H), 3.39 – 3.32 (m, 1H), 3.19 – 3.07 (m, 1H), 2.72 (td, *J* = 13.8, 13.3, 5.9 Hz, 1H), 2.47 (t, *J* = 6.2 Hz, 2H), 2.40 (td, *J* = 13.8, 13.0, 5.5 Hz, 2H), 2.25 – 2.17 (m, 2H), 1.92 – 1.84 (m, 1H), 1.75 (q, *J* = 11.5 Hz, 1H), 1.60 (s, 3H), 1.56 – 1.47 (m, 2H), 1.45 (s, 9H), 1.21 (dd, *J* = 8.3, 4.1 Hz, 1H), 1.08 (s, 3H), 0.90 (d, *J* = 7.3 Hz, 3H) ppm.
^{13}C-NMR (125 MHz, CD_3OD): δ = 189.0, 175.7, 172.8, 171.2, 156.1, 129.8, 125.1, 103.4, 102.0, 88.2, 81.8, 73.1 (d, *J* = 37.6 Hz), 71.6, 71.6, 71.5, 71.0, 71.4, 71.1, 70.7, 67.9, 50.3 (d, *J* = 22.7 Hz), 44.8, 40.0, 37.2, 36.7, 36.2, 35.8 (d, *J* = 15.2 Hz), 33.5, 32.3, 28.8, 28.4, 23.1 (d, *J* = 5.7 Hz), 17.7, 15.2 ppm.
^{19}F NMR (471 MHz, CD_3OD): δ = -165.86 ppm.
IR (neat): 3421, 2934, 2872, 1728, 1663, 1523, 1454, 1368, 1293, 1245, 1124, 1035, 893 cm^{-1}.
HRMS (ESI): *m/z* calcd. for $C_{36}H_{56}FNNaO_{10}$ $[M+Na]^+$ 704.3780, found 704.3771.
$[\alpha]^{25}_D$ = 36.0 (c = 0.5, CH_3OH).

Synthesis of KH-95

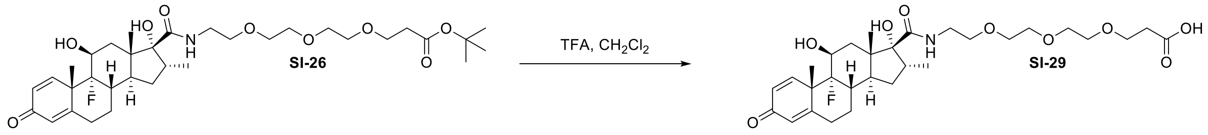

SI-26 (51.8 mg, 81.2 μmol, 1.00 equiv) was dissolved in anhydrous CH_2Cl_2 (0.38 mL) and TFA (0.38 mL) was added. The resulting reaction mixture was stirred at r.t. for 1.5 h after which it was diluted with toluene and concentrated *in vacuo*. Crude product **SI-29** was used in the next step without further purification.

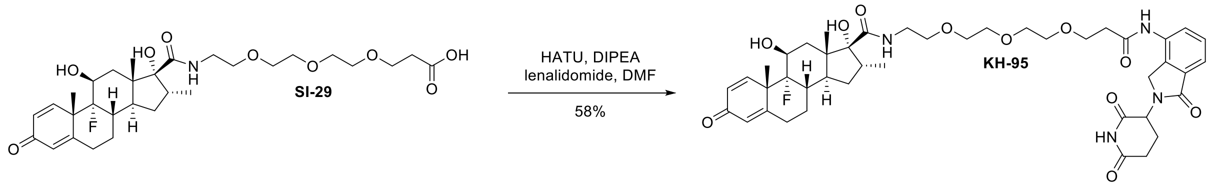

SI-29 (47.2 mg, 81.1 µmol, 1.00 equiv), lenalidomide (21.0 mg, 81.1 µmol, 1.00 equiv) and HATU (34.0 mg, 89.3 µmol, 1.10 equiv) were dissolved in anhydrous DMF (0.9 mL) and DIPEA (42.1 µL, 243 µmol, 3.00 equiv) was added. The resulting reaction mixture was stirred overnight after which it was concentrated *in vacuo.* Column chromatography (eluent: grading from 5% to 10% MeOH/EtOAc) followed by preparative TLC (15% MeOH/EtOAc) yielded **KH-95** (38.5 mg, 58% yield) as a colorless oil.

^{1}H-NMR (400 MHz, CD_3OD): δ = 7.76 (dt, *J* = 7.9, 1.0 Hz, 1H), 7.65 (dd, *J* = 7.6, 1.0 Hz, 1H), 7.52 (t, *J* = 7.7 Hz, 1H), 7.41 (dd, *J* = 10.1, 1.8 Hz, 1H), 6.28 (dt, *J* = 10.1, 1.9 Hz, 1H), 6.07 (q, *J* = 1.6 Hz, 1H), 5.17 (dd, *J* = 13.3, 5.1 Hz, 1H), 4.56 – 4.40 (m, 2H), 4.28 – 4.16 (m, 1H), 3.83 (t, *J* = 6.0 Hz, 2H), 3.70 – 3.64 (m, 4H), 3.62 (dd, *J* = 5.2, 4.1 Hz, 2H), 3.58 – 3.52 (m, 2H), 3.51 – 3.45 (m, 2H), 3.44 – 3.36 (m, 1H), 3.30 – 3.21 (m, 1H), 3.17 – 3.03 (m, 1H), 2.97 – 2.84 (m, 1H), 2.82 – 2.73 (m, 2H), 2.69 (t, *J* = 6.0 Hz, 2H), 2.57 – 2.28 (m, 3H), 2.28 – 2.08 (m, 3H), 1.92 – 1.80 (m, 1H), 1.72 (q, *J* = 11.5 Hz, 1H), 1.57 (s, 3H), 1.55 – 1.39 (m, 2H), 1.24 – 1.12 (m, 1H), 1.06 (s, 3H), 0.88 (d, *J* = 7.3 Hz, 3H) ppm.
^{13}C-NMR (101 MHz, CD_3OD): δ = 189.0, 175.6, 174.7, 172.5, 172.1 (d, *J* = 1.2 Hz), 171.2, 171.1, 156.1, 136.3, 134.5, 133.9, 130.1, 129.8, 127.8, 125.1, 121.5, 103.6, 101.8, 88.2, 73.1 (d, *J* = 37.5 Hz), 71.5, 71.4, 71.3, 71.1, 70.7, 68.1, 53.6, 50.3 (d, *J* = 22.7 Hz), 44.8, 39.9, 37.9, 36.7, 36.1, 35.7 (d, *J* = 19.3 Hz), 33.4, 32.4, 32.2, 28.8, 24.2, 23.6 (d, *J* = 5.7 Hz), 17.7, 15.3 ppm.
^{19}F NMR (376 MHz, CD_3OD): δ = -161.92 ppm.
^{1}H-NMR (600 MHz, $(CD_3)_2CO$): δ = 9.82 (s, 1H), 9.17 (s, 1H), 7.95 (dt, *J* = 7.7, 1.0 Hz, 1H), 7.56 (dd, *J* = 7.6, 1.1 Hz, 1H), 7.50 (t, *J* = 7.7 Hz, 1H), 7.30 (dd, *J* = 10.3, 1.4 Hz, 1H), 7.07 – 7.02 (m, 1H), 6.21 – 6.17 (m, 1H), 6.01 – 5.98 (m, 1H), 5.24 – 5.19 (m, 1H), 4.55 – 4.43 (m, 2H), 4.35 – 4.29 (m, 2H), 3.87 – 3.81 (m, 2H), 3.77 – 3.76 (m, 1H), 3.68 – 3.61 (m, 4H), 3.61 – 3.56 (m, 2H), 3.53 – 3.46 (m, 2H), 3.45

– 3.40 (m, 2H), 3.40 – 3.35 (m, 1H), 3.25 – 3.18 (m, 1H), 3.18 – 3.12 (m, 1H), 3.04 – 2.96 (m, 1H), 2.81 – 2.75 (m, 1H), 2.75 – 2.69 (m, 1H), 2.67 (t, *J* = 6.0 Hz, 2H), 2.55 – 2.40 (m, 2H), 2.35 (dt, *J* = 13.9, 3.3 Hz, 1H), 2.26 – 2.21 (m, 1H), 2.21 – 2.16 (m, 2H), 1.86 (dt, *J* = 13.1, 5.5 Hz, 1H), 1.76 – 1.68 (m, 1H), 1.60 (s, 3H), 1.55 (dt, *J* = 13.6, 2.4 Hz, 1H), 1.52 – 1.42 (m, 1H), 1.20 – 1.14 (m, 1H), 1.10 (dd, *J* = 1.5, 0.7 Hz, 3H), 0.90 (d, *J* = 7.3 Hz, 3H) ppm.

IR (neat): 3408, 2935, 2873, 1662, 1604, 1534, 1434, 1353, 1296, 1239, 1131, 894, 802 cm^{-1}.

HRMS (ESI): *m/z* calcd. for $C_{43}H_{55}FN_4NaO_{11}$ $[M+Na]^+$ 845.3744, found 845.3745.

$[\alpha]^{25}_D$ = 32.8 (c = 0.5, CH_3OH).

Synthesis of KH-99

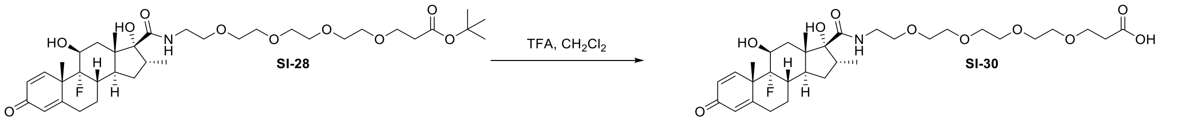

SI-28 (47.9 mg, 70.3 µmol, 1.00 equiv) was dissolved in anhydrous CH_2Cl_2 (0.35 mL) and TFA (0.35 mL) was added. The resulting reaction mixture was stirred at r.t. for 1.5 h after which it was diluted with toluene and concentrated *in vacuo*. Crude product **SI-30** was used in the next step without further purification.

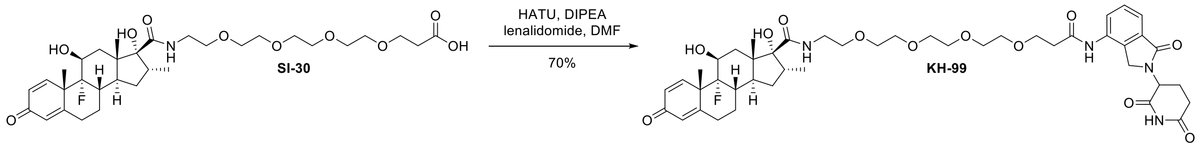

SI-30 (44.0 mg, 70.3 µmol, 1.00 equiv), lenalidomide (18.2 mg, 70.3 µmol, 1.00 equiv) and HATU (29.5 mg, 77.4 µmol, 1.10 equiv) were dissolved in anhydrous DMF (0.65 mL) and DIPEA (36.5 µL, 211 µmol, 3.00 equiv) was added. The resulting reaction mixture was stirred overnight after which it was concentrated *in vacuo.* Column chromatography (eluent: grading from 5% to 10% MeOH/EtOAc) followed by preparative TLC (15% MeOH/EtOAc) yielded **KH-99** (42.9 mg, 70% yield) as a colorless oil.

^{1}H-NMR (400 MHz, CD_3OD): δ = 7.76 (d, *J* = 7.9 Hz, 1H), 7.66 (dd, *J* = 7.6, 1.0 Hz, 1H), 7.53 (t, *J* = 7.7 Hz, 1H), 7.41 (dd, *J* = 10.1, 1.5 Hz, 1H), 6.28 (dt, *J* = 10.1, 1.6 Hz, 1H), 6.07 (t, *J* = 1.7 Hz, 1H), 5.23 – 5.09 (m, 1H), 4.57 – 4.42 (m, 2H), 4.29 – 4.17 (m, 1H), 3.83 (t, *J* = 5.8 Hz, 2H), 3.70 – 3.64 (m, 4H), 3.64 – 3.59 (m, 2H), 3.59 – 3.52 (m, 6H), 3.51 – 3.45 (m, 2H), 3.45 – 3.37 (m, 1H), 3.30 – 3.22 (m, 1H), 3.16 – 3.05 (m, 1H), 2.97 – 2.85 (m, 1H), 2.83 – 2.74 (m, 2H), 2.71 (t, *J* = 5.8 Hz, 2H), 2.53 – 2.32 (m, 3H), 2.24 – 2.10 (m, 3H), 1.91 – 1.81 (m, 1H), 1.73 (dt, *J* = 13.8, 11.4 Hz, 1H), 1.58 (s, 3H), 1.53 – 1.41 (m, 2H), 1.25 – 1.13 (m, 1H), 1.07 (s, 3H), 0.94 – 0.85 (m, 3H) ppm.
^{13}C-NMR (101 MHz, CD_3OD): δ = 189.0, 175.7, 174.6, 172.6, 172.1 (d, *J* = 1.2 Hz), 171.2, 171.1, 156.1, 136.4, 134.5, 133.9, 130.1, 129.8, 127.8, 125.1, 121.5, 103.6, 101.8, 88.2, 73.1 (d, *J* = 37.6 Hz), 71.4, 71.3, 71.3, 71.0, 70.9, 68.1, 53.6, 50.3 (d, *J* = 22.7 Hz), 44.8, 39.9, 37.8, 36.8, 36.2, 35.8 (d, *J* = 19.2 Hz), 33.4, 32.4, 32.2, 28.8, 24.2, 23.6, 23.6, 17.8, 15.2 ppm.
^{19}F NMR (376 MHz, CD_3OD): δ = -165.77 ppm.
^{1}H-NMR (600 MHz, $(CD_3)_2CO$): δ = 9.82 (s, 1H), 9.14 (s, 1H), 7.97 (dd, *J* = 7.9, 1.1 Hz, 1H), 7.56 (dd, *J* = 7.6, 1.1 Hz, 1H), 7.50 (t, *J* = 7.7 Hz, 1H), 7.31 (dd, *J* = 10.1, 1.1 Hz, 1H), 7.06 (d, *J* = 5.5 Hz, 1H), 6.19 (dt, *J* = 10.1, 1.7 Hz, 1H), 5.99 (t, *J* = 1.8 Hz, 1H), 5.25 – 5.18 (m, 1H), 4.56 – 4.43 (m, 2H), 4.35 – 4.28 (m, 2H), 3.86 – 3.79 (m, 2H), 3.76 (s, 1H), 3.68 – 3.62 (m, 4H), 3.62 – 3.57 (m, 2H), 3.56 – 3.48 (m, 6H), 3.48 – 3.45 (m, 2H), 3.45 – 3.40 (m, 1H), 3.29 – 3.22 (m, 1H), 3.20 – 3.13 (m, 1H), 3.04 – 2.95 (m, 1H), 2.81 – 2.75 (m, 1H), 2.75 – 2.68 (m, 1H), 2.66 (t, *J* = 6.0 Hz, 2H), 2.58 – 2.40 (m, 2H), 2.38 –

2.32 (m, 1H), 2.27 – 2.16 (m, 3H), 1.86 (dt, *J* = 11.8, 5.4 Hz, 1H), 1.79 – 1.68 (m, 1H), 1.60 (s, 3H), 1.57 (dt, *J* = 14.2, 1.7 Hz, 1H), 1.51 – 1.43 (m, 1H), 1.20 – 1.15 (m, 1H), 1.10 (s, 3H), 0.90 (d, *J* = 7.3 Hz, 3H) ppm.

IR (neat): 3417, 2933, 2873, 1678, 1604, 1533, 1434, 1355, 1296, 1235, 1205, 1130, 893, 802 cm^{-1}.

HRMS (ESI): *m/z* calcd. for $C_{45}H_{59}FN_4NaO_{12}$ $[M+Na]^+$ 889.4006, found 889.4006.

$[\alpha]^{25}_D$ = 26.0 (c = 1.0, CH_3OH).
