# Supplementary material for: Harnessing PROTAC technology to combat stress hormone receptor activation
Source: Nat Commun. 2023 Dec 9;14:8177. doi: 10.1038/s41467-023-44031-2 (PMC10710461; doi:10.1038/s41467-023-44031-2)
Supplement: Supplementary file 3 — Description of Additional Supplementary Files [file 41467_2023_44031_MOESM3_ESM.pdf]

Description of Additional Supplementary Files

File name: Supplementary Data 1

Description: The Supplementary Data 1 provides NMR Spectra of PROTAC KH-95, PROTAC KH-99, PROTAC KH-102 and PROTAC KH-103 and their synthetic intermediates.

File name: Supplementary Data 2

Description: The Supplementary Data 2 provides the 5001st and the last MD frames
114 of PROTAC KH-95, PROTAC KH-99, PROTAC KH-102 and PROTAC KH-103.
