# Supplementary material for: Harnessing PROTAC technology to combat stress hormone receptor activation
Source: Nat Commun. 2023 Dec 9;14:8177. doi: 10.1038/s41467-023-44031-2 (PMC10710461; doi:10.1038/s41467-023-44031-2)
Supplement: Supplementary file 4 — Supplementary Data 1 [file 41467_2023_44031_MOESM4_ESM.docx]

**Supplementary Data 1**

Harnessing PROTAC technology to combat stress hormone receptor activation

Mahshid Gazorpak*^1,2^, Karina M. Hugentobler*^3^, Dominique Paul^4^, Pierre-Luc Germain^1,4,5^, Miriam Kretschmer^1,2^, Iryna Ivanova^1^, Selina Frei^1^, Kei Mathis^1^, Remo Rudolf^1^, Sergio Mompart Barrenechea^1^, Vincent Fischer^1,2^, Xiaohan Xue^6^, Aleksandra L. Ptaszek^7^, Julian Holzinger^7^, Mattia Privitera^8^, Andreas Hierlemann^6^, Onno C. Meijer^9^, Robert Konrat^10^, Erick M. Carreira^3^, Johannes Bohacek^2,8^, Katharina Gapp^#1,2^

^1^Laboratory of Epigenetics and Neuroendocrinology, Institute for Neuroscience, Department of Health Science and Technology, ETH Zurich, 8057 Zurich, Switzerland

^2^Neuroscience Center Zurich, ETH Zurich and University of Zurich, Switzerland

^3^Laboratory of Organic Chemistry, Department of Chemistry and Applied Biosciences, ETH Zurich, 8093, Zurich, Switzerland

^4^Lab of Statistical Bioinformatics, University of Zürich, Switzerland,

^5^Computational Neurogenomics, Institute for Neuroscience, Department of Health Science and Technology, ETH Zurich, 8057 Zurich, Switzerland

^6^Bio Engineering Laboratory, Department of Biosystems Science and Engineering, ETH Zurich, 4056 Basel, Switzerland

^7^Christian Doppler Laboratory for High-Content Structural Biology and Biotechnology, Max Perutz Laboratories, Department of Structural and Computational Biology, University of Vienna, Campus Vienna Biocenter 5, 1030 Vienna, Austria

^8^Laboratory of Molecular and Behavioral Neuroscience, Institute for Neuroscience, Department of Health Science and Technology, ETH Zurich, 8057 Zurich, Switzerland

^9^Department of Medicine, Division of Endocrinology, Leiden University Medical Center, 2300 RA Leiden, the Netherlands

^10^Department of Structural and Computational Biology, University of Vienna, Campus Vienna Biocenter 5, 1030 Vienna, Austria

* These authors contributed equally

#Corresponding: Prof. Katharina Gapp (katharina.gapp@hest.ethz.ch)

^1^H NMR (500 MHz, CD_3_OD) of compound **SI-2**:

^13^C NMR (126 MHz, CD_3_OD) of compound **SI-2**:

^19^F NMR (376 MHz, CD_3_OD) of compound **SI-2**:

^1^H NMR (400 MHz, CDCl_3_) of compound **SI-4**:

^13^C NMR (101 MHz, CDCl_3_) of compound **SI-4**:

^1^H NMR (400 MHz, CDCl_3_) of compound **SI-6**:

^13^C NMR (101 MHz, CDCl_3_) of compound **SI-6**:

^1^H NMR (400 MHz, CDCl_3_) of compound **SI-7**:

^13^C NMR (101 MHz, CDCl_3_) of compound **SI-7**:

^1^H NMR (400 MHz, CDCl_3_) of compound **SI-8**:

^13^C NMR (101 MHz, CDCl_3_) of compound **SI-8**:

^1^H NMR (400 MHz, CDCl_3_) of compound **SI-9**:

^13^C NMR (101 MHz, CDCl_3_) of compound **SI-9**:

^1^H NMR (400 MHz, CDCl_3_) of compound **SI-10**:

^13^C NMR (101 MHz, CDCl_3_) of compound **SI-10**:

^1^H NMR (500 MHz, CD_3_OD) of compound **SI-12**:

^13^C NMR (126 MHz, CD_3_OD) of compound **SI-12**: ^19^F NMR (471 MHz, CD_3_OD) of compound **SI-12**:

^1^H NMR (400 MHz, CD_3_OD) of compound **SI-14**:

^13^C NMR (101 MHz, CD_3_OD) of compound **SI-14**:

^19^F NMR (376 MHz, CD_3_OD) of compound **SI-14**:

^1^H NMR (400 MHz, CD_3_OD) of compound **KH-103**:

^13^C NMR (101 MHz, CD_3_OD) of compound **KH-103**:

^19^F NMR (376 MHz, CD_3_OD) of compound **KH-103**:

^1^H NMR (600 MHz, (CD_3_)_2_CO) of compound **KH-103**:

ROESY (600 MHz, (CD_3_)_2_CO) of compound **KH-103**:

^1^H NMR (400 MHz, CD_3_OD) of compound **KH-102**:

^13^C NMR (101 MHz, CD_3_OD) of compound **KH-102**:

^19^F NMR (376 MHz, CD_3_OD) of compound **KH-102**:

^1^H NMR (600 MHz, (CD_3_)_2_CO) of compound **KH-102**:

ROESY (600 MHz, (CD_3_)_2_CO) of compound **KH-102**:

^1^H NMR (400 MHz, CDCl_3_) of compound **SI-19**:

^13^C NMR (101 MHz, CDCl_3_) of compound **SI-19**:

^1^H NMR (400 MHz, CDCl_3_) of compound **SI-22**:

^13^C NMR (101 MHz, CDCl_3_) of compound **SI-22**:

^1^H NMR (400 MHz, CDCl_3_) of compound **SI-23**:

^13^C NMR (101 MHz, CDCl_3_) of compound **SI-23**:

^1^H NMR (400 MHz, CDCl_3_) of compound **SI-24**:

^13^C NMR (101 MHz, CDCl_3_) of compound **SI-24**:

^1^H NMR (400 MHz, CD_3_OD) of compound **SI-26**:

^13^C NMR (101 MHz, CD_3_OD) of compound **SI-26**:

^19^F NMR (376 MHz, CD_3_OD) of compound **SI-26**:

^1^H NMR (400 MHz, CD_3_OD) of compound **SI-28**:

^13^C NMR (101 MHz, CD_3_OD) of compound **SI-28**:

^19^F NMR (376 MHz, CD_3_OD) of compound **SI-28**:

^1^H NMR (400 MHz, CD_3_OD) of compound **KH-95**:

^13^C NMR (101 MHz, CD_3_OD) of compound **KH-95**:

^19^F NMR (376 MHz, CD_3_OD) of compound **KH-95**:

^1^H NMR (600 MHz, (CD_3_)_2_CO) of compound **KH-95**:

ROESY (600 MHz, (CD_3_)_2_CO) of compound **KH-95**:

^1^H NMR (400 MHz, CD_3_OD) of compound **KH-99**:

^13^C NMR (101 MHz, CD_3_OD) of compound **KH-99**:

^19^F NMR (376 MHz, CD_3_OD) of compound **KH-99**: ^1^H NMR (600 MHz, (CD_3_)_2_CO) of compound **KH-99**:

ROESY (600 MHz, (CD_3_)_2_CO) of compound **KH-99**:
