# Supplementary material for: Harnessing PROTAC technology to combat stress hormone receptor activation
Source: Nat Commun. 2023 Dec 9;14:8177. doi: 10.1038/s41467-023-44031-2 (PMC10710461; doi:10.1038/s41467-023-44031-2)
Supplement: Supplementary file 5 — Supplementary Data 2 [file 41467_2023_44031_MOESM5_ESM.docx]

**Supplementary Data 2**

**Harnessing PROTAC technology to combat stress hormone receptor activation**

Mahshid Gazorpak*^1,2^, Karina M. Hugentobler*^3^, Dominique Paul^4^, Pierre-Luc Germain^1,4,5^, Miriam Kretschmer^1,2^, Iryna Ivanova^1^, Selina Frei^1^, Kei Mathis^1^, Remo Rudolf^1^, Sergio Mompart Barrenechea^1^, Vincent Fischer^1,2^, Xiaohan Xue^6^, Aleksandra L. Ptaszek^7^, Julian Holzinger^7^, Mattia Privitera^8^, Andreas Hierlemann^6^, Onno C. Meijer^9^, Robert Konrat^10^, Erick M. Carreira^3^, Johannes Bohacek^2,8^, Katharina Gapp^#1,2^

^1^Laboratory of Epigenetics and Neuroendocrinology, Institute for Neuroscience, Department of Health Science and Technology, ETH Zurich, 8057 Zurich, Switzerland

^2^Neuroscience Center Zurich, ETH Zurich and University of Zurich, Switzerland

^3^Laboratory of Organic Chemistry, Department of Chemistry and Applied Biosciences, ETH Zurich, Zurich, Switzerland

^4^Lab of Statistical Bioinformatics, University of Zürich, Switzerland,

^5^Computational Neurogenomics, Institute for Neuroscience, Department of Health Science and Technology, ETH Zurich, 8057 Zurich, Switzerland

^6^Bio Engineering Laboratory, Department of Biosystems Science and Engineering, ETH Zurich, 4056 Basel, Switzerland

^7^Christian Doppler Laboratory for High-Content Structural Biology and Biotechnology, Max Perutz Laboratories,  Department of Structural and Computational Biology, University of Vienna, Campus Vienna Biocenter 5, 1030 Vienna, Austria

^8^ Laboratory of Molecular and Behavioral Neuroscience, Institute for Neuroscience, Department of Health Science and Technology, ETH Zurich, 8057 Zurich, Switzerland

^9^ Department of Medicine, Division of Endocrinology, Leiden University Medical Center, 2300 RA Leiden, the Netherlands

^10^ Department of Structural and Computational Biology, University of Vienna, Campus Vienna Biocenter 5, 1030 Vienna, Austria

* These authors contributed equally

#Corresponding: Prof. Katharina Gapp (katharina.gapp@hest.ethz.ch)

KH-95 the first, the 5001st and the last MD frames

114

energy: -181.477665134693 gnorm: 0.000733370379 xtb: 6.4.1 (afa7bdf)

C 3.47100000000000 8.58690000000000 10.72280000000000

C 2.33710000000000 8.11570000000000 11.23760000000000

C 2.32840000000000 7.14000000000000 12.32780000000000

C 3.63400000000000 6.78220000000000 12.85720000000000

C 4.78800000000000 7.24520000000000 12.35780000000000

C 4.83380000000000 8.17980000000000 11.18050000000000

C 5.54770000000000 7.36960000000000 10.04120000000000

C 6.11460000000000 6.79310000000000 12.87140000000000

C 6.89140000000000 6.10180000000000 11.74450000000000

C 6.96080000000000 6.95130000000000 10.47260000000000

C 6.26460000000000 7.26080000000000 7.56490000000000

C 5.45070000000000 8.01710000000000 8.63740000000000

C 7.59140000000000 6.15250000000000 9.33690000000000

C 7.68610000000000 6.94660000000000 8.02580000000000

C 8.44140000000000 5.89890000000000 7.15010000000000

C 9.00310000000000 5.61160000000000 9.54840000000000

C 9.52040000000000 5.33770000000000 8.11960000000000

O 1.28130000000000 6.66350000000000 12.77040000000000

H 6.93370000000000 5.30000000000000 9.13100000000000

C 8.50210000000000 8.23430000000000 8.12790000000000

C 9.09550000000000 6.53210000000000 5.91150000000000

C 9.83490000000000 3.86340000000000 7.88800000000000

O 5.78430000000000 9.37860000000000 8.69900000000000

C 5.56880000000000 9.47750000000000 11.56890000000000

F 4.78950000000000 6.18840000000000 9.90150000000000

H 7.54620000000000 7.85300000000000 10.65290000000000

O 7.49390000000000 4.93440000000000 6.72840000000000

O 10.27040000000000 6.87480000000000 5.86870000000000

N 8.25990000000000 6.63450000000000 4.86810000000000

C 8.64390000000000 7.26980000000000 3.62780000000000

C 8.73290000000000 8.78980000000000 3.76740000000000

O 7.44250000000000 9.31150000000000 4.01360000000000

C 7.45700000000000 10.70460000000000 4.22150000000000

C 6.01780000000000 11.19400000000000 4.33840000000000

O 6.05160000000000 12.57360000000000 4.63550000000000

C 4.77320000000000 13.15760000000000 4.74380000000000

C 4.97850000000000 14.63220000000000 5.07740000000000

O 3.78680000000000 15.34400000000000 4.82360000000000

C 3.94920000000000 16.74320000000000 5.00050000000000

C 2.70990000000000 17.47880000000000 4.49440000000000

C 1.49980000000000 17.34730000000000 5.40490000000000

N 1.17920000000000 16.05470000000000 5.66930000000000

O 0.89150000000000 18.31970000000000 5.81520000000000

C 0.09750000000000 15.55740000000000 6.39730000000000

C -0.76090000000000 16.36860000000000 7.14340000000000

C -0.16800000000000 14.19130000000000 6.37940000000000

C -1.26220000000000 13.67460000000000 7.06920000000000

C -2.11440000000000 14.48150000000000 7.80440000000000

C -1.84060000000000 15.83840000000000 7.83230000000000

C 0.54470000000000 13.07020000000000 5.68270000000000

N -0.23170000000000 11.91180000000000 6.08290000000000

C -1.32430000000000 12.22200000000000 6.83680000000000

C -0.03260000000000 10.56790000000000 5.56210000000000

C 1.08920000000000 10.57300000000000 4.53800000000000

N 2.33870000000000 10.27500000000000 5.02690000000000

C 2.71980000000000 10.00560000000000 6.32590000000000

C 1.61460000000000 9.92060000000000 7.33390000000000

C 0.28080000000000 9.57520000000000 6.68500000000000

O 0.93410000000000 10.83150000000000 3.36780000000000

O 3.89670000000000 9.87320000000000 6.58470000000000

O -2.15940000000000 11.41720000000000 7.21890000000000

H 3.45640000000000 9.33790000000000 9.94620000000000

H 1.36850000000000 8.44110000000000 10.88550000000000

H 3.62990000000000 6.09060000000000 13.68870000000000

H 6.68520000000000 7.64780000000000 13.23740000000000

H 5.97670000000000 6.09830000000000 13.69970000000000

H 7.90150000000000 5.87880000000000 12.08500000000000

H 6.39400000000000 5.15950000000000 11.50560000000000

H 6.27260000000000 7.88140000000000 6.66570000000000

H 5.74840000000000 6.32620000000000 7.34370000000000

H 4.39140000000000 7.91540000000000 8.34880000000000

H 9.62690000000000 6.34610000000000 10.05650000000000

H 8.99860000000000 4.69890000000000 10.14080000000000

H 10.43280000000000 5.90920000000000 7.92760000000000

H 8.01380000000000 8.94400000000000 8.78690000000000

H 9.50990000000000 8.04380000000000 8.48230000000000

H 8.57760000000000 8.69330000000000 7.14390000000000

H 8.96800000000000 3.24010000000000 8.09860000000000

H 10.16890000000000 3.69680000000000 6.86470000000000

H 10.63940000000000 3.56000000000000 8.55310000000000

H 5.43890000000000 9.79650000000000 7.89660000000000

H 5.01310000000000 9.95510000000000 12.37280000000000

H 5.61580000000000 10.14620000000000 10.71690000000000

H 6.57800000000000 9.28340000000000 11.91130000000000

H 7.96170000000000 4.16230000000000 6.38740000000000

H 7.29030000000000 6.39810000000000 5.01670000000000

H 9.63240000000000 6.89930000000000 3.34410000000000

H 7.91070000000000 7.01160000000000 2.86550000000000

H 9.41100000000000 9.03890000000000 4.59600000000000

H 9.14460000000000 9.22050000000000 2.84340000000000

H 7.95270000000000 11.21520000000000 3.38360000000000

H 8.00320000000000 10.95520000000000 5.14260000000000

H 5.50970000000000 10.63570000000000 5.13590000000000

H 5.49450000000000 11.01740000000000 3.38690000000000

H 4.18870000000000 12.65750000000000 5.52950000000000

H 4.22560000000000 13.07730000000000 3.79240000000000

H 5.28120000000000 14.74750000000000 6.12640000000000

H 5.79070000000000 15.01220000000000 4.44300000000000

H 4.82340000000000 17.08030000000000 4.42770000000000

H 4.12720000000000 16.96760000000000 6.06170000000000

H 2.44670000000000 17.08560000000000 3.51040000000000

H 2.93650000000000 18.54030000000000 4.39820000000000

H 1.81400000000000 15.36570000000000 5.27740000000000

H -0.57850000000000 17.42980000000000 7.17060000000000

H -2.95520000000000 14.05390000000000 8.32610000000000

H -2.47720000000000 16.50280000000000 8.39490000000000

H 1.58810000000000 12.97830000000000 6.00730000000000

H 0.52710000000000 13.19180000000000 4.59310000000000

H -0.95730000000000 10.26390000000000 5.05170000000000

H 3.08920000000000 10.27430000000000 4.33810000000000

H 1.54590000000000 10.88790000000000 7.84160000000000

H 1.88680000000000 9.18010000000000 8.08810000000000

H -0.52160000000000 9.61420000000000 7.42080000000000

H 0.31230000000000 8.56800000000000 6.26710000000000

114

energy: -181.384932342205 gnorm: 0.155160544180 xtb: 6.4.1 (afa7bdf)

C 3.25442566436036 8.85016081612925 13.02964568467240

C 2.81387362757273 8.03941607754643 13.98953349507525

C 3.75245603891489 7.43253266033977 14.93365099098257

C 5.05672231463582 8.07406989501845 14.94765378516351

C 5.46522595127710 8.94991405072378 14.01947965243656

C 4.62550709964636 9.42036937897074 12.86399092328727

C 5.34477614934098 8.76267322169519 11.63347492730636

C 6.85030366722073 9.50594022308747 14.04049393384278

C 7.57701358111396 8.89503624301537 12.83637326244802

C 6.81243400145171 9.20584298653785 11.54679446093562

C 5.38169666598617 8.22586123816518 9.16155469865420

C 4.58728154893979 8.91688730928295 10.29118595310874

C 7.38158971450136 8.33157115910956 10.43447127657029

C 6.83633519202528 8.67434016627130 9.04028583383489

C 7.64092705812268 7.67965945605007 8.14683963388207

C 8.89152251842899 8.21362200718604 10.24370206003921

C 9.05737031712228 7.72762422373533 8.78754595089976

O 3.43840442501739 6.45392743997232 15.61414262159386

H 7.06050299342412 7.29617017708666 10.59746969129923

C 7.09451268493041 10.13038053419171 8.65591289939302

C 7.71412345941111 8.09808454856651 6.66951713623979

C 9.79833357112638 6.40119443439648 8.65462951215819

O 4.11657696707418 10.21107892064572 10.02235317466436

C 4.46767434345246 10.94799811103172 12.73536672051032

F 5.33878087233667 7.36727090639803 11.83946821352344

H 6.84534784025921 10.23907526017599 11.20094491896094

O 7.02179140997641 6.40656798238461 8.10492837713031

O 8.70922410584819 8.70443072357440 6.29297474634920

N 6.58305717405725 7.74374708570445 6.04299441942665

C 5.97076654051257 8.18200495642261 4.80912392115724

C 5.58299111702931 9.65005740777742 4.98873846335249

O 4.92159371376779 10.20762937507386 3.87089032196241

C 5.62574050640124 10.85730755501857 2.83829367889454

C 4.91958403884211 10.57185844782997 1.51756037474361

O 3.62460822808344 11.12434175436025 1.62006332907006

C 3.02659646662703 11.33652448458723 0.36131320597773

C 1.78367924410973 12.21830802976148 0.43545339877457

O 1.84350602407044 13.31375855434582 1.32293944151947

C 2.30680025487694 14.53250199021789 0.76128927586611

C 2.01275596280616 15.61543489952173 1.79758235787663

C 2.73360435895671 15.64017252799975 3.13564446550374

N 2.80051801315109 14.38389735235641 3.64633205754941

O 3.09991161495492 16.69578165205643 3.62087138053064

C 3.22275372060570 13.93411333916922 4.89804195879744

C 3.99861192664892 14.65264690994702 5.81090279586963

C 2.79589014845846 12.68818127644276 5.34802066151460

C 3.09955516232422 12.18358996165795 6.61026148342611

C 3.81376074174565 12.94990142872767 7.51609191504379

C 4.28820353496551 14.17517982677102 7.07960659165264

C 2.20233874107110 11.65509325678267 4.43663524002015

N 2.07592068905908 10.51515377805590 5.32511939420963

C 2.63167029493358 10.78932772352859 6.53934142072147

C 1.60468450248081 9.16949288286204 5.03516432276164

C 2.10442059074189 8.34204435582692 6.20684468451357

N 1.13075148224086 7.93579984724849 7.08775974494315

C -0.17770825862545 8.36990399104890 7.15632562417622

C -0.65370744705353 9.34279223224397 6.12111101255791

C 0.09486669165011 9.00806439999156 4.83776037472123

O 3.16791735845174 7.76912996444276 6.17537980603758

O -0.89973453356627 7.69798998037081 7.86117343448978

O 2.69898056122940 9.92906427461780 7.40335048691972

H 2.52914972324802 9.19584842222160 12.30729076338643

H 1.83039045658590 7.60640107535773 13.87418873765511

H 5.69722316646473 7.79376816019773 15.77282603736958

H 6.85334630093112 10.58779745924347 13.90039351478540

H 7.46039676097405 9.24704579935546 14.90571324073343

H 8.59657156915483 9.27553258485664 12.87754743564776

H 7.68576230708875 7.82652372095242 13.03351274112901

H 4.71262003220797 8.45894317985237 8.32982260503835

H 5.37380563754497 7.14595203578806 9.31189729212167

H 3.72009646705377 8.25627065053656 10.45648875803115

H 9.37730326186126 9.16845924855011 10.44140187719958

H 9.37188227164632 7.50204514076628 10.91220200520752

H 9.61735790017474 8.40469211731003 8.13643690487989

H 8.16871860267257 10.27882674609230 8.63499992027642

H 6.69684157401583 10.35871620131564 7.67241355240371

H 6.79474783859534 10.86651641039308 9.39947343105372

H 9.14609497108025 5.58560122588295 8.96083759280201

H 10.01032827423291 6.16340680578210 7.61301070398494

H 10.68438382950856 6.24230246631952 9.26397186620244

H 3.53066294002236 10.25045604112655 9.25232620842957

H 5.25986352426055 11.42356549443245 13.30932790043750

H 3.54227246596751 11.17634249310351 13.25185767608484

H 4.48613319692886 11.38896480810762 11.74612212639839

H 7.65505891236316 5.86646075750703 7.61655074928954

H 5.83408060829788 7.59034574100144 6.70147237259495

H 6.69731323403660 8.02668373697323 4.00731757736567

H 5.02759708536471 7.66820539467264 4.63082035638531

H 4.88106355174309 9.78069357244817 5.82460942602816

H 6.51143673316369 10.14076004948956 5.31427653475998

H 6.65616778711540 10.47445985168734 2.85072409754852

H 5.66651770226327 11.94908571108146 2.96445900041396

H 4.79588076457466 9.48559961521060 1.41455552759919

H 5.44641477095871 10.85589463604954 0.59443767242240

H 2.77870827583534 10.34734746654769 -0.04995923659148

H 3.79935583640426 11.69166114462681 -0.33739754913601

H 1.46591657373251 12.54972245564417 -0.56179970051567

H 0.94664649607260 11.60039850699194 0.78772630538909

H 1.84805709190586 14.69039987612722 -0.22389337921803

H 3.37831178434574 14.33967093289040 0.61008070774900

H 0.95910018370046 15.45982079048314 2.03785353384395

H 2.13298929890300 16.63873538034213 1.44292148977208

H 2.30854532641178 13.74793437120947 3.02601628754196

H 4.40638156568159 15.62430140280664 5.58781858531630

H 4.14259403269165 12.60199203894673 8.48189990665718

H 4.96983094716301 14.73111025202862 7.70373090349254

H 2.74473984077580 11.42160165205847 3.51263465013572

H 1.18326536713298 11.90916183477780 4.12157472009066

H 2.15959989122907 8.69900839758775 4.21132724362744

H 1.46267775447797 7.28015652753141 7.79320497465118

H -0.48122208156725 10.38666960078964 6.40167671268146

H -1.71131816914272 9.12636629669450 5.96026913928195

H -0.13929012732408 9.58764671630768 3.94555897927879

H -0.01196104815101 7.93265380003793 4.68888330820235

114

energy: -181.380445702203 gnorm: 0.161217831745 xtb: 6.4.1 (afa7bdf)

C 2.24835847173088 3.75028571310479 7.52925345419592

C 1.87447274506387 2.47648207463173 7.42721659818707

C 1.74694181649764 1.59084787460440 8.58479606679678

C 2.05143690564240 2.20826923086148 9.86501099584500

C 2.43841489685950 3.48206474160937 10.01725585877841

C 2.50453632712335 4.41733177700324 8.84145241286437

C 3.93476199206606 5.06216237061430 8.78920368058716

C 2.74962039141650 4.16250560388830 11.30884340795026

C 4.10197037231374 4.88505966968470 11.29202843093339

C 4.29523396333143 5.80528843047705 10.08371369637151

C 5.45951879631434 6.73175933451858 7.56708924060674

C 4.17178771577999 5.88235875083304 7.49675774697000

C 5.67570091724455 6.44650320745682 9.99040258285042

C 5.73100708778814 7.48966530161541 8.86465827799591

C 7.16969690699152 8.05715596515737 9.07233561060327

C 6.29462545028522 7.13092544264913 11.20642092890507

C 7.18415494176052 8.24640897706959 10.61606194409774

O 1.28380449092183 0.45512551384048 8.46230522699303

H 6.24905384566778 5.59518775914418 9.60537167709609

C 4.65662456697922 8.55066190345926 9.09798241467093

C 7.24643242303688 9.33245127411399 8.21755342096698

C 8.60184359940430 8.16557593402186 11.17259964012522

O 3.05252772045744 6.67572955897340 7.20250338943072

C 1.40028551171997 5.46631872220236 9.07674257726239

F 4.93775783984695 4.07038879530407 8.78805259711409

H 3.56480962494675 6.61443266926672 10.08919633666279

O 8.24477716802530 7.24586820503694 8.63431503868265

O 6.73080971386853 10.39583199031780 8.53852045278301

N 7.95258829705855 9.19880002343328 7.08579464467083

C 8.09588533922255 10.32585697857660 6.19214215688145

C 7.79166460871089 9.64964063582864 4.85498458595178

O 6.45807083510321 9.20240578939157 4.71560693090910

C 5.68231689623282 9.92660025721264 3.78937344542525

C 5.24320035887859 11.20053606016792 4.50263275789643

O 4.25305184180460 11.88280505649551 3.76315866033917

C 4.51749664741477 12.27179173365508 2.43430586182540

C 3.68697023404399 13.42665808703596 1.88258872994429

O 2.38816510621599 12.98716632281573 1.54915987435393

C 1.51441399862852 12.25783087350141 2.39772149440245

C 0.24508277080856 13.08109542758648 2.60782337815471

C 0.42954181011008 14.48090178451764 3.17099442040939

N 1.25150694673314 14.55177072124772 4.24935313949646

O -0.07595183759815 15.46580391947736 2.66268274077172

C 1.71951995377061 15.63141061142740 4.99949176925988

C 1.31695505086941 16.95301513822662 4.79245446248113

C 2.60131455473375 15.40329843197036 6.05184309927216

C 3.13915109223476 16.45305155877075 6.79267174670723

C 2.65117488405163 17.74192398096994 6.65651429742959

C 1.85044316479387 17.98193898366164 5.55281862538752

C 3.24153846756496 14.13533695379199 6.53411343074983

N 4.14981168053840 14.55077655310657 7.58639543165034

C 4.05577333984195 15.89384456190119 7.80014601649567

C 5.01158688059291 13.61328943914149 8.29024358526178

C 6.36149832536835 14.30442025739733 8.37579725898003

N 7.15384025113957 13.95235467586694 9.44225007530404

C 6.76471820505448 13.14508738001807 10.49206822613884

C 5.46896870315393 12.40170663609438 10.37728858543289

C 4.39986572625227 13.18495049141315 9.62699349211039

O 6.74674385567018 15.04978920097362 7.50616667203250

O 7.40414091829234 13.21532318051878 11.51959260694683

O 4.63173342813703 16.46454819516137 8.71319746663908

H 2.18576933456614 4.35282115201607 6.63462760831015

H 1.46351458206763 2.01613565726161 6.53995106165534

H 1.86105868288204 1.62721112598063 10.75711372346238

H 2.00337247632091 4.92765026854504 11.52729286507785

H 2.68761899580794 3.43466105830908 12.11769599617012

H 4.20356340819848 5.49542173618375 12.18819152002015

H 4.97651315762661 4.24333572594423 11.41762530919658

H 5.47436443193822 7.52305024475847 6.81382479737740

H 6.34532845803653 6.11111043679488 7.42922971710944

H 4.50068897619532 5.08475064570757 6.81017589743100

H 5.46517440354729 7.58933842274294 11.74368325938809

H 6.84014835784991 6.47351003313561 11.88035642152865

H 6.83433552852235 9.26885267005020 10.78394671085227

H 4.49937499845585 8.94687752909862 10.09531773468922

H 4.76578519549215 9.49645941179832 8.57730834274927

H 3.73706782421738 8.02322113950709 8.85128645492757

H 9.21968052251925 8.60255936725882 10.39049102904004

H 8.65104672651611 8.61362368256750 12.16420778877235

H 8.84549868824278 7.10833130274271 11.23965044172034

H 3.32002118409007 7.11533656004788 6.38214964554168

H 1.82172724972210 6.23187575502293 9.72440666098784

H 0.54633685382305 4.99914924420412 9.55402349305636

H 1.02468712417510 5.92113677202788 8.16819229190516

H 8.34562929372463 6.43128370357551 9.14179626250451

H 8.43006896277830 8.34494904867300 6.83872823033960

H 7.28098375218280 11.01910114109255 6.41627632977189

H 9.04029078502447 10.86445710026709 6.25009934993751

H 8.09875393865450 10.31638703009390 4.03669749060216

H 8.50231921929115 8.81710784699771 4.75186057859880

H 4.79495912222538 9.37010022897000 3.45559034272501

H 6.26219397854524 10.15001757035047 2.88196654454696

H 6.17781598211629 11.75692236223868 4.65362504160920

H 4.79399961235241 10.96798087273818 5.47962788285780

H 4.26284359681582 11.37078579759988 1.85776021987423

H 5.58620469631761 12.48775854025416 2.28364978034565

H 3.72601208187213 14.25051352735157 2.60719919656776

H 4.01834238270594 13.65606853845376 0.86077879439451

H 1.98124931794184 11.94972336607094 3.34275929608387

H 1.28478319690404 11.33650490340230 1.84399506466057

H -0.40934012313838 12.58539266142891 3.32764692539458

H -0.20328819769709 13.34380236195222 1.65004845261410

H 1.56337143414850 13.62576215042922 4.52598717187104

H 0.60251745089062 17.16210163615835 4.01398359411973

H 2.90733236866698 18.48451237087099 7.39468990000265

H 1.43083029616732 18.95004438317049 5.32942942786747

H 3.89342993780766 13.64222288955559 5.80310698352151

H 2.41919791707796 13.47103356753724 6.82528620925392

H 5.15900664770036 12.77512166800422 7.59473190699644

H 8.01979879959109 14.46950878423619 9.58495121509015

H 5.10417924396050 12.20291952537985 11.38998825220582

H 5.58560146480353 11.42593855941984 9.90239470384527

H 4.22318055778455 14.07029689422937 10.23667579963521

H 3.51814948211835 12.56722503923650 9.45070892372768

KH-99 the first, the 5001st and the last MD frames

121

energy: -191.856291593448 gnorm: 0.000594028615 xtb: 6.4.1 (afa7bdf)

C -2.68790000000000 -1.52020000000000 -0.25290000000000

C -1.52620000000000 -1.83020000000000 -0.82480000000000

C -0.58030000000000 -0.80590000000000 -1.26630000000000

C -0.96190000000000 0.56490000000000 -0.96910000000000

C -2.12200000000000 0.90190000000000 -0.38980000000000

C -3.15360000000000 -0.12200000000000 -0.00020000000000

C -4.40280000000000 0.18520000000000 -0.89980000000000

C -2.51530000000000 2.32570000000000 -0.17480000000000

C -3.78790000000000 2.62340000000000 -0.97510000000000

C -4.90610000000000 1.62120000000000 -0.67980000000000

C -6.75550000000000 -0.52160000000000 -1.67770000000000

C -5.50530000000000 -0.90250000000000 -0.86050000000000

C -6.09080000000000 1.86200000000000 -1.61050000000000

C -7.25170000000000 0.88880000000000 -1.36440000000000

C -8.28480000000000 1.45780000000000 -2.38550000000000

C -6.75260000000000 3.23760000000000 -1.58750000000000

C -8.14380000000000 2.99670000000000 -2.21520000000000

O 0.47180000000000 -1.08990000000000 -1.84270000000000

H -5.74550000000000 1.67490000000000 -2.63390000000000

C -7.83480000000000 0.94490000000000 0.04760000000000

C -9.71440000000000 0.98320000000000 -2.06650000000000

C -8.33270000000000 3.77010000000000 -3.51620000000000

O -5.81540000000000 -1.22880000000000 0.47120000000000

C -3.43840000000000 -0.02220000000000 1.51100000000000

F -3.92560000000000 0.11130000000000 -2.22340000000000

H -5.22820000000000 1.71690000000000 0.35730000000000

O -7.91950000000000 0.98640000000000 -3.67010000000000

O -10.53800000000000 1.66880000000000 -1.47550000000000

N -9.95270000000000 -0.25300000000000 -2.52460000000000

C -11.15690000000000 -1.00690000000000 -2.24940000000000

C -11.39030000000000 -1.19020000000000 -0.74820000000000

O -10.25340000000000 -1.79680000000000 -0.16160000000000

C -10.43350000000000 -2.04320000000000 1.21540000000000

C -9.10370000000000 -2.48940000000000 1.81480000000000

O -9.32130000000000 -2.84470000000000 3.16290000000000

C -8.11820000000000 -3.08260000000000 3.86190000000000

C -8.45800000000000 -3.49530000000000 5.29060000000000

O -7.25610000000000 -3.51900000000000 6.02950000000000

C -3.05630000000000 -5.96840000000000 14.38500000000000

C -2.18840000000000 -4.93140000000000 14.73180000000000

C -3.03650000000000 -7.11460000000000 15.17440000000000

C -2.17240000000000 -7.20650000000000 16.26500000000000

C -1.31350000000000 -6.17580000000000 16.60810000000000

C -1.33740000000000 -5.03670000000000 15.82100000000000

C -3.82330000000000 -8.38670000000000 15.06680000000000

N -3.32150000000000 -9.15300000000000 16.18900000000000

C -2.35330000000000 -8.52420000000000 16.89430000000000

C -3.75640000000000 -10.50770000000000 16.45530000000000

C -2.64570000000000 -11.24650000000000 17.20680000000000

N -2.89100000000000 -11.54280000000000 18.52500000000000

C -3.83700000000000 -10.97790000000000 19.35430000000000

C -4.85770000000000 -10.11460000000000 18.66920000000000

C -5.07700000000000 -10.55850000000000 17.22860000000000

O -1.65740000000000 -11.66490000000000 16.64410000000000

O -3.81250000000000 -11.19890000000000 20.54520000000000

O -1.78110000000000 -9.01090000000000 17.86480000000000

C -7.41960000000000 -4.00340000000000 7.34460000000000

C -6.08740000000000 -3.84380000000000 8.07110000000000

O -6.15230000000000 -4.54150000000000 9.29220000000000

C -4.99740000000000 -4.36090000000000 10.08700000000000

C -5.11100000000000 -5.25710000000000 11.31560000000000

C -3.99400000000000 -4.97420000000000 12.30450000000000

N -3.93600000000000 -5.90270000000000 13.30020000000000

O -3.24870000000000 -4.01840000000000 12.20930000000000

H -3.34990000000000 -2.30010000000000 0.09420000000000

H -1.22560000000000 -2.85620000000000 -0.98140000000000

H -0.24900000000000 1.32430000000000 -1.26010000000000

H -2.69860000000000 2.50890000000000 0.88470000000000

H -1.71350000000000 2.98790000000000 -0.50090000000000

H -4.13530000000000 3.62940000000000 -0.74420000000000

H -3.55040000000000 2.57770000000000 -2.03990000000000

H -7.53280000000000 -1.25910000000000 -1.46060000000000

H -6.49850000000000 -0.57960000000000 -2.73590000000000

H -5.05970000000000 -1.78230000000000 -1.35750000000000

H -6.84190000000000 3.60670000000000 -0.56650000000000

H -6.18740000000000 3.96530000000000 -2.16610000000000

H -8.93410000000000 3.30530000000000 -1.52510000000000

H -7.08540000000000 0.67700000000000 0.78490000000000

H -8.22520000000000 1.93100000000000 0.27770000000000

H -8.65620000000000 0.23510000000000 0.12400000000000

H -7.55140000000000 3.52600000000000 -4.23340000000000

H -9.30960000000000 3.56190000000000 -3.95040000000000

H -8.28220000000000 4.83620000000000 -3.31030000000000

H -6.51640000000000 -1.89250000000000 0.45860000000000

H -2.50270000000000 -0.17650000000000 2.04380000000000

H -4.14780000000000 -0.78590000000000 1.80840000000000

H -3.83940000000000 0.94680000000000 1.78320000000000

H -8.39160000000000 1.49620000000000 -4.33960000000000

H -9.18640000000000 -0.71900000000000 -2.98750000000000

H -12.02300000000000 -0.47740000000000 -2.65760000000000

H -11.05810000000000 -1.97860000000000 -2.73050000000000

H -11.56970000000000 -0.21110000000000 -0.28320000000000

H -12.27850000000000 -1.81870000000000 -0.59550000000000

H -10.77740000000000 -1.13360000000000 1.72750000000000

H -11.18690000000000 -2.82820000000000 1.37160000000000

H -8.37850000000000 -1.66620000000000 1.75190000000000

H -8.71820000000000 -3.34980000000000 1.24810000000000

H -7.53640000000000 -3.87780000000000 3.37580000000000

H -7.50140000000000 -2.17330000000000 3.89160000000000

H -8.93730000000000 -4.48380000000000 5.29350000000000

H -9.16450000000000 -2.76990000000000 5.71900000000000

H -2.18100000000000 -4.03660000000000 14.13240000000000

H -0.65560000000000 -6.27310000000000 17.45660000000000

H -0.68370000000000 -4.21120000000000 16.05420000000000

H -3.62750000000000 -8.91820000000000 14.12880000000000

H -4.90130000000000 -8.22240000000000 15.17030000000000

H -3.86010000000000 -11.01680000000000 15.48800000000000

H -2.15640000000000 -12.06810000000000 18.99190000000000

H -4.47380000000000 -9.09070000000000 18.70070000000000

H -5.78020000000000 -10.14220000000000 19.24900000000000

H -5.81150000000000 -9.91320000000000 16.74800000000000

H -5.45460000000000 -11.58150000000000 17.21200000000000

H -7.70120000000000 -5.06620000000000 7.33500000000000

H -8.20520000000000 -3.44410000000000 7.87100000000000

H -5.88080000000000 -2.77810000000000 8.24310000000000

H -5.28470000000000 -4.24710000000000 7.43610000000000

H -4.90610000000000 -3.30960000000000 10.39670000000000

H -4.09470000000000 -4.61770000000000 9.51370000000000

H -6.07110000000000 -5.06010000000000 11.79730000000000

H -5.09340000000000 -6.30120000000000 11.00300000000000

H -4.59420000000000 -6.66810000000000 13.24000000000000

121

energy: -191.766848751813 gnorm: 0.157206353487 xtb: 6.4.1 (afa7bdf)

C -4.70765283437039 -1.35682261097796 1.92680804595125

C -4.02326013389509 -1.37484808673807 0.78488062004678

C -4.49143915024545 -0.68110525876789 -0.41443955511576

C -5.73174822881795 0.07095898770018 -0.31919336721872

C -6.46860007252877 0.04350953044260 0.79941533636737

C -6.05786197268656 -0.72244859650541 2.02781280722457

C -7.18337452246451 -1.77427279931137 2.32954123670637

C -7.77154515729916 0.75706564416792 0.94531991163103

C -8.85232264342951 -0.32424511402445 1.05163654230718

C -8.54958632497098 -1.06950547316875 2.35352590635545

C -8.00356673631713 -3.51635779057424 4.03790000601140

C -6.81976082633919 -2.65934076330717 3.54797541900911

C -9.58870804619097 -2.13156342317007 2.69987863432429

C -9.34929346201029 -2.79388614932070 4.06347005050165

C -10.56197697648883 -3.77348217080919 4.00437536685460

C -11.05284879510631 -1.74539968376004 2.89469406986634

C -11.77696063193894 -2.85685311570417 3.68703924086120

O -3.85875553000512 -0.61493256129348 -1.47044144951265

H -9.57402197653015 -2.90159279507775 1.91986250840224

C -9.39417028682357 -1.82839201170053 5.24782809744532

C -10.75712237190447 -4.52034666497544 5.33661935877897

C -12.91935543316946 -3.53210976667076 2.93511214550076

O -6.41723317532481 -1.90987914362906 4.66705422680896

C -5.92321297079925 0.32913718074343 3.14621951140977

F -7.19799082260530 -2.68229889097200 1.25233512110263

H -8.53063883653626 -0.27591141834425 3.10074260466857

O -10.38885230786951 -4.80375642332258 3.04812119482949

O -11.58531029314244 -4.19470909151797 6.17668499504212

N -9.92150373314438 -5.54280851349159 5.56277465355449

C -9.81941736634595 -6.20832026107613 6.84373928477046

C -8.43143829134643 -6.83465081780524 6.99506679315368

O -7.48413423115316 -6.27706955216777 6.10269754164299

C -6.23296108863054 -5.73093574481314 6.45707248771131

C -6.22314856776525 -4.87247629261987 7.71790313598813

O -5.73434405555006 -5.62147202494822 8.80924955297295

C -4.32284421580994 -5.62391554761394 8.79166400120627

C -3.82021042350274 -6.37362026090352 10.02140833793787

O -2.46032699582024 -6.04483513871989 10.20502728191137

C -3.02477764806251 -1.63376361079714 9.56209664258959

C -3.98115199599956 -0.63317411869077 9.74391677655841

C -2.82649097881638 -2.17361605673744 8.29459486801734

C -3.53091070120892 -1.56328864775783 7.25737925052766

C -4.58530609468809 -0.68148959253486 7.42605119668707

C -4.75901292825105 -0.16176352337501 8.69780481325817

C -1.91339076986139 -3.22429259441436 7.73674420060708

N -2.21155505119515 -3.11278971808331 6.32358567606098

C -3.16249353563960 -2.21075726224313 5.98847030089779

C -1.59495165610405 -4.04402256719880 5.40278629445860

C -2.38994089854436 -4.06781732001856 4.09448275790991

N -1.79484679355418 -3.58297161218829 2.95588507004633

C -0.67751805978715 -2.77521110553683 2.92645748286113

C -0.13032333362570 -2.49990559073266 4.29804860627894

C -0.11223999408662 -3.77512095720824 5.13109532519223

O -3.49346658573192 -4.56804971399084 4.07385824967048

O -0.39507988823314 -2.27002601797731 1.86217332152554

O -3.45569028030263 -1.98949011568036 4.81747218756414

C -1.83291146593501 -6.56174780849318 11.35828989936958

C -0.48134119600130 -5.88935268054309 11.58004167011973

O -0.49032651210645 -4.48173344059056 11.60473682196461

C -1.15818439460346 -3.88103072176801 12.69617601229429

C -1.02036460446587 -2.36298137370566 12.65068692921741

C -2.20030256260772 -1.75260093924216 11.91527765197308

N -2.25765357416103 -2.14724477593648 10.61225734444632

O -3.03049747453073 -1.04158439001851 12.44755641690858

H -4.28624152949397 -1.80562700056623 2.81447553480667

H -3.04258298476297 -1.82547176251472 0.73224443758840

H -5.95086397588560 0.69446089274559 -1.17524795294933

H -7.85494612151659 1.39034843391213 1.82945710558480

H -7.90707645206551 1.37537990796246 0.05816037788007

H -9.82573109015146 0.14622915254870 0.92061158538439

H -8.74050550626270 -1.05537341202353 0.24836689453341

H -7.73683936057906 -3.73987349369095 5.07430301461930

H -8.07626222503876 -4.46135409012807 3.49855075918497

H -5.93221987629242 -3.25081887543455 3.26154720970392

H -11.15475530885904 -0.80843706416261 3.44092334223193

H -11.56899417381996 -1.56740060304604 1.95358484587763

H -12.08190188731647 -2.46798351417217 4.66266075090525

H -8.55479598381683 -2.02573156640589 5.90622762939266

H -9.39641253107222 -0.90757583511493 4.67350769582150

H -10.29244933856502 -1.84733843116902 5.86190198867683

H -13.18093878703304 -4.56695456685809 3.14738727843329

H -13.84926935837879 -2.99274641922082 3.10991993626754

H -12.80193278558802 -3.52034114635447 1.85456242690130

H -5.49080413855659 -1.64069566169670 4.63062696402338

H -6.60173817255827 0.23163710742990 3.99079994859539

H -6.18509847406976 1.28297958019429 2.70289854793179

H -4.88292702039093 0.38762636522571 3.44327001783303

H -10.38032395497197 -4.44688547965181 2.15169769041039

H -9.18754492672862 -5.88597391336525 4.96095234991656

H -9.89200927343135 -5.49185621365714 7.66747248557463

H -10.58700106652102 -6.97457187926245 6.93909788911794

H -8.00608414235836 -6.66462060411924 7.99367069127092

H -8.42787099464114 -7.92602004656492 6.86806576217719

H -5.52221758500945 -6.56838180170658 6.49466974832304

H -6.00440211400562 -5.11273087926696 5.57740292506622

H -7.26437219105426 -4.60884456396932 7.95002674101984

H -5.66134178094799 -3.93865868383634 7.56821977726443

H -3.97051200882591 -6.06983893781856 7.85137583473906

H -3.94062156808575 -4.59928331752219 8.90205177512747

H -3.92005058109221 -7.46662276291764 10.06880689841846

H -4.42417367392812 -5.97535920054932 10.84935186859225

H -3.99909109789732 -0.19182985598604 10.72620779218601

H -5.13484424690067 -0.30190101087611 6.57978154036966

H -5.54515805597480 0.54449132203820 8.91306315409390

H -1.99440074489926 -4.22389380465206 8.17822890113545

H -0.86524521673062 -2.94927495572581 7.89657841138249

H -1.58181229504098 -5.06458264431581 5.80761996680577

H -2.30684015383868 -3.83455721002410 2.11437431258402

H -0.76482591905081 -1.73671137849033 4.75813961656222

H 0.87875695221010 -2.10249655045114 4.18960928459756

H 0.50114730333918 -3.72278278316425 6.02992536997542

H 0.33244102350038 -4.62747620705617 4.61618208030106

H -1.75256343332884 -7.65738665102757 11.40361407064198

H -2.44065876748331 -6.32562703468361 12.24258114535702

H 0.06676061606321 -6.05144992681941 10.64126963359734

H 0.04934943308802 -6.51016078242321 12.31704094912769

H -0.75657667672356 -4.30257906880291 13.62917639038977

H -2.22640184543404 -4.14011204683916 12.73187990326215

H -1.03743252074786 -1.92523195807320 13.65104688603596

H -0.15820149244564 -1.93438784824531 12.13965298030045

H -1.63526765398868 -2.91114536089960 10.38470484561577

121

energy: -191.757571278689 gnorm: 0.160267022145 xtb: 6.4.1 (afa7bdf)

C -8.40501319375177 -5.33966655502526 10.88756732133974

C -9.34683551992110 -6.24258477623211 11.15294614701240

C -9.28244697709464 -7.61931647666129 10.66375955260549

C -8.19746199966413 -7.94177849511179 9.75169325781544

C -7.37136600904517 -6.99054843435201 9.29593418777651

C -7.34546403462657 -5.59758609940991 9.86456675967983

C -7.90347855008247 -4.74461160894864 8.67074642005900

C -6.38473192059937 -7.09981374845799 8.18115589834147

C -6.84058450076320 -6.27538869002076 6.97238052417284

C -6.99103212252631 -4.82470301031791 7.43590480795688

C -8.44853168132433 -2.31972321946040 7.85990843625196

C -8.18958972787819 -3.26907051153849 9.04632720332378

C -7.47383629842290 -3.92639049557169 6.30116431136493

C -7.44150991502542 -2.44988542115818 6.71860897186863

C -7.85948670943095 -1.77315932561920 5.37658101975848

C -6.71553703871033 -3.92860859470461 4.97611302016966

C -7.11112744618837 -2.60010277805249 4.29342856122960

O -10.15687090999159 -8.41277280270338 11.01818477132433

H -8.49404297244795 -4.19765107112239 6.00589814544903

C -6.10210749791361 -1.95683392978194 7.26614730157395

C -7.27464329402040 -0.34882926388180 5.37448687463698

C -8.03753683780307 -2.76386252168936 3.09285363246258

O -7.24298500731330 -2.74489152704156 9.94367579555889

C -5.91329999734271 -5.28277522459392 10.33848324783732

F -9.17465277841101 -5.22979229648929 8.30494072313341

H -6.01290843464775 -4.55279994123598 7.83317812930006

O -9.27066228459461 -1.82992053510550 5.27065237370234

O -6.17312720649190 0.03319194901919 5.00250915123179

N -8.13750411120358 0.51329497199362 5.92860415216671

C -7.90975113426892 1.92872452029847 6.12579864457152

C -7.86834442683698 2.26920836510302 7.61711756653305

O -6.67080022449202 1.73545926054621 8.15153735198606

C -5.56307967025361 2.60812551448668 8.17774332501735

C -4.40309644479613 1.63203706953016 8.34636451081637

O -3.14267055878947 2.26117594962526 8.42681521274709

C -2.42160209886439 2.16774639444175 7.21686553845801

C -2.87476942862787 3.19515563647286 6.18437406605113

O -2.12481194178637 3.09629934122654 4.99320249539102

C -2.14788993525027 -4.36309218642877 5.29433916648595

C -2.14784772475571 -5.02001896007466 6.52614134748093

C -2.90967672464013 -4.88917926397473 4.25499717793140

C -3.61837625007198 -6.08188340364509 4.39544040774002

C -3.54753784717273 -6.76684785264510 5.59692892558285

C -2.80035013133355 -6.23813288858640 6.63607148760420

C -3.23467211519318 -4.27970718355126 2.92391042396572

N -3.84041971763331 -5.40968065208431 2.24968387645710

C -4.24692403493413 -6.38102423270909 3.09909217752320

C -4.15635228338890 -5.27489697429202 0.84352399206999

C -3.86472838518362 -6.62678073606015 0.18656289493684

N -4.69597385855539 -6.90219950284739 -0.87116695360872

C -5.69453713747173 -6.14326129136572 -1.44444754182720

C -5.98623404764355 -4.84757303114395 -0.74260553323137

C -5.64881988548205 -4.94892619649588 0.73940231064559

O -2.97334588900514 -7.39371513706881 0.47926678588809

O -6.33970847216664 -6.53987629352953 -2.39003441234708

O -4.83530527418802 -7.40781611423240 2.77402946963388

C -2.58903424314070 2.35172749990589 3.88822555091987

C -2.87676204047341 0.87311855192513 4.13104141273366

O -1.88105181083515 0.10995144459271 4.76996207533924

C -0.71654176088183 -0.22589747124461 4.04247375725089

C 0.02111053411235 -1.35385459758163 4.75599961548933

C -0.64055950120857 -2.64571180417273 4.30997017416060

N -1.39309210733564 -3.18621539196109 5.30919244475151

O -0.63930709514476 -3.04775043715228 3.16260272268320

H -8.24440113134140 -4.43679394786691 11.45852376147792

H -10.12401478312001 -6.05494437826023 11.87981849375971

H -8.17718758139431 -8.95634625828888 9.37774541192269

H -5.40069260858649 -6.71865870155523 8.45699797632422

H -6.26934393778509 -8.08838092496062 7.73713579616502

H -6.22305882904648 -6.41584309011483 6.08639396506419

H -7.83836591850729 -6.63099927037475 6.70730102127645

H -8.47608273892919 -1.32460667321071 8.31181324287701

H -9.36885270101071 -2.64017716824881 7.37050192628875

H -9.12214094031992 -3.37572112493880 9.62821232161044

H -5.66950585669511 -3.87617874178754 5.27564628972108

H -6.81157653452945 -4.74070762243565 4.25844465801572

H -6.24376671703911 -2.02174334957818 3.96292621662592

H -5.82456778339067 -2.58748651373855 8.10413959699002

H -5.31305552642097 -1.91242398050196 6.52238919242155

H -6.25015385203866 -0.96998695273165 7.70038949521870

H -8.38738003365370 -1.78171034072170 2.78080047586090

H -7.44503850236046 -3.22060356193097 2.30130841238817

H -8.91001347358631 -3.32250840433089 3.42181194064694

H -6.65364851635071 -2.13157588299432 9.48696596156275

H -5.97657762359463 -4.39302240886100 10.96102215790959

H -5.18483742782942 -5.18903234342320 9.54130862758253

H -5.64614988946521 -6.10169902705087 10.99563579590105

H -9.52939122465675 -1.37644460907888 4.45921930754912

H -9.10618865334582 0.23508546277704 5.98243563261988

H -6.97123579275289 2.15775230265390 5.61212371894133

H -8.66913712475444 2.55680665366082 5.66291065389286

H -8.06349612025071 3.33055147091109 7.82330388654797

H -8.71793185220636 1.76195105820258 8.09473667362285

H -5.40201912019816 3.19783110578300 7.26440188019458

H -5.69510349007503 3.24595666875734 9.06316759036142

H -4.50904303538091 0.91597690068961 7.51958591618065

H -4.39811695051646 1.14359345139536 9.33198377632632

H -1.36380191928465 2.37353864393448 7.43095855591503

H -2.54054474877249 1.16995019009485 6.77143742908794

H -2.81860252657314 4.25349173752576 6.47357836460684

H -3.92215486544589 3.01260635970569 5.90413581335082

H -1.48509969748947 -4.80704187145087 7.34797336905550

H -3.74359660652914 -7.82589808949696 5.54975472123953

H -2.57025178358299 -6.84607860533037 7.49666015805952

H -2.33167672205385 -3.84770386356780 2.47821291639382

H -3.99286598418764 -3.55423284853470 3.23794820486838

H -3.53948756410709 -4.51465308337377 0.34641948803267

H -4.64200863352954 -7.80319403306770 -1.33900181263312

H -6.97646572812436 -4.44074625227364 -0.96771850762457

H -5.29441233325867 -4.16048815144957 -1.22967136570895

H -6.17394568438148 -5.81801989320018 1.13411451941113

H -5.95041717938251 -4.07439680333735 1.31700400117318

H -1.76815759099497 2.46265589536042 3.16518269405690

H -3.39579442320799 2.99186164150797 3.50554147442651

H -2.95949541184799 0.39329534907712 3.14569429979844

H -3.82203529811785 0.70032175453220 4.66658124751617

H -0.05096111927068 0.64308669651046 3.93593554335936

H -0.91174787246443 -0.61344581293304 3.03193564705551

H 1.08395670956632 -1.29393282569896 4.51227175481458

H -0.05302985305780 -1.34978570713627 5.84350132135899

H -1.31173763674543 -2.77415427215525 6.22912475273201

KH-102 the first, the 5001st and the last MD frames

127

energy: -189.189985703563 gnorm: 0.000676778647 xtb: 6.4.1 (afa7bdf)

C 8.73700000000000 -2.96690000000000 19.18660000000000

C 8.01090000000000 -2.97270000000000 20.30280000000000

C 7.95570000000000 -4.13620000000000 21.18710000000000

C 8.81150000000000 -5.24810000000000 20.80780000000000

C 9.55380000000000 -5.26440000000000 19.69260000000000

C 9.55620000000000 -4.12380000000000 18.71120000000000

C 8.91990000000000 -4.70840000000000 17.40030000000000

C 10.35120000000000 -6.45950000000000 19.28910000000000

C 9.80240000000000 -7.00000000000000 17.96420000000000

C 9.71500000000000 -5.91940000000000 16.88400000000000

C 8.01900000000000 -4.28170000000000 15.03290000000000

C 8.59240000000000 -3.65010000000000 16.31700000000000

C 8.99760000000000 -6.46910000000000 15.65460000000000

C 8.87000000000000 -5.44220000000000 14.52110000000000

C 8.17130000000000 -6.33910000000000 13.45390000000000

C 9.58060000000000 -7.70310000000000 14.96850000000000

C 8.96280000000000 -7.67270000000000 13.55200000000000

O 7.23910000000000 -4.16420000000000 22.18990000000000

H 7.97380000000000 -6.71300000000000 15.96100000000000

C 10.20710000000000 -4.93160000000000 13.98450000000000

C 8.25310000000000 -5.73870000000000 12.03910000000000

C 8.11470000000000 -8.90620000000000 13.25940000000000

O 9.72200000000000 -2.85210000000000 16.07610000000000

C 10.99290000000000 -3.60100000000000 18.52310000000000

F 7.65060000000000 -5.17780000000000 17.79170000000000

H 10.71400000000000 -5.57990000000000 16.60890000000000

O 6.80810000000000 -6.45650000000000 13.82330000000000

O 9.11520000000000 -6.04340000000000 11.22500000000000

N 7.27000000000000 -4.86380000000000 11.79410000000000

C 7.11400000000000 -4.15420000000000 10.54350000000000

C 7.88690000000000 -2.83230000000000 10.51600000000000

C 7.54940000000000 -2.03780000000000 9.25650000000000

C 8.48540000000000 -0.85070000000000 9.03620000000000

C 8.03420000000000 -0.00680000000000 7.84530000000000

C 9.08370000000000 1.01240000000000 7.40660000000000

C 8.56600000000000 1.88710000000000 6.26620000000000

C 9.66550000000000 2.73700000000000 5.63360000000000

C 9.10830000000000 3.65800000000000 4.54880000000000

C 11.77400000000000 9.28520000000000 4.87640000000000

C 12.82040000000000 10.18040000000000 5.09710000000000

C 11.21640000000000 8.65180000000000 5.98170000000000

C 11.71320000000000 8.89590000000000 7.26090000000000

C 12.75230000000000 9.78550000000000 7.48030000000000

C 13.29350000000000 10.42600000000000 6.37750000000000

C 10.11110000000000 7.64540000000000 6.06880000000000

N 10.06720000000000 7.35850000000000 7.48710000000000

C 10.98440000000000 8.04040000000000 8.21360000000000

C 9.19400000000000 6.33960000000000 8.03060000000000

C 9.84760000000000 5.74370000000000 9.27890000000000

N 9.25150000000000 6.03410000000000 10.48020000000000

C 8.36870000000000 7.05880000000000 10.75560000000000

C 7.81930000000000 7.77460000000000 9.55510000000000

C 7.78870000000000 6.86550000000000 8.33320000000000

O 10.77420000000000 4.96690000000000 9.20880000000000

O 8.08160000000000 7.32060000000000 11.90300000000000

O 11.13470000000000 7.92110000000000 9.42500000000000

C 11.09310000000000 8.44130000000000 1.28170000000000

C 11.90590000000000 9.09700000000000 2.40550000000000

N 11.28720000000000 8.98200000000000 3.60300000000000

O 12.97250000000000 9.64660000000000 2.19770000000000

C 10.21130000000000 4.38540000000000 3.78130000000000

C 9.63580000000000 5.44900000000000 2.84810000000000

C 10.72210000000000 6.25390000000000 2.13640000000000

O 10.15810000000000 7.50410000000000 1.75240000000000

H 8.78480000000000 -2.07910000000000 18.57290000000000

H 7.43510000000000 -2.11160000000000 20.61040000000000

H 8.80840000000000 -6.09430000000000 21.48140000000000

H 11.40100000000000 -6.19000000000000 19.16830000000000

H 10.28300000000000 -7.23110000000000 20.05580000000000

H 10.43750000000000 -7.81120000000000 17.61110000000000

H 8.80070000000000 -7.39860000000000 18.13880000000000

H 7.93930000000000 -3.49760000000000 14.27620000000000

H 7.01780000000000 -4.65030000000000 15.25860000000000

H 7.78720000000000 -3.02910000000000 16.74810000000000

H 10.66750000000000 -7.64670000000000 14.92410000000000

H 9.30730000000000 -8.61800000000000 15.49030000000000

H 9.74720000000000 -7.61970000000000 12.79190000000000

H 10.74440000000000 -4.38320000000000 14.75070000000000

H 10.82990000000000 -5.74530000000000 13.62850000000000

H 10.03350000000000 -4.26280000000000 13.14360000000000

H 7.34320000000000 -9.04220000000000 14.01500000000000

H 7.65730000000000 -8.83330000000000 12.27340000000000

H 8.75050000000000 -9.78780000000000 13.26580000000000

H 9.51830000000000 -2.22730000000000 15.36830000000000

H 11.37570000000000 -3.30150000000000 19.49620000000000

H 10.99500000000000 -2.74350000000000 17.86000000000000

H 11.64670000000000 -4.35810000000000 18.10690000000000

H 6.42200000000000 -7.21230000000000 13.36420000000000

H 6.61100000000000 -4.69210000000000 12.54000000000000

H 7.48860000000000 -4.80450000000000 9.74840000000000

H 6.04990000000000 -3.96980000000000 10.38540000000000

H 7.63840000000000 -2.24350000000000 11.40130000000000

H 8.95620000000000 -3.05190000000000 10.54360000000000

H 6.52000000000000 -1.67950000000000 9.32360000000000

H 7.61370000000000 -2.70030000000000 8.39060000000000

H 9.49720000000000 -1.22130000000000 8.85730000000000

H 8.51590000000000 -0.22960000000000 9.93380000000000

H 7.81750000000000 -0.66910000000000 7.00360000000000

H 7.10820000000000 0.51250000000000 8.10220000000000

H 9.37000000000000 1.64240000000000 8.25230000000000

H 9.97990000000000 0.48150000000000 7.07680000000000

H 7.77200000000000 2.53580000000000 6.64320000000000

H 8.12920000000000 1.24630000000000 5.49660000000000

H 10.16320000000000 3.32680000000000 6.40860000000000

H 10.41790000000000 2.07730000000000 5.19580000000000

H 8.43780000000000 4.38880000000000 5.00690000000000

H 8.51580000000000 3.06730000000000 3.84790000000000

H 13.27120000000000 10.67250000000000 4.25110000000000

H 13.12100000000000 9.95810000000000 8.47870000000000

H 14.10310000000000 11.12590000000000 6.50970000000000

H 10.33230000000000 6.73260000000000 5.50530000000000

H 9.15150000000000 8.05130000000000 5.73000000000000

H 9.14610000000000 5.52630000000000 7.29410000000000

H 9.68220000000000 5.60030000000000 11.29270000000000

H 8.47360000000000 8.63340000000000 9.37860000000000

H 6.82920000000000 8.15760000000000 9.80260000000000

H 7.40660000000000 7.41270000000000 7.47180000000000

H 7.12980000000000 6.01730000000000 8.52280000000000

H 11.79150000000000 8.00210000000000 0.55890000000000

H 10.52020000000000 9.22350000000000 0.77460000000000

H 10.40510000000000 8.47760000000000 3.56340000000000

H 10.78840000000000 3.66310000000000 3.20150000000000

H 10.90130000000000 4.84840000000000 4.49240000000000

H 8.98610000000000 4.98180000000000 2.10860000000000

H 9.02320000000000 6.14860000000000 3.41980000000000

H 11.09270000000000 5.72680000000000 1.24780000000000

H 11.57660000000000 6.41870000000000 2.80830000000000

127

energy: -189.075312154626 gnorm: 0.148220962548 xtb: 6.4.1 (afa7bdf)

C 12.92148110154058 -0.07543121077079 16.23383975724936

C 12.46473859447347 -0.04861349395273 17.48436944634380

C 12.46698762655309 -1.26866962881162 18.29073405649916

C 13.06607308004552 -2.38086695801294 17.57191724986631

C 13.62274034259775 -2.42459021264336 16.35406832262729

C 13.28304701014551 -1.28475141736234 15.43238712788778

C 12.02993918423397 -1.85772307127909 14.67971314609231

C 14.13519307536743 -3.68619860522401 15.74360979305429

C 13.06864213827270 -4.19458216270895 14.76754779313924

C 12.43796329401320 -3.11196102697953 13.88871736257782

C 10.04998931230813 -1.36839930504025 13.15400190636400

C 11.25729954101917 -0.76495097432147 13.89889863460450

C 11.18200881492935 -3.65196644003057 13.21105239547905

C 10.47666918447367 -2.59507030662170 12.35018407458220

C 9.33422944340532 -3.43332000208245 11.69926464284200

C 11.38611253384833 -4.79260834269309 12.21573537031044

C 10.05754249608906 -4.78116030994978 11.42589749076800

O 12.10160709507719 -1.48926426480727 19.44734226804379

H 10.42212691580402 -4.04633129188483 13.89558910510237

C 11.33858941964259 -2.11881036516755 11.18109966374291

C 8.83130823329458 -2.83050936084866 10.37543682538918

C 9.09244402438518 -5.93411296310327 11.68220933518926

O 12.11425674848185 -0.00331078608007 13.08873717328957

C 14.47234612820131 -0.85148257898538 14.55451799622974

F 11.07724775112395 -2.21865682286475 15.65272843384590

H 13.18431041396602 -2.82778010393849 13.14632360710695

O 8.11969075878495 -3.67614701143654 12.38809280035198

O 9.20263423340669 -3.31914372030791 9.31611118854920

N 8.10664499230907 -1.71412287629192 10.51863271164467

C 7.40132739169978 -0.88918402955481 9.56266643447172

C 5.88376316215327 -0.83357057945009 9.76130236611824

C 5.25408692088910 -0.06403736428496 8.60250850369943

C 5.03900877942967 -0.85230381654026 7.31169019937510

C 4.16372696749622 -0.08361132891836 6.32326464845858

C 4.79645449592359 1.07781757324873 5.55944833531861

C 3.98059841266989 1.44384473195377 4.32088928924587

C 4.41750400022557 2.80021419388711 3.77244748707934

C 3.90809056999217 4.02053963187910 4.53848120864611

C 9.12592011680943 3.78114321643725 7.56083315313789

C 9.38136345293363 2.72660931301385 6.68461885832677

C 9.35660651768720 3.55630440230857 8.91362018987546

C 9.80140533239998 2.28695869581522 9.27922000326506

C 10.07806266739058 1.24795357227053 8.40561687066277

C 9.73863955972304 1.44522498770967 7.07701553999484

C 9.29789466941244 4.42747812697956 10.13005956899653

N 9.78132559296348 3.55082142537961 11.17580393816118

C 9.96625094204945 2.28087390527100 10.74327461405828

C 10.18375218501425 4.00702678465413 12.48956084177975

C 9.75542924859408 2.99861448517238 13.55735611020481

N 10.65917471446989 2.69660577412686 14.54468782284787

C 11.82650173921429 3.37474013269666 14.83221604733666

C 12.23621854536867 4.49905492320851 13.92476983687232

C 11.69740731334336 4.23193326396881 12.52521017582583

O 8.63432399871225 2.54781145086743 13.63993601106351

O 12.52908512477722 3.03279997055449 15.75792977789263

O 10.24212323451636 1.29881561187802 11.42428014564270

C 8.13691638925633 7.28288612818079 6.62355942391462

C 8.19703025771047 6.12294388812250 7.62577353262530

N 8.81380731391873 5.05256727441346 7.07450141414555

O 7.74736134075528 6.23312700155263 8.75206573673089

C 4.60020918886646 5.35116773309074 4.24663076092371

C 5.81407361589415 5.51600903237222 5.15913717145517

C 6.33907368231651 6.95084212714267 5.16672080601465

O 7.75621372471668 6.99482090630681 5.30211655759681

H 12.94235159809763 0.86598329184800 15.70430733309166

H 11.94059163536026 0.85048793815435 17.77513267168804

H 12.96728335681285 -3.29184933863954 18.14650646402182

H 15.05241074898906 -3.37662030004316 15.24143853333749

H 14.25991445612425 -4.46289424443414 16.49795400335102

H 13.44914414433921 -5.02768831743027 14.17828601114535

H 12.29265847452954 -4.69537389427374 15.35049464338670

H 9.64494388096021 -0.63828804776020 12.44927815420437

H 9.27101471955019 -1.70152906238195 13.84061028524434

H 10.80972935139208 -0.15037617109779 14.70003159563075

H 12.33393485079209 -4.56984399222555 11.72735760281901

H 11.47723777710828 -5.75860512908686 12.70823567727994

H 10.33419567565919 -4.77939718249635 10.36792570832835

H 10.75291140519085 -1.30381519170765 10.76968694101315

H 12.26764564860117 -1.71233649563576 11.56627112794257

H 11.62071132831431 -2.88741793124966 10.46402724015086

H 8.90748244770612 -5.98251170693927 12.75369726827718

H 8.18777673334703 -5.91219026305335 11.07572630785883

H 9.64204956777354 -6.81223091073544 11.35305317082201

H 11.44882855999086 0.41749350824756 12.52929222970485

H 15.40846430935941 -1.11823766926385 15.03999117136589

H 14.39549132487296 0.20375712793905 14.31877491986905

H 14.55045094829759 -1.36234542582712 13.60225956870821

H 8.31960114689197 -4.33019760088162 13.06877872477958

H 7.96220701592561 -1.46316010297890 11.48625043960751

H 7.80329801079006 0.12452203111896 9.64152649613370

H 7.61375047623021 -1.32272534021347 8.58377616988099

H 5.44245896215345 -1.83031223759479 9.82415499039168

H 5.60594231508218 -0.42205091625946 10.73385342198752

H 5.81178070678537 0.76557496469417 8.16288743304061

H 4.29701848916238 0.30203437427845 8.98044374946089

H 4.47407494877717 -1.75152334735459 7.56731260195781

H 5.98652341664563 -1.07038145670659 6.81465964148555

H 4.00528959308512 -0.83328240090558 5.54419472071856

H 3.28419893707576 0.25143390248494 6.87763026494174

H 5.13290423507723 1.94666707840509 6.13039342067085

H 5.75931397798895 0.64812381337732 5.27298336264784

H 2.92631817659953 1.48686111555398 4.60376640850797

H 4.08910849487889 0.67948892013110 3.54778593585591

H 5.51102456542724 2.78003739289574 3.77246194220620

H 4.01204904948675 2.94599339154520 2.76878748564649

H 3.98790676508301 3.98785029517747 5.62754286085118

H 2.83127761490213 4.09216784087745 4.37542658752519

H 9.14982777417295 2.85108034846593 5.63963213106238

H 10.44207598883495 0.31716998526726 8.81017742231505

H 9.82916191934047 0.67138121090831 6.33152205631103

H 8.29034743752956 4.68724082656430 10.47216694300538

H 9.91473630261476 5.32859767677621 10.04138953058673

H 9.50520257392229 4.83125473221170 12.74724720060490

H 10.33838171408540 2.05606875307777 15.26624540712355

H 13.32711976002107 4.48182616538847 14.00499380656538

H 11.80007807419161 5.37441463914694 14.40619492230274

H 12.07452020610439 3.25959779027864 12.20931810703385

H 11.91970491646178 5.04297524719625 11.83070655269764

H 9.06683144252245 7.85431967111842 6.73228267837060

H 7.35118542348164 7.94903122106012 6.99244860802305

H 8.91748949834001 5.16480474586113 6.06914540666932

H 3.81637165259319 6.10306239435784 4.35237987534382

H 4.98577341043901 5.45537052906984 3.22848822746106

H 5.65205011715032 5.13611326870139 6.16743959378799

H 6.62224334779872 4.95185249175459 4.68993690256635

H 5.75660629167707 7.65583722448725 5.77376477677030

H 6.19529853291763 7.29243394011911 4.13162533207047

127

energy: -189.072087200835 gnorm: 0.163548739934 xtb: 6.4.1 (afa7bdf)

C 10.26324213107500 4.22876173957593 13.21695662962614

C 10.64142720059924 4.62760567640653 14.42982796486678

C 10.79681872618375 6.04842208467061 14.73950522944502

C 10.50427740285833 7.01934295902872 13.69820028199923

C 10.11368754160281 6.64177923838239 12.47352430694180

C 9.94131438560693 5.18991891696276 12.11784296643753

C 10.77910580940417 4.94138915874086 10.81345338379868

C 9.82183462173791 7.66149427049864 11.42379759515909

C 10.54762323871807 7.34930822532738 10.11061349973589

C 10.33191479780938 5.89333311513288 9.69135996971409

C 11.52937366492492 3.13883241469871 9.12023150406062

C 10.86714074688414 3.43048218493741 10.48148347157084

C 11.12027697519534 5.52581629240291 8.43769586434068

C 10.92925195896207 4.05226373436721 8.05327430436722

C 11.57230989543981 3.89424193960945 6.64152618279003

C 10.74423450093862 6.19062620761837 7.11482094444030

C 11.19185724349797 5.24599271363501 5.97623642880212

O 11.14971321891426 6.34986467795268 15.88165462957798

H 12.19009548226565 5.61417097285016 8.65954362250570

C 9.46044470853349 3.65710171192092 7.90181283361125

C 11.18986042589694 2.71566533286158 5.72855299579002

C 12.18320233220120 5.96957466448405 5.07065666866692

O 9.68470571633685 2.72489153625981 10.75501996934882

C 8.42284743905034 4.95058924232376 12.01890965277443

F 12.07546536357384 5.18782159344649 11.30678469627851

H 9.26093497956176 5.75820428417305 9.53746488642310

O 12.95374715524003 3.68875182158962 6.88227143531195

O 10.52794254900984 2.80892230045645 4.70288970818733

N 11.73976652557960 1.60298043156530 6.23004698363575

C 11.60137413239286 0.25220357786786 5.73193257780255

C 10.88113279571429 -0.76301070395911 6.62420083958861

C 9.41432841194568 -0.34631977377708 6.70376414230990

C 8.71367053818173 -1.14544445696498 7.80117599977728

C 8.28674644269464 -2.60163159709753 7.62440979904699

C 7.84419346444679 -3.02734241466342 9.02283524979397

C 7.11249400542633 -4.36793560903851 8.99041104741644

C 6.98063805073851 -5.03593611308942 10.35708670530316

C 5.76260215247160 -4.40203285895330 11.02795114397107

C 6.27579059001666 -1.40718331508928 13.32239410164932

C 5.55223381351585 -0.39358944802434 12.69459014594325

C 7.61318399795566 -1.50764899866407 12.95485189469794

C 8.11700364542177 -0.58201967613850 12.04264353569537

C 7.42579198912426 0.49927960410215 11.52084674783496

C 6.09672505878679 0.60263202595868 11.89794759850328

C 8.69835228988604 -2.51046793952375 13.19750322874721

N 9.85999893501439 -1.96774946704110 12.52530714093900

C 9.54863119322468 -0.85224323620070 11.82335140825372

C 11.24004551757812 -2.31065471795209 12.79718074849009

C 11.74603348397976 -1.56279322994572 14.03217958365128

N 12.97180857796284 -1.95144890330173 14.51093975169600

C 13.67390899965380 -3.06957852636338 14.10840743248968

C 12.94317678472571 -4.05042636372973 13.23698432718660

C 11.44828989421419 -3.79927531717038 13.08640163844031

O 11.11036303490497 -0.68947006052734 14.58003866374944

O 14.76109946380118 -3.32277783399950 14.57893844407901

O 10.30370596117772 -0.26408828717871 11.05637778781417

C 5.08065081524712 -4.05010148076938 15.77106440135355

C 6.05457240797981 -3.07462989145253 15.09768132380939

N 5.58066073176702 -2.38854087519234 14.03245333382946

O 7.19542731941569 -2.95845029654138 15.50735070792737

C 5.54040176004890 -5.05308790295645 12.39233024291856

C 4.04022827620861 -5.23039703236771 12.61893218893424

C 3.86635091746525 -5.40755229602852 14.12651677014449

O 4.00945250705499 -4.20430398288091 14.87502694304558

H 10.01669596420519 3.20819753372565 12.96246680781237

H 10.83814010238997 3.89368340766307 15.19815000472271

H 10.72524063196764 8.05982089248695 13.89411338411944

H 8.76467543432408 7.90020067158066 11.30240301905340

H 10.28146157081613 8.53522004982561 11.88552589699211

H 10.36524714416091 8.09769188386254 9.34071875038158

H 11.56784962138366 7.38069205412810 10.49910184818707

H 11.46762287094783 2.10320725624857 8.77755502996766

H 12.59181440874543 3.38357736270757 9.14319823789405

H 11.60239567474269 2.93069076181403 11.13680322634676

H 9.67443205479796 6.31243367014490 7.27971363966470

H 11.23983460271768 7.15927130084680 7.12565446613585

H 10.34476567709386 4.99380163660197 5.33227997692017

H 8.84546934630135 3.75106469317283 8.79033551436272

H 8.95701021914199 4.22693695747730 7.12814298162139

H 9.47810467122893 2.59069094544435 7.68501934994264

H 12.83958687105450 6.68254038327794 5.56611487313842

H 12.94543178757894 5.29878024151239 4.67598430992090

H 11.66057721212267 6.51423041460488 4.28853502906090

H 9.89182987539885 1.81532110534524 10.50475853604036

H 7.85149438369741 5.69043179091577 11.46271868371160

H 7.90738267168534 4.94645016196376 12.97247727808007

H 8.32949642930570 3.92571701374854 11.68008846431142

H 13.47152022038784 3.84514990830225 6.08319049285358

H 12.29423757000448 1.83844430711903 7.04075490046544

H 11.04553407877289 0.35060775160217 4.79557916460862

H 12.58852873735229 -0.15406948605121 5.50438969265961

H 10.99846245661608 -1.80279549882805 6.31231125730904

H 11.34159956827259 -0.79738315574050 7.61373471795917

H 9.01890128384675 -0.56574935085665 5.70976529562428

H 9.34541164169373 0.71488256327995 6.95260872985451

H 7.79328717387322 -0.65012568776170 8.11841816223275

H 9.37266049399175 -1.16909402052772 8.67155187050240

H 7.47957187202097 -2.63713474991725 6.88870074743501

H 9.07863009572871 -3.20281746238663 7.17203613108738

H 8.71499676327593 -3.17819218053618 9.66550323786958

H 7.21324928310693 -2.26992321039663 9.49402828778919

H 7.67447098547844 -5.05603281920604 8.35473755161391

H 6.10201578310045 -4.27577209467337 8.58526577860706

H 6.83533007941440 -6.08517404710558 10.08472723453414

H 7.78685413580596 -4.90783021517599 11.08274722726003

H 4.84972068571376 -4.50644944903695 10.43698659050892

H 5.81758349320259 -3.31547761041220 11.11523398372815

H 4.52595153273534 -0.16238070944831 12.92779893945966

H 7.90534882139773 1.17767956821446 10.83358272258092

H 5.41694100714782 1.37183610853835 11.56778429501784

H 8.49995998147560 -3.49485531896568 12.76013886403799

H 8.90242721949658 -2.66893206151464 14.26221681664798

H 11.61045756528517 -1.94225621136920 11.83112637945290

H 13.27194175111123 -1.48021645645632 15.36046977131106

H 13.10321520924533 -5.08038836958729 13.56919534264475

H 13.32858481981341 -3.94825605559289 12.22245727313672

H 10.90524249533312 -4.02692928087378 14.00330480789026

H 11.00150632310330 -4.43479646016572 12.32087048844970

H 4.74870223578023 -3.58730811859759 16.70847119908086

H 5.60153600504992 -4.99614178379878 15.94681434247852

H 4.62334735919962 -2.57467028953359 13.74436489290577

H 5.97559559918548 -4.39193396093181 13.14360351378561

H 6.05410909917257 -6.01778942865229 12.43217928628581

H 3.48712392093036 -4.34166478370231 12.31648096485838

H 3.65582854566778 -6.09680573377262 12.07751495621659

H 4.51418298581605 -6.18351467104631 14.55428392129916

H 2.83295366795896 -5.70933481697937 14.34966769755170

KH-103 the first, the 5001st and the last MD frames

121

energy: -182.860963905650 gnorm: 0.000512881329 xtb: 6.4.1 (afa7bdf)

C -2.59050000000000 -0.79320000000000 0.47900000000000

C -1.28040000000000 -0.87260000000000 0.25480000000000

C -0.46080000000000 0.31410000000000 0.01330000000000

C -1.15980000000000 1.58480000000000 0.11080000000000

C -2.47580000000000 1.69070000000000 0.33920000000000

C -3.36260000000000 0.48680000000000 0.50780000000000

C -4.35410000000000 0.52680000000000 -0.70850000000000

C -3.17370000000000 3.00980000000000 0.35060000000000

C -4.20910000000000 3.03990000000000 -0.77870000000000

C -5.15630000000000 1.83890000000000 -0.73190000000000

C -6.21180000000000 -0.63800000000000 -2.04830000000000

C -5.21150000000000 -0.75250000000000 -0.88120000000000

C -6.05130000000000 1.82960000000000 -1.96780000000000

C -7.03190000000000 0.64920000000000 -1.99030000000000

C -7.80230000000000 0.98850000000000 -3.30340000000000

C -6.93630000000000 3.04710000000000 -2.22700000000000

C -8.02740000000000 2.52190000000000 -3.18760000000000

O 0.74210000000000 0.23850000000000 -0.24660000000000

H -5.39880000000000 1.70190000000000 -2.83920000000000

C -7.99960000000000 0.61740000000000 -0.80740000000000

C -9.13810000000000 0.23050000000000 -3.40060000000000

C -8.01430000000000 3.24200000000000 -4.53200000000000

O -5.82880000000000 -1.07930000000000 0.33650000000000

C -4.06130000000000 0.54700000000000 1.87990000000000

F -3.52850000000000 0.52220000000000 -1.85020000000000

H -5.77020000000000 1.88080000000000 0.16820000000000

O -6.97730000000000 0.61130000000000 -4.39180000000000

O -10.20900000000000 0.70390000000000 -3.04240000000000

N -9.00170000000000 -0.99260000000000 -3.92750000000000

C -10.09220000000000 -1.93050000000000 -4.07650000000000

C -10.20110000000000 -2.88310000000000 -2.88310000000000

C -11.29900000000000 -3.92020000000000 -3.10630000000000

C -11.56620000000000 -4.75840000000000 -1.85790000000000

C -12.60020000000000 -5.85100000000000 -2.12290000000000

C -13.02480000000000 -6.56900000000000 -0.84370000000000

C -13.98210000000000 -7.72380000000000 -1.13130000000000

C -14.56430000000000 -8.32000000000000 0.14870000000000

C -15.43610000000000 -9.53850000000000 -0.14460000000000

C -18.12310000000000 -14.74240000000000 -0.32430000000000

C -19.16310000000000 -15.58500000000000 0.07090000000000

C -17.32610000000000 -15.15410000000000 -1.38790000000000

C -17.56610000000000 -16.37360000000000 -2.01930000000000

C -18.59830000000000 -17.20940000000000 -1.62610000000000

C -19.39320000000000 -16.79220000000000 -0.57130000000000

C -16.14800000000000 -14.49080000000000 -2.03390000000000

N -15.78600000000000 -15.44500000000000 -3.06190000000000

C -16.55780000000000 -16.55640000000000 -3.07630000000000

C -14.63080000000000 -15.26000000000000 -3.91460000000000

C -14.10140000000000 -16.63420000000000 -4.33200000000000

N -14.24810000000000 -16.97200000000000 -5.65430000000000

C -15.11040000000000 -16.41390000000000 -6.57540000000000

C -15.78750000000000 -15.14800000000000 -6.13490000000000

C -14.93170000000000 -14.38310000000000 -5.13330000000000

O -13.47580000000000 -17.33970000000000 -3.57080000000000

O -15.27210000000000 -16.94130000000000 -7.65400000000000

O -16.39860000000000 -17.49670000000000 -3.84840000000000

C -16.16790000000000 -10.02440000000000 1.10060000000000

O -16.89180000000000 -11.19820000000000 0.76350000000000

C -17.73660000000000 -11.65460000000000 1.78770000000000

C -18.37630000000000 -12.99020000000000 1.41690000000000

N -17.84430000000000 -13.52490000000000 0.29720000000000

O -19.25270000000000 -13.48120000000000 2.10630000000000

H -3.16180000000000 -1.68590000000000 0.68890000000000

H -0.76110000000000 -1.82020000000000 0.24930000000000

H -0.55090000000000 2.46760000000000 -0.02920000000000

H -3.67240000000000 3.16340000000000 1.30840000000000

H -2.45310000000000 3.81510000000000 0.20890000000000

H -4.78970000000000 3.95910000000000 -0.71530000000000

H -3.68270000000000 3.03140000000000 -1.73550000000000

H -6.86420000000000 -1.51410000000000 -2.02290000000000

H -5.64530000000000 -0.65440000000000 -2.97990000000000

H -4.49670000000000 -1.54810000000000 -1.15720000000000

H -7.37410000000000 3.41390000000000 -1.29940000000000

H -6.37620000000000 3.85990000000000 -2.68490000000000

H -9.02040000000000 2.65340000000000 -2.74850000000000

H -8.69070000000000 -0.21550000000000 -0.92130000000000

H -8.59160000000000 1.52480000000000 -0.75420000000000

H -7.46260000000000 0.48840000000000 0.12600000000000

H -8.25650000000000 4.29060000000000 -4.37880000000000

H -8.76450000000000 2.82250000000000 -5.20130000000000

H -7.03220000000000 3.18790000000000 -4.99830000000000

H -6.40360000000000 -1.84350000000000 0.19950000000000

H -4.70560000000000 1.41370000000000 1.96650000000000

H -4.65970000000000 -0.34350000000000 2.03370000000000

H -3.29480000000000 0.60040000000000 2.64980000000000

H -7.28750000000000 1.05510000000000 -5.19050000000000

H -8.06940000000000 -1.28210000000000 -4.18750000000000

H -11.01180000000000 -1.34570000000000 -4.15970000000000

H -9.94110000000000 -2.49570000000000 -4.99830000000000

H -9.24460000000000 -3.38740000000000 -2.73000000000000

H -10.42410000000000 -2.29700000000000 -1.98890000000000

H -11.01250000000000 -4.57660000000000 -3.93060000000000

H -12.21970000000000 -3.40860000000000 -3.39470000000000

H -11.92640000000000 -4.10540000000000 -1.05970000000000

H -10.63510000000000 -5.21580000000000 -1.51660000000000

H -13.48130000000000 -5.40450000000000 -2.58960000000000

H -12.18620000000000 -6.57600000000000 -2.82700000000000

H -12.14050000000000 -6.95020000000000 -0.32830000000000

H -13.51130000000000 -5.85290000000000 -0.17730000000000

H -13.45260000000000 -8.50010000000000 -1.68700000000000

H -14.79880000000000 -7.36430000000000 -1.76110000000000

H -13.75250000000000 -8.60670000000000 0.82040000000000

H -15.16110000000000 -7.56020000000000 0.65830000000000

H -14.81800000000000 -10.35130000000000 -0.52750000000000

H -16.17710000000000 -9.29070000000000 -0.90610000000000

H -19.78890000000000 -15.28600000000000 0.89540000000000

H -18.76240000000000 -18.14830000000000 -2.13000000000000

H -20.20700000000000 -17.41530000000000 -0.23590000000000

H -15.31570000000000 -14.35110000000000 -1.33530000000000

H -16.40700000000000 -13.52450000000000 -2.47970000000000

H -13.84230000000000 -14.80320000000000 -3.30240000000000

H -13.84930000000000 -17.86970000000000 -5.91680000000000

H -16.73730000000000 -15.44420000000000 -5.68010000000000

H -16.01330000000000 -14.54990000000000 -7.01760000000000

H -15.44950000000000 -13.47830000000000 -4.81650000000000

H -13.98900000000000 -14.09550000000000 -5.60020000000000

H -15.45620000000000 -10.24960000000000 1.90840000000000

H -16.86080000000000 -9.25170000000000 1.46130000000000

H -18.54040000000001 -10.93380000000000 1.99700000000000

H -17.17880000000000 -11.80620000000000 2.72520000000000

H -17.12390000000000 -12.95130000000000 -0.13040000000000

121

energy: -182.754096015048 gnorm: 0.166269079107 xtb: 6.4.1 (afa7bdf)

C -13.22039016126526 -0.51573436230757 -2.89767867355543

C -13.32303686910808 0.78904114728566 -2.65281842715147

C -12.30346900634924 1.74155243670547 -3.09045631953312

C -11.10271324236156 1.11117978913144 -3.61352578904671

C -10.87169007082390 -0.20430715478608 -3.72010803594275

C -11.98605563134451 -1.17221490875096 -3.42760265046619

C -11.41291216162100 -2.06100524984356 -2.26759564760766

C -9.62940867189172 -0.83986869879217 -4.24927496504850

C -9.06564801012943 -1.62696326237732 -3.06144765077043

C -10.07569521251805 -2.70705884169080 -2.66777611854710

C -11.72172717505848 -3.87192992675052 -0.56573972142214

C -12.45854762047879 -2.97475736737653 -1.57964789746443

C -9.54046453720407 -3.45589776250706 -1.45067764070002

C -10.45586574266498 -4.60459526554885 -1.00574903805103

C -9.64139124362109 -5.42096615787112 0.04450908986639

C -8.19620297420038 -4.15727625851426 -1.63437563395417

C -8.28643800573519 -5.39752731204542 -0.71637695161302

O -12.36647632455768 2.93289048831591 -2.77904740886292

H -9.43358302384243 -2.70411157254700 -0.66023765515753

C -10.90738600233849 -5.43721365411159 -2.20557528575504

C -10.23573808425485 -6.83346952777798 0.18578740167412

C -7.07168583933833 -5.57918161424101 0.18778336352555

O -13.08338666979937 -3.72848093782108 -2.58569350617608

C -12.40280834011674 -1.96214170822415 -4.68330842050448

F -11.03977462325069 -1.11287008338261 -1.29449570469452

H -10.14768124305387 -3.41622817715812 -3.49282502584051

O -9.61184262251178 -4.85609013771169 1.34357081758777

O -9.93786811521652 -7.78252414006820 -0.52822271253320

N -11.14285603963394 -6.94896040068084 1.16354047047575

C -11.75050840257418 -8.17888190851406 1.62081430054738

C -11.80264896453638 -8.29139865834194 3.14663567775930

C -10.52496253210786 -7.90404117537771 3.88707515660872

C -9.30276716076143 -8.79976037117439 3.69627883322679

C -9.58984146804726 -10.29054208564940 3.86454642394404

C -8.44887609128125 -11.22831368630978 3.47603100067821

C -8.46123764322600 -11.63460643176740 2.00379681852198

C -8.04749854518523 -10.47380880350963 1.10147752627551

C -7.79502197701770 -10.99284035444136 -0.31190996912478

C -9.20530818566553 -7.70617771150151 -4.32167058834310

C -8.84128785932525 -6.58886582940386 -5.07457249070759

C -10.56166343618143 -7.89873433802450 -4.07841477736049

C -11.47793056751819 -7.11083252462150 -4.77347708987831

C -11.11544279775606 -6.11150943169190 -5.66148655680400

C -9.76074417554430 -5.87731110655301 -5.83021044913572

C -11.36211271184509 -8.88668140804982 -3.28567007063810

N -12.75362119627699 -8.52534277609687 -3.46305168470196

C -12.82844914843475 -7.47826837388553 -4.31694196996242

C -13.77663082427896 -8.90131159339776 -2.51020954427629

C -14.64626690234610 -7.67468348972103 -2.22380180951314

N -15.98404201215787 -7.75669338810441 -2.52000980654821

C -16.59824930106685 -8.62573942427976 -3.39805907635194

C -15.62353936456820 -9.55335037550308 -4.06475686869294

C -14.51791294110228 -10.07527486208282 -3.15599217646753

O -14.15338996974162 -6.75103641649615 -1.61349083836594

O -17.76144469530404 -8.52083333161904 -3.72000131561539

O -13.83745790061938 -6.83939986294349 -4.59871416439910

C -7.01496439803313 -9.97836696957366 -1.13925936109228

O -6.78754005077893 -10.58345077661846 -2.40323159875805

C -6.19227954032861 -9.82172297721713 -3.42127351373033

C -7.03752797081048 -8.84088056623680 -4.23000053257005

N -8.24077715108699 -8.51068427406097 -3.71442506163793

O -6.63958228145839 -8.39510793201476 -5.29177091265253

H -14.05291531000093 -1.18623639716028 -2.74056759167811

H -14.27934744858304 1.13634777426643 -2.28881748488433

H -10.34753440818640 1.82176623949972 -3.92088043669089

H -9.89453766396982 -1.42829309258661 -5.12855810640856

H -8.91835110295915 -0.08129653062066 -4.57610327130832

H -8.16183811334293 -2.13402377710434 -3.39620407964179

H -8.87216238922805 -0.91181279253937 -2.25910198216943

H -12.41564952133697 -4.66734286512694 -0.28358343840471

H -11.58903293108424 -3.15469810464477 0.24484064149634

H -13.14164219614110 -2.34559225112895 -0.98162828134092

H -8.07057029351706 -4.45339224553865 -2.67513717935804

H -7.39216444352307 -3.52698878848373 -1.25982522491572

H -8.21492101708881 -6.24100725565204 -1.40889191110048

H -9.99473781590239 -5.88776074579932 -2.59079656989801

H -11.47616922757205 -4.86357862672508 -2.92952107126733

H -11.57260394563466 -6.26332023804175 -1.97912758241489

H -7.12046303850798 -6.55937208245755 0.65526648844971

H -6.89845236711367 -4.87456721408746 1.00034197247799

H -6.20836516560501 -5.52400102959394 -0.47290977897100

H -13.70646577124972 -4.38184027008300 -2.24209534405513

H -12.02325028451904 -2.95678976324290 -4.88431058869444

H -13.47847092685109 -2.09412549987807 -4.66726067957105

H -12.14673719098789 -1.41164408708524 -5.58580482360511

H -9.72985400844202 -3.89841401911973 1.33774532280661

H -11.21872892936540 -6.11800281332432 1.73305459283695

H -12.73420211112187 -8.18429524772598 1.14447951823626

H -11.17602531425888 -8.88062627512056 1.01293148594882

H -12.60175324503347 -7.62008098306607 3.46821813724018

H -12.09157089162501 -9.32547546614566 3.34674846125493

H -10.76973139244852 -7.76643523484648 4.94233176540459

H -10.31483095028834 -6.87866239908246 3.57556160366657

H -8.68373318774716 -8.42093236880719 4.51271166264892

H -8.74708179803240 -8.55854171181086 2.78761222981320

H -9.87705790937432 -10.42658013785885 4.90977784053562

H -10.48726335792608 -10.57587986760667 3.31137907873254

H -7.57159472142330 -10.61691000785640 3.69851702293855

H -8.38592970544786 -12.08898531006028 4.14599557333420

H -9.48349440725774 -11.91482869794720 1.74249044667181

H -7.82997033201879 -12.52140729440985 1.91450520712408

H -8.84322279264486 -9.73281107410239 1.00076850426995

H -7.21855145391166 -9.85481970011887 1.45198808227100

H -8.74519595728613 -11.10431632574829 -0.83540435810691

H -7.28710154460731 -11.95715713479346 -0.36169020433946

H -7.77354671797339 -6.55150886381615 -5.21371357573711

H -11.87555612165474 -5.54445579092762 -6.17437561089626

H -9.32213580790969 -5.16431091318876 -6.51011534673766

H -11.29392612296596 -8.87522305533282 -2.19228053627676

H -11.17660774321233 -9.93790479241450 -3.53076529733582

H -13.30664111938887 -9.36042438700426 -1.63071396624578

H -16.40630372789257 -6.86836830496964 -2.26235387838827

H -15.26209354933148 -8.90679014689468 -4.86975023357260

H -16.23316261117957 -10.36792850686022 -4.45551319881757

H -13.74016773745084 -10.61936335334363 -3.69098959480028

H -14.93383601172054 -10.79854172715453 -2.45364916217628

H -7.63973270353118 -9.07975214897960 -1.24847813225345

H -6.01665093244429 -9.76112689305349 -0.73496100012552

H -5.93951207878183 -10.69035011623852 -4.04661268770969

H -5.27039128005133 -9.29053552706138 -3.13668330223577

H -8.63471091843155 -8.99900830288263 -2.91618277853155

121

energy: -182.740911228250 gnorm: 0.178038277305 xtb: 6.4.1 (afa7bdf)

C -6.24565847668370 -7.51759602412888 -5.09086513948610

C -4.92925940073709 -7.44252941318506 -5.27631187829965

C -4.04860555687367 -8.60949887198247 -5.30720518079064

C -4.72638417752186 -9.88946785500764 -5.43001109039772

C -6.03148400185879 -10.04918381262024 -5.17224639154101

C -6.91070064818845 -8.84893864171348 -4.94729075288072

C -7.36286157522111 -8.90987752067707 -3.44532707150129

C -6.76185999268721 -11.34963819840141 -5.12136918309527

C -7.49371867155102 -11.38312925639274 -3.77543497694978

C -8.28108964866978 -10.14041463986006 -3.35414361477846

C -8.62187219498074 -7.88215418123930 -1.46239344511837

C -7.89208941248473 -7.60804144446588 -2.79209719184936

C -8.73520307822309 -10.28903389557852 -1.90491843675376

C -9.58461433366901 -9.06569615485982 -1.53428105583418

C -10.22593820953950 -9.58855601152121 -0.21208835936668

C -9.64508286472141 -11.49543095860454 -1.68256557488122

C -10.54863804544624 -11.08156575587571 -0.49876227912743

O -2.84270575390707 -8.41138109510987 -5.47087190225008

H -7.90856532380766 -10.36135762773547 -1.18878939575899

C -10.63304553285976 -8.67502732388438 -2.57585526415284

C -11.52660066982062 -8.95249984476558 0.30952759784773

C -10.43406280282833 -12.06128795414918 0.66448486206961

O -8.62704497557210 -6.94671517105849 -3.78866063900331

C -7.95391352401891 -8.75610528110016 -6.07757965878399

F -6.16531026073140 -9.25502220596491 -2.78816543983928

H -9.09649738039509 -10.00809052844786 -4.06576808990228

O -9.31036952761520 -9.47908083073039 0.86367582442736

O -12.60191348079911 -9.53193235800284 0.39439181735488

N -11.29708509508666 -7.67681515912860 0.64442066873233

C -12.20454844441153 -6.81453797408683 1.36832396913899

C -12.85898232728528 -5.78024240004636 0.44883580204200

C -13.93243378716761 -6.45511848649013 -0.40147196077815

C -14.45478843659351 -5.51326843973957 -1.48431163024318

C -15.89350595966332 -5.84467599215392 -1.87599604794482

C -17.02007709064975 -5.61198682876848 -0.87157828619289

C -17.87528737894435 -4.35154274198057 -0.98381088832508

C -17.21186867540359 -3.25609706839084 -0.15153348246181

C -17.94415546654763 -1.92117632096312 -0.26353810082977

C -13.42954732989120 -2.78675574552058 -2.96358366042590

C -13.72181406106282 -3.30112271450145 -4.22761089142926

C -12.22685627994718 -3.07612251854948 -2.32659676294055

C -11.43005053466845 -4.08675440095618 -2.86247949751150

C -11.78153626567122 -4.71439821758723 -4.04617118779166

C -12.92271577751055 -4.31951248390089 -4.72462504577569

C -11.66918948543514 -2.62536943143493 -1.01090980493616

N -10.41845448090370 -3.35571766934909 -0.98684337168573

C -10.22079807523452 -4.19491490419511 -2.02980680054845

C -9.50331590366142 -3.09416874385743 0.10399256996347

C -9.02452816860267 -4.33433396416984 0.86271875037099

N -8.00821368297216 -4.17588344539711 1.77162215020366

C -7.25282611703516 -3.03305485259638 1.93531267830430

C -7.45419683349576 -1.88060648919095 0.99386691995643

C -8.29618802427686 -2.21370359457868 -0.23119038746983

O -9.41391712206432 -5.46706425301491 0.67879094022694

O -6.53782800208344 -3.00523105119175 2.91290112194050

O -9.22732705499266 -4.82362801117246 -2.38106520038081

C -17.09916257346639 -0.73836079458248 0.19372280282110

O -15.86432690621475 -0.63793195440034 -0.49950803180223

C -15.93257840695633 -0.23229755734435 -1.84181058123640

C -15.37968976081237 -1.24770914425528 -2.83870056365274

N -14.26090721752068 -1.83828243759214 -2.36731415261844

O -15.95478215685366 -1.48385887312551 -3.88652327911398

H -6.88056771422896 -6.65233770925267 -4.96607210162304

H -4.37432039606781 -6.51533511850860 -5.27770616011981

H -4.03687545244559 -10.68497195410141 -5.67789412756243

H -7.47562295280223 -11.46143561234611 -5.93851009343401

H -6.08723267545095 -12.19709977368872 -5.24194365412840

H -8.11520293003058 -12.26932957407823 -3.65522318580646

H -6.79660597070388 -11.59042176873517 -2.96075764911209

H -9.22634151039335 -7.00324452879730 -1.22584154403147

H -7.83857992022546 -8.11550504639356 -0.74054141152093

H -7.00893807255709 -6.96683616049990 -2.62182124788731

H -10.26492313315635 -11.51155315289967 -2.57821671123031

H -9.11926827030782 -12.44345920705318 -1.58881209982667

H -11.53438530617233 -11.06364390965536 -0.97218725463361

H -11.15651535230966 -9.58711793875878 -2.85582828022415

H -10.14255710396359 -8.40119042214963 -3.50381186535178

H -11.34429856637600 -7.96416500351188 -2.16960479319904

H -10.98341130248715 -11.75128079753274 1.54981238871785

H -9.38003288914791 -12.15113181433394 0.92466134581560

H -10.90900962542022 -13.00925600432405 0.41818219826258

H -8.70963241837798 -6.07339444407560 -3.38411657146427

H -8.35438706385145 -7.76064857261871 -6.22750572895764

H -7.37781973391636 -9.15915661511753 -6.90243893068556

H -8.83397236744648 -9.33687184996719 -5.81050954383209

H -9.69309441879036 -9.82608053507487 1.67867093701168

H -10.32118807102542 -7.41573151250220 0.65176024308204

H -12.97365447630706 -7.48698814191779 1.75673628091949

H -11.60913988480093 -6.38421145618210 2.17597811906717

H -13.36153781384076 -4.89841997557192 0.85192083630901

H -12.08083975747484 -5.42153504798320 -0.22840789623649

H -14.66747859292701 -6.71093205898128 0.36447989784166

H -13.50725091257435 -7.34165370195261 -0.87673235778626

H -13.73283135714792 -5.55150348083564 -2.30320865496645

H -14.43105810292354 -4.50650266129421 -1.06181050758934

H -16.18786567524895 -5.25406303828594 -2.74664927775700

H -15.94100653379382 -6.87788418403633 -2.22671293903994

H -17.68638895402747 -6.46979228075256 -0.75696852529889

H -16.58987628005113 -5.66338120692990 0.13134558952337

H -18.92851245133995 -4.49380173861430 -0.73422371953874

H -17.75445703159413 -3.95558813677640 -1.99450726104568

H -17.34194052978022 -3.52728335598797 0.89819806908356

H -16.22894924473499 -3.16112362577594 -0.61844662020427

H -18.85354679200159 -2.07002739988929 0.31968967121696

H -18.40503076904062 -1.85405805607304 -1.25017743953392

H -14.70491701074425 -3.04555586139165 -4.58680604301638

H -11.31690681052329 -5.59581713787337 -4.45799335101583

H -13.16295392849193 -4.73509413704878 -5.69033453243126

H -12.37027382770303 -2.81132524868485 -0.18982356992659

H -11.43880151769329 -1.55464066768244 -1.00694663594680

H -10.05439598631131 -2.61975140568444 0.92644397544569

H -7.72567377254465 -4.94785024441070 2.36999616574068

H -6.47619200766357 -1.45635039137492 0.74848791095938

H -7.97308485438677 -1.09248929634983 1.53929800219538

H -7.79766105952553 -2.71096285265099 -1.06266271938360

H -8.61721717044472 -1.28137642587609 -0.69712924810648

H -17.66997542905592 0.19957517077317 0.25869649740860

H -16.65309824348073 -0.99045376587416 1.16570836783725

H -15.30041209900933 0.66759127662634 -1.83740198367397

H -16.94622834732273 0.09013417018368 -2.12743489786474

H -14.11554629008293 -1.50691988668517 -1.41868144714124
