# Supplementary material for: Harnessing PROTAC technology to combat stress hormone receptor activation
Source: Nat Commun. 2023 Dec 9;14:8177. doi: 10.1038/s41467-023-44031-2 (PMC10710461; doi:10.1038/s41467-023-44031-2)
Supplement: Supplementary file 6 — Reporting Summary [file 41467_2023_44031_MOESM6_ESM.pdf]

nature portfolio

Corresponding author(s): NCOMMS-23-09734 Katharina Gapp

Last updated by author(s): Nov 1, 2023

Reporting Summary

Nature Portfolio wishes to improve the reproducibility of the work that we publish. This form provides structure for consistency and transparency in reporting. For further information on Nature Portfolio policies, see our Editorial Policies and the Editorial Policy Checklist.

Statistics

For all statistical analyses, confirm that the following items are present in the figure legend, table legend, main text, or Methods section.

n/a	Confirmed	
☐	☒	The exact sample size (*n*) for each experimental group/condition, given as a discrete number and unit of measurement
☐	☒	A statement on whether measurements were taken from distinct samples or whether the same sample was measured repeatedly
☐	☒	The statistical test(s) used AND whether they are one- or two-sided<br>*Only common tests should be described solely by name; describe more complex techniques in the Methods section.*
☒	☐	A description of all covariates tested
☐	☒	A description of any assumptions or corrections, such as tests of normality and adjustment for multiple comparisons
☐	☒	A full description of the statistical parameters including central tendency (e.g. means) or other basic estimates (e.g. regression coefficient) AND variation (e.g. standard deviation) or associated estimates of uncertainty (e.g. confidence intervals)
☐	☒	For null hypothesis testing, the test statistic (e.g. *F*, *t*, *r*) with confidence intervals, effect sizes, degrees of freedom and *P* value noted<br>*Give P values as exact values whenever suitable.*
☒	☐	For Bayesian analysis, information on the choice of priors and Markov chain Monte Carlo settings
☐	☒	For hierarchical and complex designs, identification of the appropriate level for tests and full reporting of outcomes
☐	☒	Estimates of effect sizes (e.g. Cohen's *d*, Pearson's *r*), indicating how they were calculated

Our web collection on statistics for biologists contains articles on many of the points above.

Software and code

Policy information about availability of computer code

Data collection	The code underlying the bioinformatic analysis, including processed data objects, are available on the following GitHub repositories: https://github.com/ETHZ-INS/MG_A549 (main differential analysis) and https://github.com/ETHZ-INS/Glucorticoid-protacs (downstream questions and specific figures). GR-ChIP-Sequencing data were accessed from the ENCODE portal(McDowell et al., 2018).
Data analysis	The code underlying the bioinformatic analysis, including processed data objects, are available on the following GitHub repositories: https://github.com/ETHZ-INS/MG_A549 (main differential analysis) and https://github.com/ETHZ-INS/Glucorticoid-protacs (downstream questions and specific figures). GR-ChIP-Sequencing data were accessed from the ENCODE portal(McDowell et al., 2018). salmon 1.7.0<br>tximport 1.18.0<br>sva 3.44.0<br>edgeR 3.38.1<br>epiwraps 0.99.50<br>FragPipe 18.0<br>MSFragger 3.5<br>Philosopher 4.4.0<br>prolfqua 1.1.2

For manuscripts utilizing custom algorithms or software that are central to the research but not yet described in published literature, software must be made available to editors and reviewers. We strongly encourage code deposition in a community repository (e.g. GitHub). See the Nature Portfolio guidelines for submitting code & software for further information.

Data

Policy information about availability of data

All manuscripts must include a data availability statement. This statement should provide the following information, where applicable:

- Accession codes, unique identifiers, or web links for publicly available datasets
- A description of any restrictions on data availability
- For clinical datasets or third party data, please ensure that the statement adheres to our policy

Sequencing data have been deposited at GEO; the accession number is: GSE229084. The remaining data are deposited at the ETH research collection https://doi.org/10.3929/ethz-b-000603617, which will be lifted upon publication. The identical excel file is supplied as source data.

Research involving human participants, their data, or biological material

Policy information about studies with human participants or human data. See also policy information about sex, gender (identity/presentation), and sexual orientation and race, ethnicity and racism.

Reporting on sex and gender	na
Reporting on race, ethnicity, or other socially relevant groupings	na
Population characteristics	not applicable
Recruitment	not applicable
Ethics oversight	not applicable

Note that full information on the approval of the study protocol must also be provided in the manuscript.

Field-specific reporting

Please select the one below that is the best fit for your research. If you are not sure, read the appropriate sections before making your selection.

☒ Life sciences ☐ Behavioural & social sciences ☐ Ecological, evolutionary & environmental sciences

For a reference copy of the document with all sections, see nature.com/documents/nr-reporting-summary-flat.pdf

Life sciences study design

All studies must disclose on these points even when the disclosure is negative.

Sample size	Sample size was not predetermined via calculation due to the absence of any data on effects of a GR targeting PROTAC, but used a minimum of 3 replicates in cases where the number of experimental conditions did not allow to allow further simultaneous handling. This minimum number was chosen to allow for statistical analysis and was guided by similar experimental designs evaluating the efficiency of PROTACs. We included more replicates, if experimentally feasible to handle more samples simultaneously. Information on the number of replicates for each experiment are provided in the manuscript.
Data exclusions	No replicates were excluded from the reporting, apart from animals where the drug dosing partially failed (reflux during subcutaneous injection)
Replication	We have not indication for data not being reproducible. In the fractionation experiments Western blots were repeated to allow a more balanced distribution of treatment groups and results were not affected. Intraperitoneal injection experiments of KH-103 was reproduced in an independent set of animals once.
Randomization	Samples were allocated randomly for in vitro experiments and matched for weight in in vivo experiments.
Blinding	Experimenter was blinded were possible . In in vitro studies using different dosages all wells received PROTAC and hence blinding was not possible/indicated. Researchers doing animal work were not identical to experimenters carrying out associated Westernblots/Elisas, and hence partial blinding could be achieved of secondary experimenter.

Reporting for specific materials, systems and methods

We require information from authors about some types of materials, experimental systems and methods used in many studies. Here, indicate whether each material, system or method listed is relevant to your study. If you are not sure if a list item applies to your research, read the appropriate section before selecting a response.

Materials & experimental systems

n/a	Involved in the study
	☒ Antibodies
	☒ Eukaryotic cell lines
☒	☐ Palaeontology and archaeology
	☒ Animals and other organisms
☒	☐ Clinical data
☒	☐ Dual use research of concern
☒	☐ Plants

Methods

n/a	Involved in the study
☒	☐ ChIP-seq
☒	☐ Flow cytometry
☒	☐ MRI-based neuroimaging

Antibodies

Antibodies used

The following antibodies were used: GR (G-5, Santacruz sc-393232, 1:100), MR (clone 6G1, Merck MABS496, 1:1000), alpha-tubulin (11H10, cell signaling 2125S, dilution 1:1000), GAPDH (ABS16, Merck Millipore, 1:1000), HA (C29F4, cell signaling #3724, 1:1000), Calpain (Abcam ab28258, 1:1000), VDAC1 (Abcam ab15895, 1:1000), IL-6 (Abcam ab259341, 1:1000), H3 (Abcam ab1791, 1:1000), and GFP (Abcam, ab290, 1:1000) MAP2 antibody (Chicken monoclonal anti-Map2 PA1-16751; Thermo Fisher Scientific, 1:2000) .
Secondary antibodies included: goat anti-mouse IgG antibody (Merck Millipore AP308P, (H+L) HRP conjugate, 1:20'000) and goat anti-rabbit IgG (Merck Millipore AP307P, (H+L) HRP conjugate, 1:20'000).
Cy3 Goat anti-mouse against GR, Jackson ImmunoResearch 115-165-003, 1:300 and goat anti-chicken Alexa488 against MAP2, Thermo Fisher Scientific A-11039, 1:1000)

Validation

All antibodies used are commercially available and were validated by their manufacturer as listed below:
GR (G-5, Santacruz sc-393232, 1:100) validated by manufacturer for Western blot analysis of GR expression in Hep G2 (A), Jurkat (B) and A-431 (C) whole cell lysates, A-431 nuclear extract (D) and mouse brain tissue extract (E) and for immunoperoxidase staining of formalin fixed, paraffin-embedded human appendix tissue showing cytoplasmic and membrane staining of glandular cells (https://www.scbt.com/p/gr-antibody-g-5) and referenced by Bachman 2018 https://pubmed.ncbi.nlm.nih.gov/29343704/, Liang 2021 https://www.nature.com/articles/s41419-021-03982-4.
MR (clone 6G1, Merck MABS496, 1:1000) validated by manufacturer by Western Blotting in M1MR cell lysate and referenced by Shibata, S., et al. (2013) Cell Metab. 18(5):660-671
alpha-tubulin (11H10, cell signaling 2125S, dilution 1:1000) validated by manufacturer by Western blot analysis of extracts from C6, COS-7, NIH/3T3 and HeLa cells and referenced by Dorn 2018 https://pubmed.ncbi.nlm.nih.gov/29862142/
GAPDH (ABS16, Merck Millipore, 1:1000) validated by manufacturer by Western Blotting Analysis using A 1:1,000 dilution from a representative lot detected GAPDH in NIH3T3 and L6 cell lysates and referenced by Yamasaki 2015 https://pubmed.ncbi.nlm.nih.gov/25181483/ and Stefanovic 2021 https://www.ncbi.nlm.nih.gov/pmc/articles/PMC8261018/
HA (C29F4, cell signaling #3724, 1:1000) validated by manufacturer using Western blot analysis of extracts from HeLa cells, untransfected or transfected with either HA-FoxO4 or HA-Akt3, using HA-Tag (C29F4) Rabbit mAb and referenced by Hwang 2020 https://www.ncbi.nlm.nih.gov/pmc/articles/PMC7558343/
Calpain (Abcam ab28258, 1:1000) validated by manufacturer by Western Blot using Anti-Calpain 1 antibody (ab28258) at 1:1000 dilution + Calpain 1 at 10 µg *there is no publication using this in any of our cell types, but it has been referenced 5 times for Western Blot as provided by CiteAb https://www.citeab.com/antibodies/717152-ab28258-anti-calpain-1-antibody?des=04046e7db69cadc0*
VDAC1 (Abcam ab15895, 1:1000) validated by manufacturer by Western Blot in HeLa, A431,Jurkat, PC12, Rat kidney and HEK293 whole cell lysates, Mouse heart, kidney, skeletal muscle, spinal cord tissue lysate and rat brain tissue lysate and referenced by Yu 2021 https://www.ncbi.nlm.nih.gov/pmc/articles/PMC8532385/
IL-6 (Abcam ab259341, 1:1000) validated by manufacturer by Western Blot in RAW264.7 treated with 0.1 µg/ml lipopolysaccharide (LPS) then with 1 µg/ml Brefeldin A (BFA), HUVEC treated with 0.5 µg/ml LPS, then with 0.3 µg/ml BFA, NR8383 treated with 0.1 µg/ml LPS, then with 1 µg/ml BFA *there is no reference using our cell types but it has been referenced 33 times for Western Blot as provided by CiteAb https://www.citeab.com/antibodies/12098295-ab259341-recombinant-anti-il-6-antibody-epr23819-1?des=29b9257e878f327a*
H3 (Abcam ab1791, 1:1000) validated by manufacturer by Wstern Blot in A431 whole cell lysate, Jurkat whole cell lysate, HEK293 whole cell lysate and referenced by Denisenko 2018 https://www.ncbi.nlm.nih.gov/pmc/articles/PMC5990391/
MAP2 antibody (Invitrogen, PA1-16751, 1:2000) validated by manufacturer by immunofluorecence staining in cortical neuron-glial cell culture from E20 rat. Samples were incubated in MAP2 polyclonal antibody using a dilution of 1:10000 and referenced by Antrobus 2021 https://pubmed.ncbi.nlm.nih.gov/33483729/

Eukaryotic cell lines

Policy information about cell lines and Sex and Gender in Research

Cell line source(s)

HEK293 cells- provided by the Schratt lab
N2a cells - provided by the Schratt lab
A549 cells - purchased commercially. ATCC (ATCC-CCL-185)

Authentication

A549 cells were directly purchased from ATCC (ATCC-CCL-185). N2a and HEK293 cells were provided by Prof. Schratt lab and were originally purchased and authenticated at the ATCC as well. A549 and HEK293 were negatively tested for mycoplasma contamination and authenticated using Karyotyping and STR profiling (https://www.atcc.org/products/ccl-185#detailed-product-information, https://www.atcc.org/products/crl-1573). N2a were negatively tested for mycoplasma and authenticated by Karyotyping (https://www.atcc.org/products/ccl-131).

Mycoplasma contamination	The cell lines were not retested for Mycoplasma contamination in our lab.
Commonly misidentified lines (See ICLAC register)	no commonly misidentified lines were used

Animals and other research organisms

Policy information about studies involving animals; ARRIVE guidelines recommended for reporting animal research, and Sex and Gender in Research

Laboratory animals	We used C57/Bl6J mice 2-3 months of age housed in individually ventilated cages. Housing temperature was set to 21°C and relative humidity to 55%. For primary culture pregnant Wistar rats were purchased from Janvier directly. Pups were sacrificed at embryonic day 18.
Wild animals	No wild type animals were used in this study.
Reporting on sex	Data provided are from male mice only, yet while stress hormone signaling is known to differ between males and females - the expression of the glucocorticoid receptor is expectedly equal. Hence we assume that females would respond identically. Due to the limited availability of our compound we were constrained in the number of animals we were able to assay. Our aim was further a proof of principle experiment and not the elucidation of sex specific responses to our compound.
Field-collected samples	No field collected samples were used in this study.
Ethics oversight	The cantonal veterinary office of Zürich approved our experiments. The license number is license number: ZH067/2022. For Primary culture the cantonal office of Basel approved experiments under license: 2358.

Note that full information on the approval of the study protocol must also be provided in the manuscript.
